# Supplementary material for: Ultralight crystalline hybrid composite material for highly efficient sequestration of radioiodine
Source: Nat Commun. 2024 Feb 10;15:1278. doi: 10.1038/s41467-024-45581-9 (PMC10858966; doi:10.1038/s41467-024-45581-9)
Supplement: Supplementary file 1 — Supplementary information [file 41467_2024_45581_MOESM1_ESM.pdf]

Supplementary Information

Ultralight Crystalline Hybrid Composite Material for Highly Efficient Sequestration of Radioiodine

Sahel Fajal,[1] Writakshi Mandal,[1] Arun Torris,[2] Dipanjan Majumder,[1] Sumanta Let,[1] Arunabha Sen,[1] Fayis Kanheerampockil,[2] Mandar M. Shirolkar,[3,4] and Sujit K. Ghosh[1,5]*

[1]Department of Chemistry, Indian Institute of Science Education and Research (IISER) Pune, Dr. Homi Bhaba Road, Pashan, Pune 411008, India. Phone: +91 20 2590 8076

[2]Polymer Science and Engineering Division, CSIR-National Chemical Laboratory, Dr. Homi Bhabha Road, Pune 411008, India.

[3]Advanced Bio-Agro Tech Pvt. Ltd, Baner, Pune 411045, India.

[4]Norel Nutrient Bio-Agro Tech Pvt. Ltd, Baner Pune 411045 India.

[5]Centre for Water Research (CWR), Indian Institute of Science Education and Research (IISER) Pune, Dr. Homi Bhaba Road, Pashan, Pune 411008, India.

*Corresponding author. E-mail: sghosh@iiserpune.ac.in

Supplementary Methods

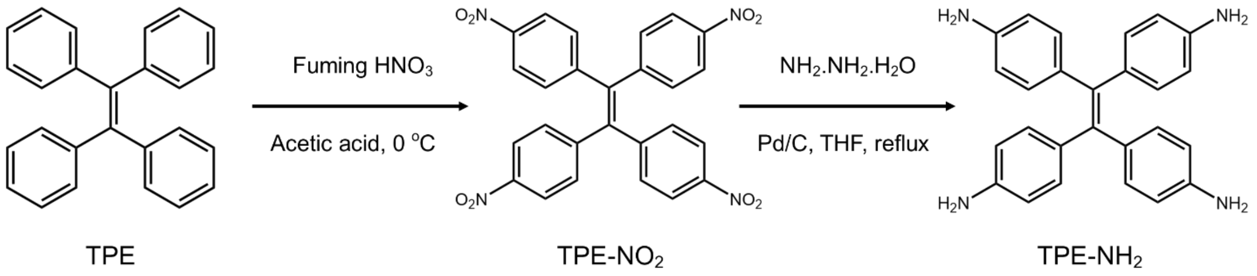

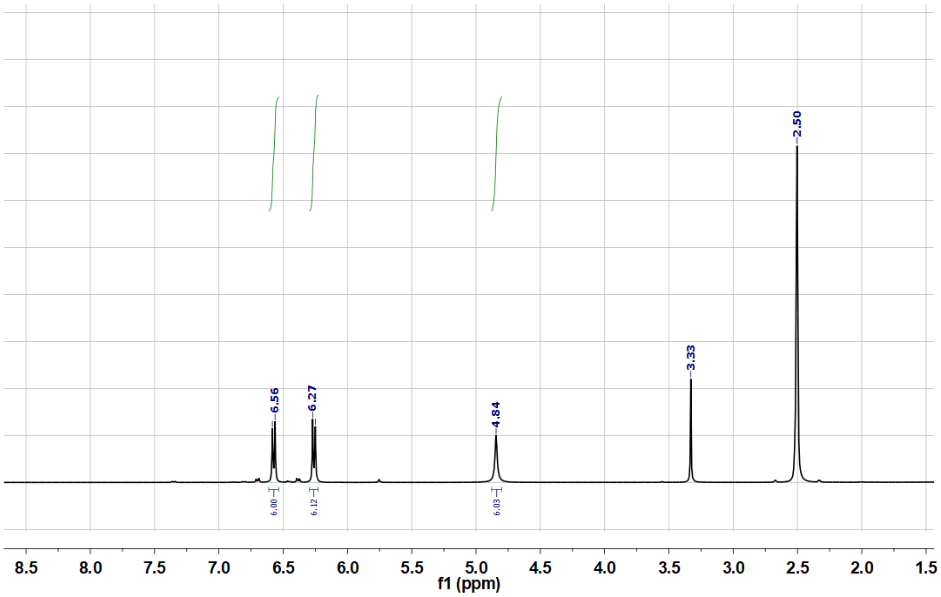

Supplementary Figure 1: ^{1}H NMR spectrum of TPE-NH_2 in DMSO-d_6.

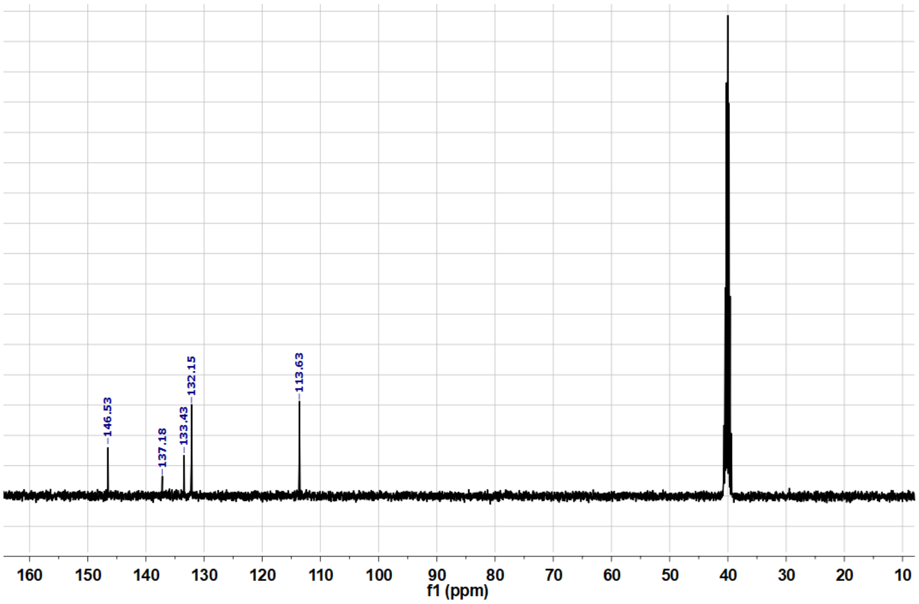

Supplementary Figure 2: ^{13}C NMR spectrum of TPE-NH_2 in DMSO-d_6.

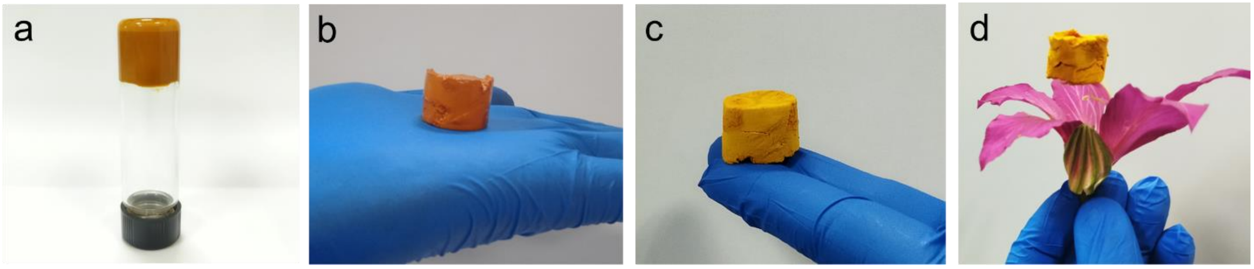

Supplementary Figure 3: Digital images of the hybrid composite material (IPcomp-7). (a, b) The as-synthesized wet-gel form of IPcomp-7. (c, d) Ultra-light weight nature of the crystalline hybrid aerogel.

Digestion process and ^{1}H NMR analysis of IPcomp-7: Almost, 30 mg of NH_2-Zr(IV)-MOP/COF composite aerogel material (IPcomp-7) was digested in ~2 mL of 1 mol/L NaOH solution over ~6 hours. Then, 2 mL of DI water was added to the mixture and the overall solution was then sonicated properly for a certain time. Thereafter, the solution was left standing for one day for precipitating. Further, the precipitate was removed by a membrane filtration (0.2 µm pores) to give a clear solution containing the corresponding organic ligands of metal-organic polyhedron, 2-aminoterepthalate ($2ATA^{2-}$). Now, in order to remove the solvent molecules, the mixture was subjected to undergo dynamic vacuum at 80 °C overnight to obtain the solid powder with very low yield. After that the powder was dispersed in 1.2 mL of D_2O and followed by the addition of few drops of 40 wt% NaOD, which result a clear solution. This clear solution was than analyzed for ^{1}H NMR.

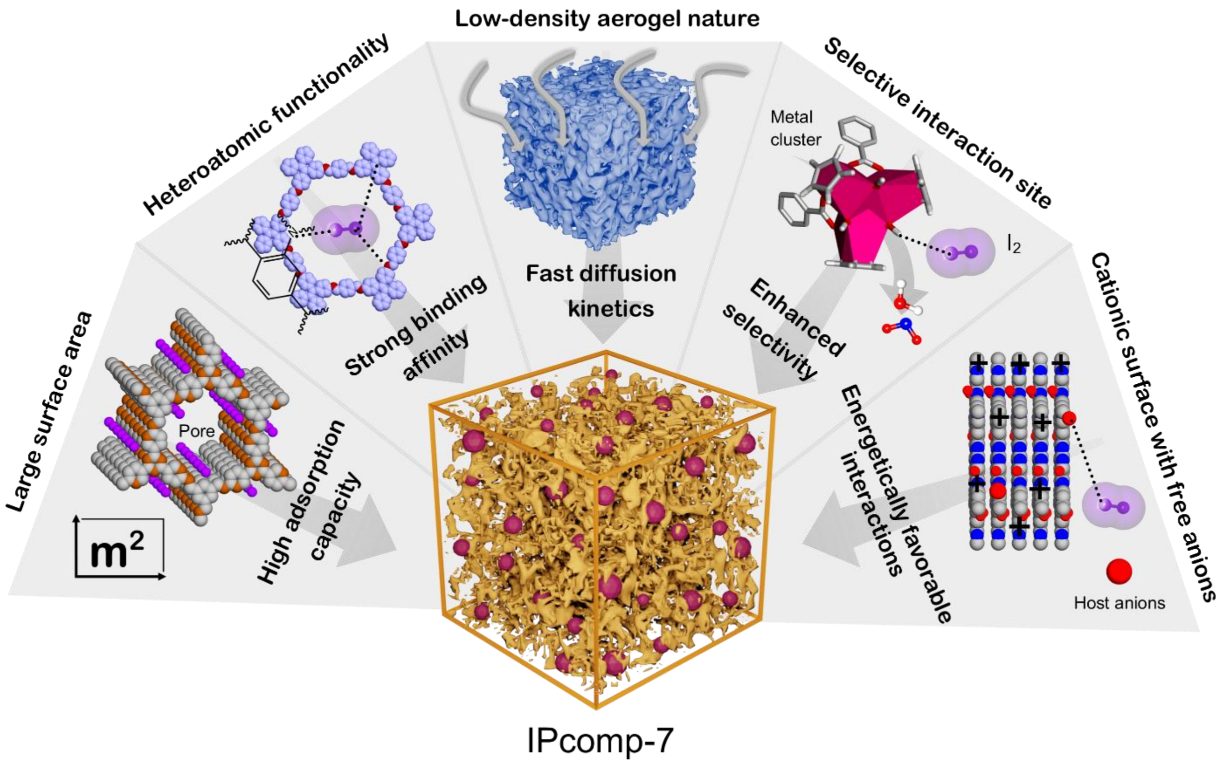

Supplementary Figure 4: Schematic representation of different strategies involved in the design of hybrid composite material (IPcomp-7) as an effective adsorbent for high performance iodine sequestration.

Supplementary Note 1

General Consideration: The aqueous or organic solution of iodine (I_2) or triiodide (I_3^-) were prepared by dissolving a certain amount of solid iodine or iodine-potassium iodide (I_2/KI) into deionized water or n-hexane solution and the different concentrations were obtained by diluting the stock solution with the proper amount of distilled water/n-hexane unless otherwise indicated. The concentrations of I_2/I_3^- during all the experiments were detected by UV-vis spectroscopic analysis. All the adsorption experiments were performed at ambient conditions. All the data has been collected three/two times through UV-vis analysis to plot the final adsorption results.

Vapor phase static iodine uptake studies: IPcomp-7 (10 mg) was weighted in a small glass vial, and this vial was exposed to molecular iodine for 24 h at 75 °C in a closed system. After the corresponding adsorption time, the glass vial was taken out, cooled to room temperature, and weighed. Iodine uptake capacity of the compound was calculated by the weight difference of glass vials before and after adsorption, using the formula:

$$w = \frac{(m_2 - m_1)}{m_1} \quad \text{..............(1)}$$

Where, w ($g.g^{-1}$) is the adsorption capacity of iodine uptake, m_1 (g) and m_2 (g) are the mass of compounds with the glass vial before and after being exposed to iodine vapor. The values of uptake capacity are the average values of at least three experiments.

Recyclability test in static condition: To study the recyclability for iodine-loaded material in static system we followed a reported protocol with slight modification. We first investigated the desorption efficiency. A certain amount of iodine-loaded compound (I_2@IPcomp-7) with maximum adsorption capacity was taken in an open glass vial and heated at ~150 ºC for a certain period of time. The iodine desorption efficiency was calculated by weight difference: Desorption efficiency = *(w3 – w2)/w1* × 100%, where w1 (g) is the weight of iodine in I_2@IPcomp-7 before desorption. w2 (g) is the weight of I_2@IPcomp-7 after desorption, and w3 (g) is the weight of I_2@IPcomp-7 before desorption. For the recyclability test, the I_2@IPcomp-7 powder was heated at ~150 ºC for ~16 h to achieve the complete desorption and used for the next cycle of iodine adsorption.

Retention ability test: To study the adsorption stability for iodine-loaded material in static system, a certain amount of iodine-loaded material (I_2@IPcomp-7) was taken in an open glass vial, and the vial

was placed in an empty large bottle, which was placed at room temperature and ambient pressure. After one day, the weight of the vial containing iodine-adsorbed material was recorded and the vial was placed back into the empty large bottle. The large bottle was opened to further continue the experiment for next 7 days.

Iodine (I_2) release studies in hexane: Time-dependent UV-Vis measurements were carried out in n-hexane to record the I_2 releasing efficiency from the I_2@IPcomp-7. In a typical experiment, 2 mg of I_2@IPcomp-7 was immersed in ~4 mL of n-hexane in a reaction vial with mild shaking. The UV-Vis adsorptions were recorded by periodically removing a 2 mL of n-hexane solution from the mother solution and then transferred back.

Vapor phase dynamic iodine uptake studies: The gaseous iodine adsorption in dynamic system was performed in a home-built setup. 20 mg IPcomp-7 was packed into a glass tube with glass wool filling the void space of both ends, which served as the adsorption cell. The temperature control system was set to 75 °C, and valve 1 and valve 2 were opened and valve 3 was closed. A nitrogen flow (10 cm^3/min) passed through the iodine vapor generator to blow the iodine vapor into the hybrid composite material in the adsorption cell. The flow rate of I_2 gas was about ~41±2 mg/h, determined through trial and error. The effluent from the adsorption cell was treated with 0.1 M NaOH solution in outlet. The adsorption capacity for iodine was calculated from the weight difference of compound containing glass tubes before and after adsorption.

Recyclability test in dynamic condition: To study the recyclability for iodine-loaded compound in dynamic system, valve 1 and valve 2 were closed and valve 3 was opened. The iodine- loaded compound with maximum adsorption capacity in the adsorption cell was heated at ~150 ºC for 16 h with a nitrogen flow (10 cm^3/min) to achieve the complete desorption, Then, valve 1 and valve 2 were opened and valve 3 was closed, and the temperature control system was set to 75 °C for the next cycle of iodine adsorption.

Aqueous phase static molecular iodine (I_2) capture studies: To study the iodine adsorption performance in aqueous solution system, we first prepared the molecular iodine (I_2) solutions in water. To prepare the saturated iodine aqueous solution, an excess of iodine was added into 1 L deionized water, and after stirred for ~6 h, the mixture was filtered to remove the undissolved iodine. The iodine concentration in the saturated iodine aqueous solution is about 300 ppm.

Aqueous phase static triiodide (I_3^-) capture studies: The I_3^- solutions were freshly prepared before experiments by mixing 300 mg I_2 and 600 mg KI in 20 mL deionized water sonication. The iodine concentration in this I_2/KI aqueous solution is 15000 ppm.

Static iodine (I_2) capture studies in hexane: 3000 mg of iodine was dissolved in 1 L n-hexane to prepare the n-hexane solution of iodine (3000 ppm).

Solution phase adsorption kinetic studies: For the time-dependent study for I_3^- removal, we took 3 mL of 100 ppm stock aqueous I_3^- solution in a cuvette. We recorded the initial absorbance value with the help of UV−visible (UV−vis) spectroscopy. Then, 3 mg of IPcomp-7 was added to the cuvette. After treating the compound, we recorded the absorbance spectra of the supernatant solution at regular time intervals. We calculated the % removal data of I_3^-, decrease in the concentration of the I_3^- vs time, and uptake of I_3^- from this study using the following equations:

$$Dt = \frac{(C_0 - C_{-L})}{C_0} x 100\% = \frac{(A_0 - A_t)}{A_0} x\ 100\% \quad \text{.........(2)}$$

$$\frac{C_0 - C_t}{C_0} = \frac{A_0 - A_t}{Ao} \quad \text{..............................(3)}$$

$$C_t = C_0\ x\ [1 - (A_0 - A_t/A_0)] \quad \text{.................................(4)}$$

$$Q_t = (C_t - C_0) x \frac{v}{m} \quad \text{..................................(5)}$$

D_t is the exchange capacity, C_0 and A_0 are the initial concentration and absorbance of the I_3^- solution, respectively, and C_t and A_t are the concentration and absorbance of the I_3^- solution at specific times, respectively. Q_t is the uptake amount in time t in mg g^{-1}, V is the volume of the solution in mL, and m is the mass of the compound in g. The kinetics data were fitted to a pseudo-second-order kinetic model using the following equation.

Pseudo-second-order model fitting: To investigate the kinetic adsorption behavior towards iodine, pseudo-second-order kinetic model was used to evaluate the adsorption data, and its linear form can be expressed as follow:

$$\frac{t}{Qt} = \frac{1}{k_2 Q_e^2} + \frac{t}{Q_e} \qquad \text{.............................(6)}$$

$$Q_t = \frac{Q_e^2 k_2 t}{1+Q_e k_2 t} \qquad \text{.............................(7)}$$

Where, Q_t and Q_e represent the adsorbed amount (mg.g^{-1}) at time and at equilibrium t (min), respectively, k_2 represent the Pseudo-second-order rate constant of adsorption (g mg^{-1} min^{-1}). The experimental data was fitted using Pseudo-second-order kinetic model, and k_2 is the correlation coefficient of the fitting curve.

Iodine uptake capacity studies: 5 mg of IPcomp-7 were kept in contact with 5 mL of I_3^- solutions bearing different concentrations (15000 to 50 ppm solution) for 24 h under stirring conditions. After 24 h, compounds were filtered out, and the filtrate was analyzed by UV−vis studies. The absorbance was recorded by diluting the solution, and the uptake amount was calculated from the initial and final absorbance value of the filtrate using equations (2−5).

Langmuir model, $Q_e = Q_m.C_e/(K_d+C_e)$; Where, C_e(ppm) and Q_e(mg g^{-1}) are the I_3^- concentration at equilibrium and amount of I_3^- adsorbed at equilibrium respectively. Q_m (mg g^{-1}) is the maximum amount of I_3^- per mass of adsorbent to form a complete monolayer. K_d (mg L^{-1}) is a constant to the affinity of the binding sites.

Freundlich model, $Qe = K_f.C_e^{1/n}$; Where, K_f and 1/n are the Freundlich model constant, indicating capacity and intensity of adsorption respectively.

Distribution coefficient (K_d) value calculation: The distribution coefficient (K_d) value as used for the determination of the affinity and selectivity of sorbents for triiodide (I_3^-), is given by the equation:

$$k_d = \left(\frac{C_0 - C_e}{C_e}\right) x \frac{v}{m} \qquad \text{...........................(8)}$$

Where, v is the volume of the testing solution (mL), m is the amount of solid adsorbent (g), C_o is the initial concentration of I_3^-, and C_e is the equilibrium concentration of I_3^-.

Selectivity test in presence of other competing ions: For testing the influence of competing ions, in this study, 2 mg each of IPcomp-7 was kept in contact separately with a binary solution containing 1 mL of I_3^- solution and 1 mL each of various competing anions solution (NO_3^-, Cl^-, Br^-, ClO_4^-, SO_4^{2-}) with equal concentration as well as ~100-fold excess for 24 h under stirring conditions. Then, after 24 h, the compounds were filtered, and the filtrate was analyzed by UV−vis studies using the protocol discussed above. The efficiency of the capture process in the presence of competing anions was measured with respect to a blank where 1 mL of water is used instead of the competing anion solution. The absorbance was recorded by diluting the solution, and the uptake amount was calculated from the initial and final absorbance value of the filtrate using equations 2−5. The iodine uptake in the presence of a mixture of competing ions was also carried using the same protocol.

I_3^- capture study in different water systems: A stock solution of 100 ppm I_3^- was prepared using different water matrix, such as seawater, river water, lake water, etc. Then, 5 mg of IPcomp-7 was kept in contact with 5 mL of I_3^- ion those water stock solution for 12 h under stirring conditions. After 12 h, compounds were filtered out, and the filtrate was analyzed by UV−vis studies. The absorbance was recorded by diluting the solution, and the uptake amount was calculated from the initial and final absorbance value of the filtrate using equations 2−5.

Dynamic column-based flow-through I_2 capture study in water: We conducted a dynamic column-based flow-through I_2 capture experiment with the flow of saturated iodine aqueous solution. IPcomp-7 was packed into a glass column with addition of cotton, and the saturated iodine aqueous solution was passed through the cell with a flow rate of 0.75 $mL.min^{-1}$. The color of IPcomp-7 material gradually became darker when iodine was adsorbed. The filtrate was clear, and the iodine concentrations of filtrate and the original solution were detected by UV-Vis spectrophotometer (Supplementary Figure-73).

Dynamic column-based flow-through I_3^- capture study in water: To check the continuous I_3^- capture efficiency by the hybrid aerogel material (IPcomp-7), a column exchange-based flow-through experiment was conducted by packing the hybrid aerogel inside a column as bed. After that, to mimic the ground water system different volume of stock ~100 ppm of I_3^- aqueous solution along with ~100-fold excess concentration of other interfering anions (such as NO_3^-, Cl^-, Br^-, ClO_4^-, SO_4^{2-}) were mixed. Thereafter, this mixture was passed through the column bed with a flow rate of 0.5 mL/min, which was controlled by a water-flow-regulator, and the drops per minute was recorded with the help of a stopwatch. A bed-volume of ~4.2-~4.5 mL of column eluted solution was collected in a different

respective container. The concentration of I_3^- in these solutions were then analyzed through UV-vis analysis. The recyclability test of the column exchanged-based I_3^- capture study was performed by washing the column with pure water, followed by excess n-hexane and saturated NaCl solution after each cycle. The filtrate of each cycle was collected at the bottom of the column (Supplementary Figure-75). Before and after capture studies, the concentration of I_3^- solutions were analyzed through UV-vis analysis.

Recovery of solid iodine by heating: The recovery of solid iodine was performed in a home-built setup as shown in Supplementary Figure-77. A certain amount of iodine loaded (captured through vapor phase) compound (I_2@IPcomp-7) was heated in a closed camber at ~150 ^oC under the flow of nitrogen. The iodine vapor generated from the compound upon heating was further subjected to condense in a separate glass flask, which was dipped in an ice-cool bath for a period of time. After that the glass flask was removed from the system. It was found that solid crystals of iodine was condensed on the surface of the glass flask. These solid iodine crystals were then measured and recovered in a separate storage vial.

Recovery of solid iodine by treating in pentane: A certain amount of iodine loaded (captured through vapor phase) compound (I_2@IPcomp-7) was treated with excess amount of pentane. Upon this treatment the color of pentane solution was become dark violet color, which indicated liberation of large amount of iodine from I_2@IPcomp-7. Thereafter, this iodine containing pentane was transferred into a glass petri dish, which was further kept at room temperature for evaporation for few minutes. After complete evaporation of pentane a large amount of solid iodine was found to crystalize on the top of the glass dish. This recovered iodine crystals were then stored in a separate glass vial (Supplementary Figure-78).

Supplementary Note 2

Determination of electrostatic surface potential (ESP), binding sites and binding energy of different interactions:

The molecular-level interactions between the amino functionalized cationic Zr(IV)-MOP and repeating unit of COF along with iodine/polyiodide species were simulated using Discovery Studio 2017 (Accelrys) (BIOVIA, Dassault Systèmes Version: V16.1.0.15350).[57] The DFT simulation was performed at fine quality calculation level with SCF density convergence of 1×10^{-6}. At first, using DMOL3 and B3LYP hybrid function the single unit of the MOP and repeating unit of the COF was fully geometry relaxed with following parameters, (a) maximum displacement to 0.05 Å, (b) octapole auxiliary density, (c) thermal orbital occupation to 0.005, (d) SCF charge mixing to 0.2, (e) SCF DIIS to 6 pulay, (f) orbital Multiplicity factor to Auto mode (ground spin state determine using spin-unrestricted calculations) and (g) double numeric plus polarizing (DNP+) basis set with water as solvent. Using simulated annealing technique structural simulation on the geometry relaxed MOP and COF structure was realized to find a feasible interaction site of the I_2/I_3^- within the relaxed MOP and COF unit. The calculations were performed with following parameters, (a) temperature: 298 K, (b) forcefield: Universal, (c) Ewald & Group electrostatic summation method, (d) the number temperature cycles: 3, (e) 15000 Monte Carlo steps per cycle and (f) conformer, rotate, translate and regrow ratio to 1. The electrostatic potential (ESP) on the van der Waals (VDW) surfaces of MOP and COF unit was derived from ground-state electron density of the system with isodensity = 0.001 a.u.

The Static binding energies (ΔE) at 0 K in vacuum were calculated using the following expression

$$\Delta E = E_{(MOP+I2/I3-)} - E_{(MOP+)} - E_{I2/I3-}$$

$$\Delta E = E_{(COF+I2/I3-)} - E_{(COF+)} - E_{I2/I3-}$$

Where, E_x refers, respectively, to the total energies of the MOP + I_2/I_3^- or COF + I_2/I_3^- complex, the charged MOP+ and COF+ alone, and the I_2/I_3^- molecule/anions respectively.

Supplementary Figures

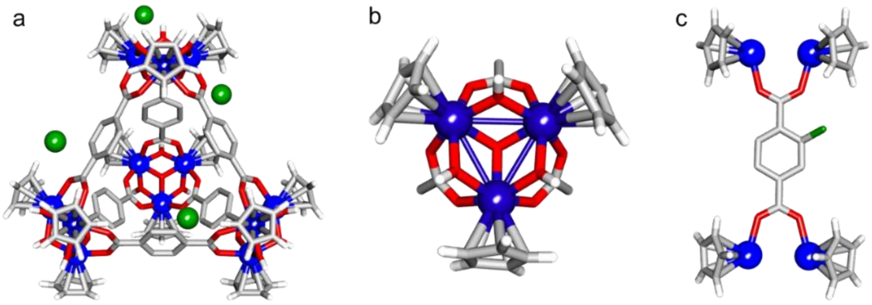

Supplementary Figure 5: Structural features of the (a) NH_2-Zr(IV)-based cationic metal-organic polyhedra (MOPs), (b) Zr(IV)-based secondary building unit (SBU), (c) Organic linker (2-aminoterepthalate) of the MOP. (Color code: zirconium: yellow, oxygen: red, carbon: gray, hydrogen: white, chlorine: green).

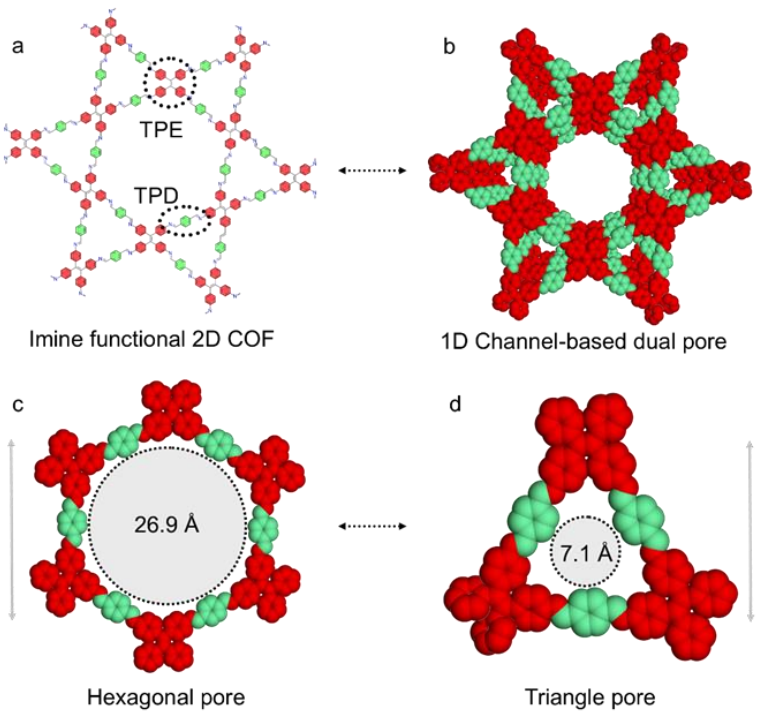

Supplementary Figure 6: Structural features of the covalent-organic framework (COF): (a) Chemical structure of the TPE core-based imine functional 2D COF. (b) Packing structure of 1D channel-based dual pore TPE-COF. (c, d) Hexagonal and triangle pores of the COF, respectively.

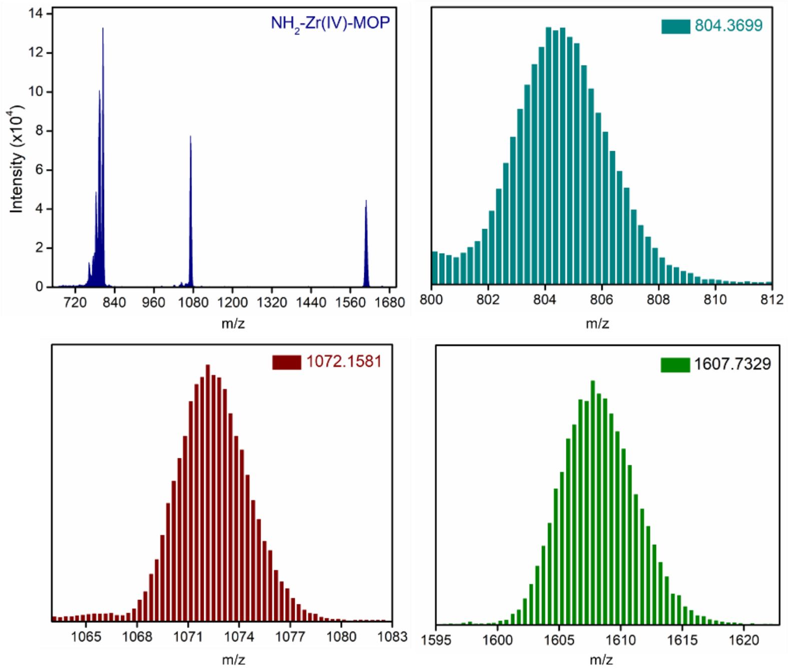

Supplementary Figure 7: Structural characterization of the metal-organic polyhedra (MOPs): ESI-TOF-MS analysis of as-synthesized Zr(IV)-MOP, indicated the formation of the MOPs.

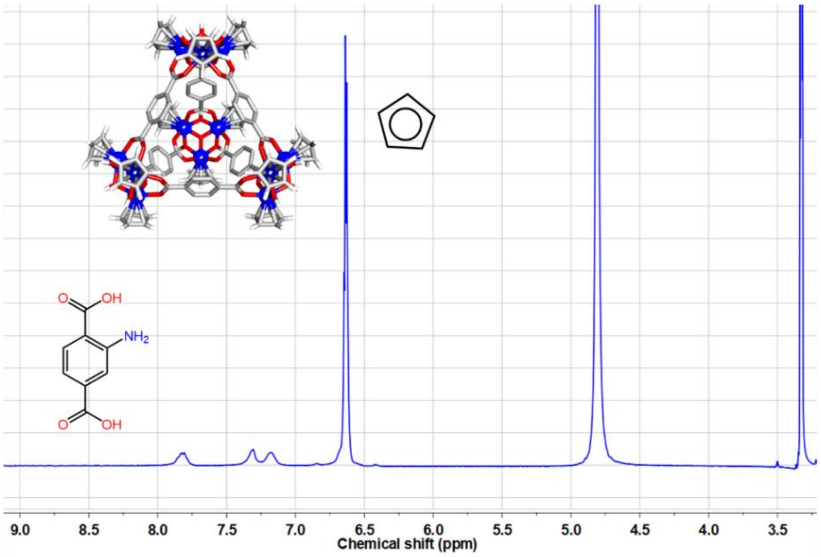

Supplementary Figure 8: ^{1}H NMR spectra of the Zr(IV)-MOPs in MeOD and D_2O.

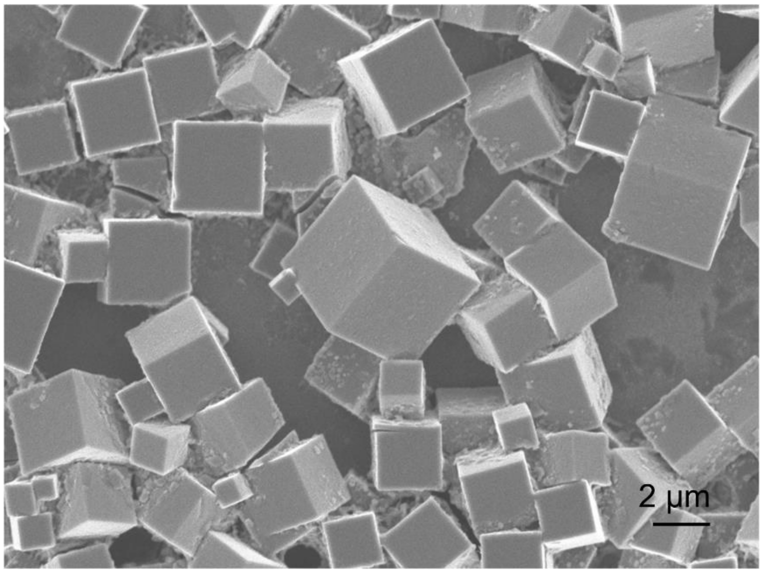

Supplementary Figure 9: FESEM image of the Zr(IV)-MOPs, indicated highly cubic micro-crystalline morphology.

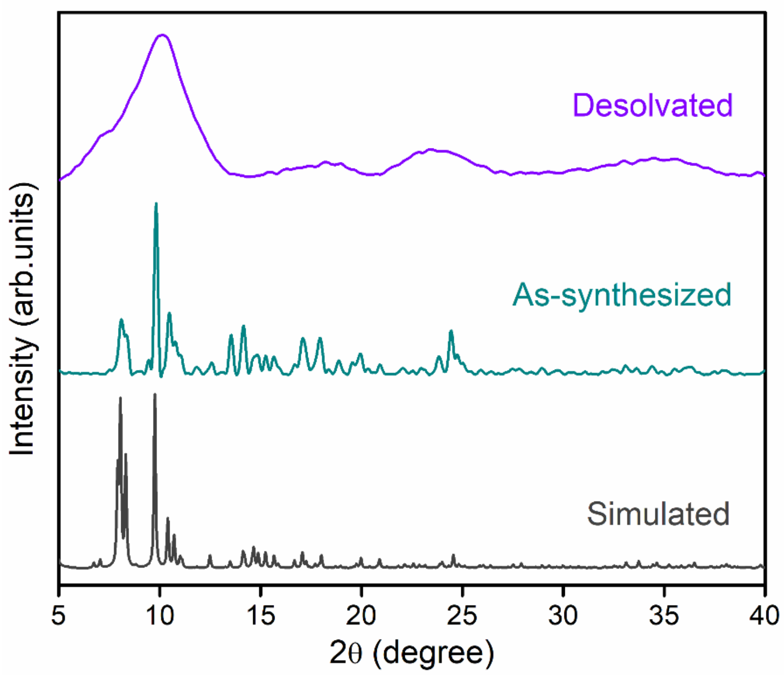

Supplementary Figure 10: PXRD analysis of the Zr(IV)-MOP indicated formation of bulk phase purity of the MOP in as-synthesized phase, also the PXRD data of the desolvated phase showed the weak crystalline nature of the MOPs, which is cause of self-aggregation upon desolvation.

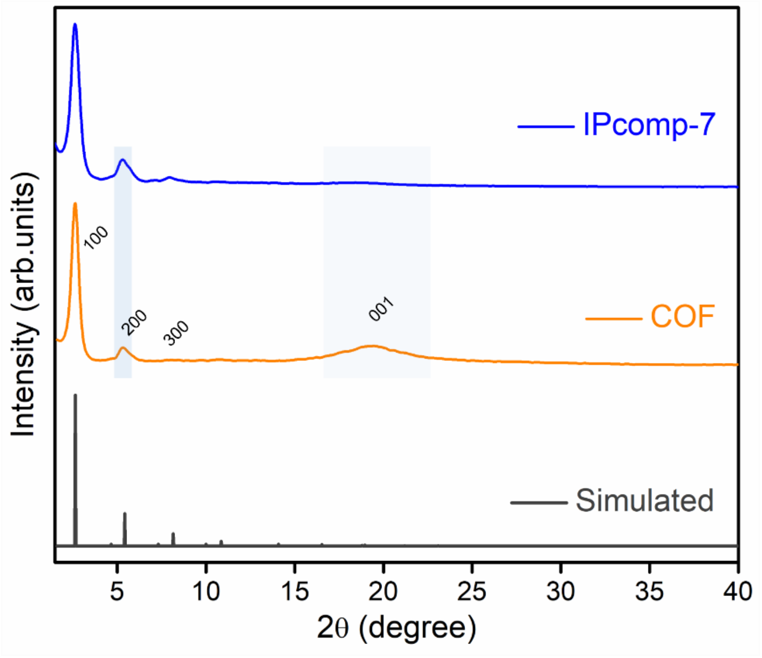

Supplementary Figure 11: PXRD profile of simulated and experimental COF along with IPcomp-7.

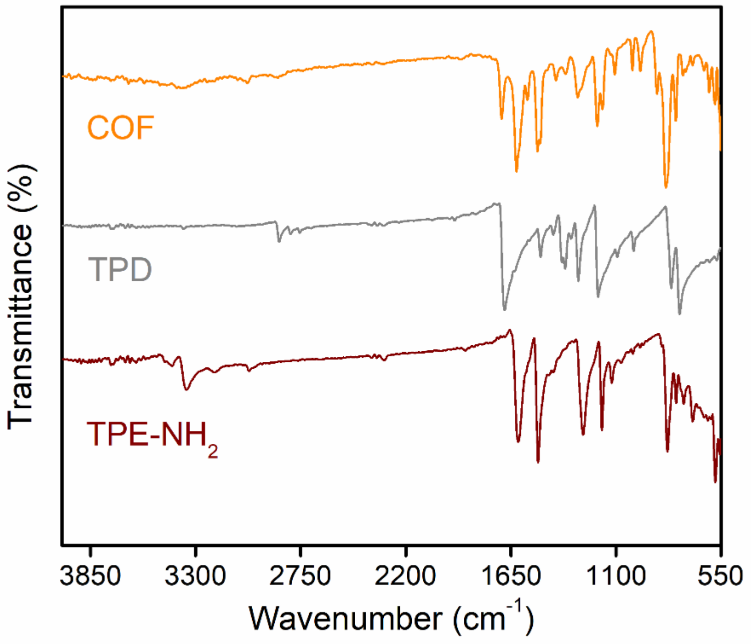

Supplementary Figure 12: FT-IR spectra of the COF aerogel along with its precursors.

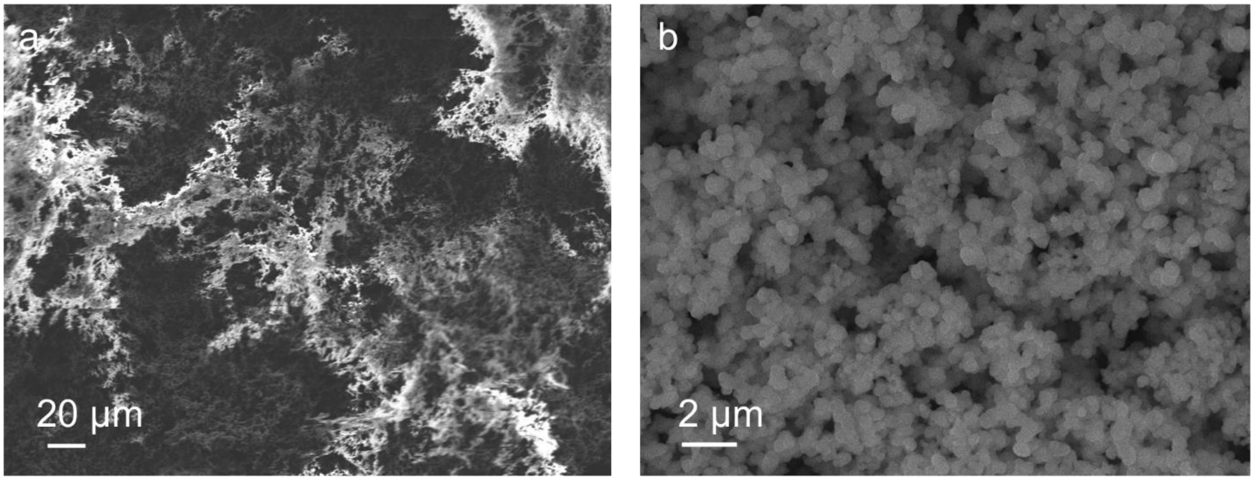

Supplementary Figure 13: (a) SEM and (b) FESEM images of the pristine COF aerogel.

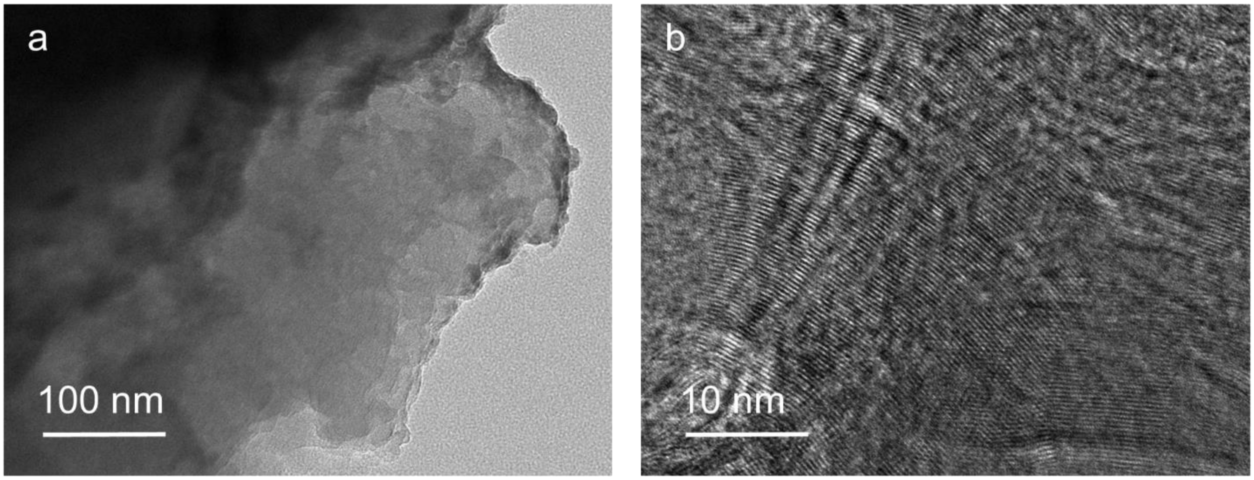

Supplementary Figure 14: (a) TEM and (b) HRTEM images of the pristine COF aerogel.

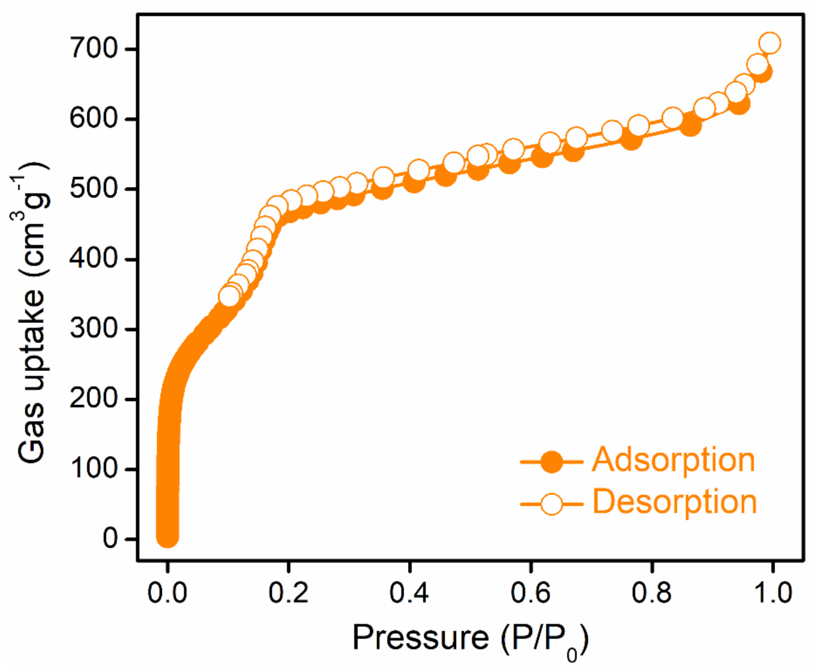

Supplementary Figure 15: Nitrogen gas adsorption-desorption measurement of the COF aerogel at 77K, indicating the existence of hierarchical (micro and meso) porosity.

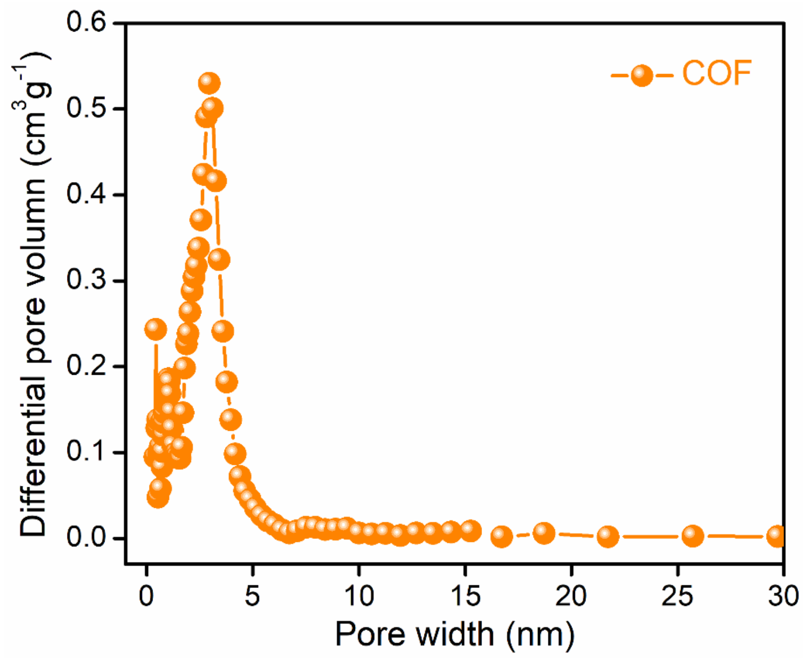

Supplementary Figure 16: NLDFT pore size distribution profile of the dual-pore COF aerogel.

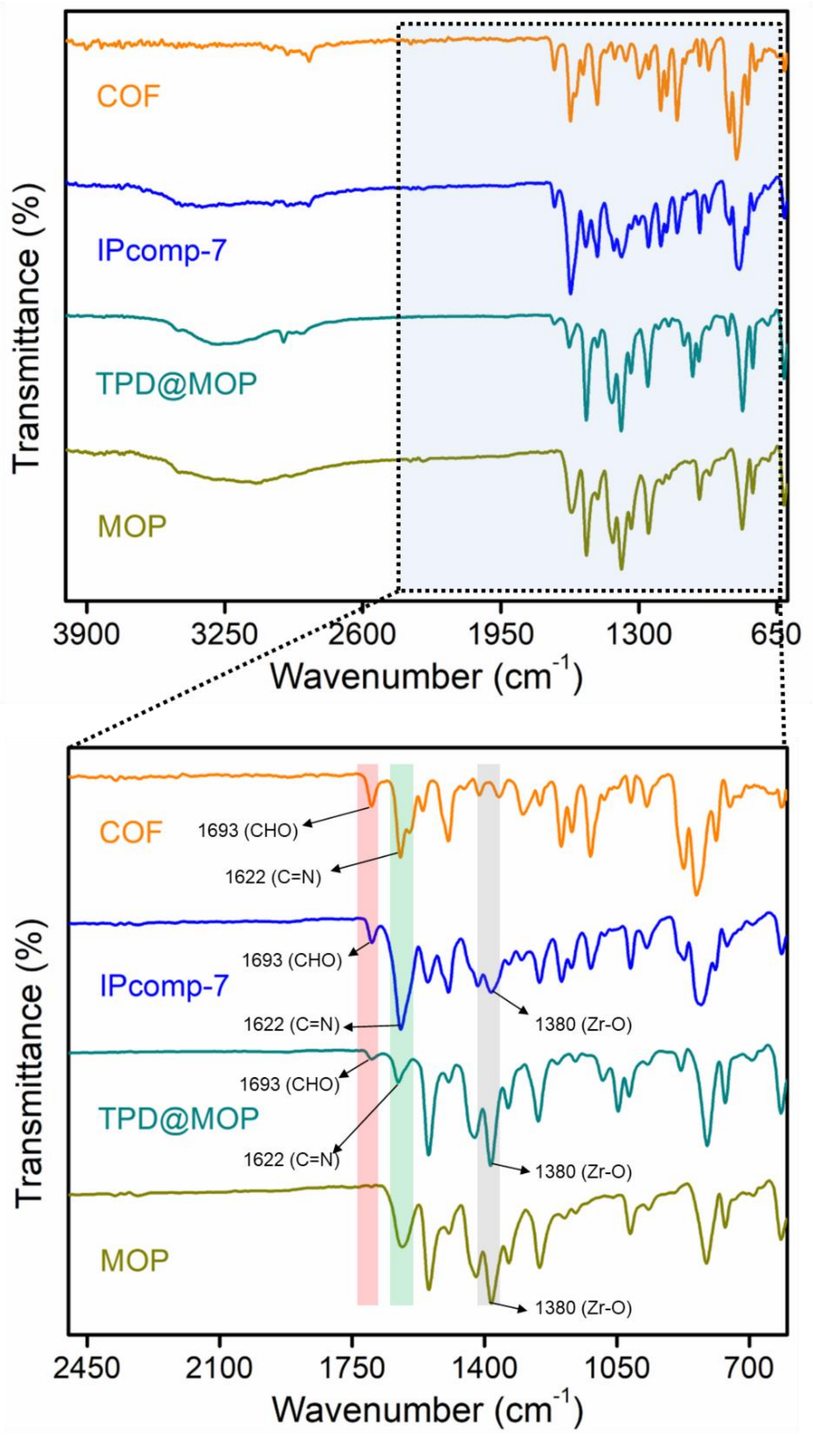

Supplementary Figure 17: FT-IR spectra of IPcomp-7, along with the pristine MOP, TPD@MOP, COF-aerogel.

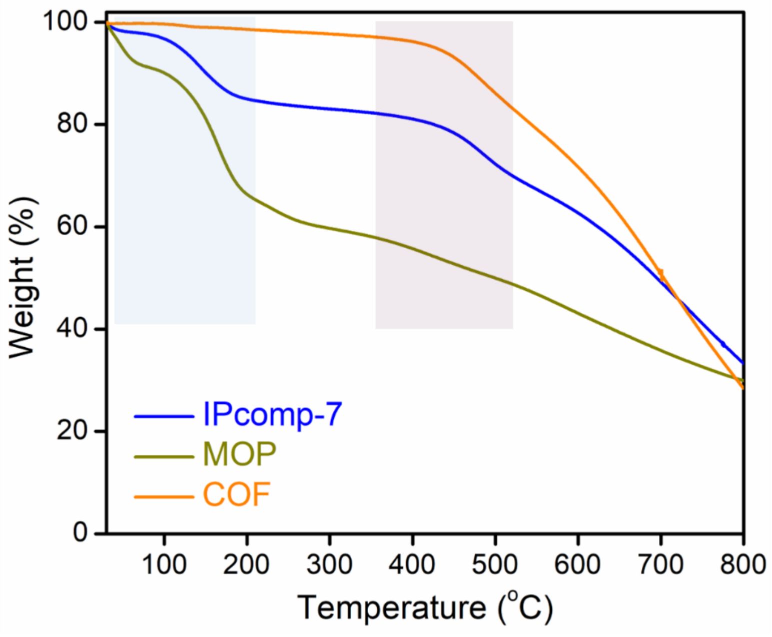

Supplementary Figure 18: TGA profile of IPcomp-7, along with pristine COF-aerogel and MOP.

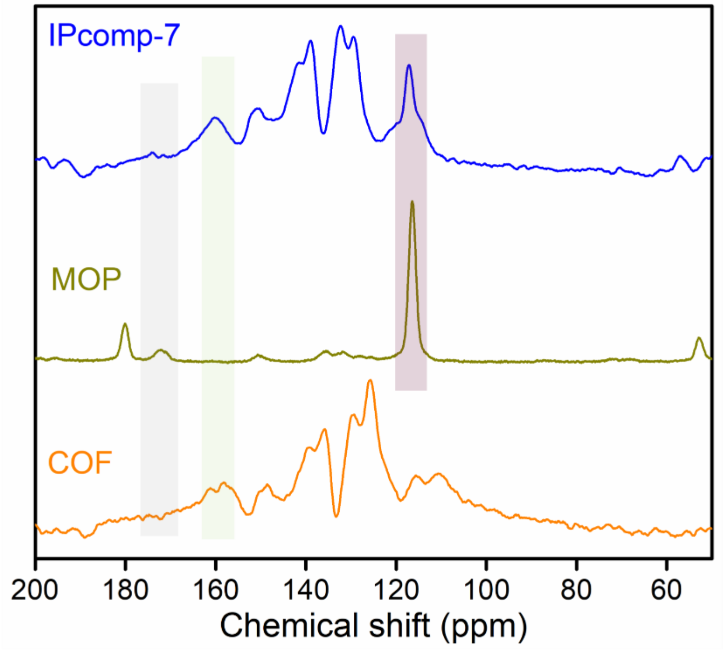

Supplementary Figure 19: Solid-state ^{13}C CP-MAS NMR spectra of IPcomp-7, along with pristine COF-aerogel and MOP.

Supplementary Note 3

In order to further confirm the covalent bonding between the amino functionalized MOP and TPE COF structure, a model compound has been synthesized by reacting the NH_2-MOP with benzaldehyde in the similar reaction condition to that of hybrid composite (IPcomp-7) synthesis. The following ^{1}H NMR data displayed the reaction of NH_2-MOP with aldehyde as indicated by the appearance of new peaks in the chemical shift range of ~7.4-7.7 ppm (Supplementary Figure 20).[1]

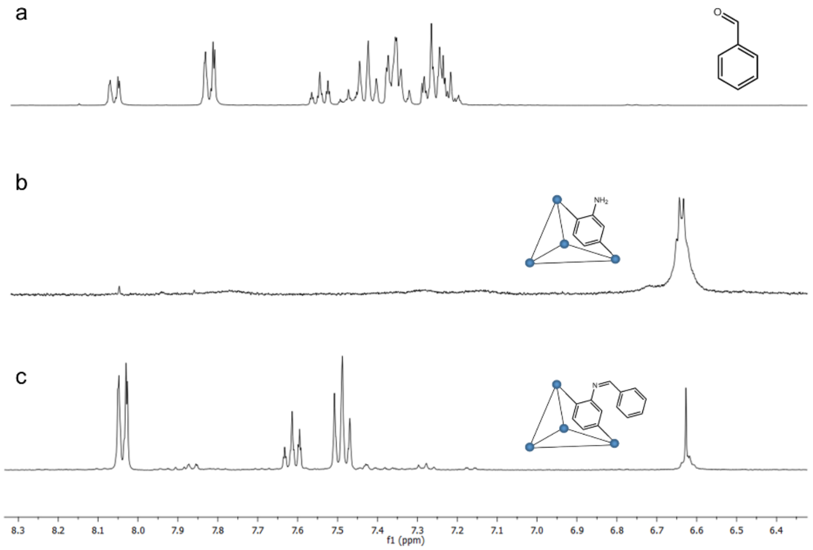

Supplementary Figure 20: ^{1}H NMR spectra of (a) benzaldehyde, (b) MOP and (c) benzaldehyde treated MOP with solvents.

The analysis of the aqueous supernatant collected from the unwashed pristine composite using ^{1}H NMR, HRMS, and ICP data revealed the presence of MOP molecules (Supplementary Figure 21). This finding can be attributed to the simple liberation of non-covalently bonded MOP molecules from the surface of IPcomp-7. However, the analysis of IPcomp-7 treated supernatant, showed no presence of MOPs after through washing with aqueous methanolic solution, arguing out the occurrence of excess MOPs on the surface of the composite and the absence of MOP liberation from the COF aerogel matrix (Supplementary Figure 22). Additionally, the appearance of Zr metal in the ICP data of digested sample of IPcomp-7 and the relevant peaks of the corresponding organic linkers of MOP in the ^{1}H NMR spectra confirmed the existence of covalently connected amino-Zr(IV)-MOPs in the hierarchical porous COF aerogel matrix (Supplementary Figure 23).

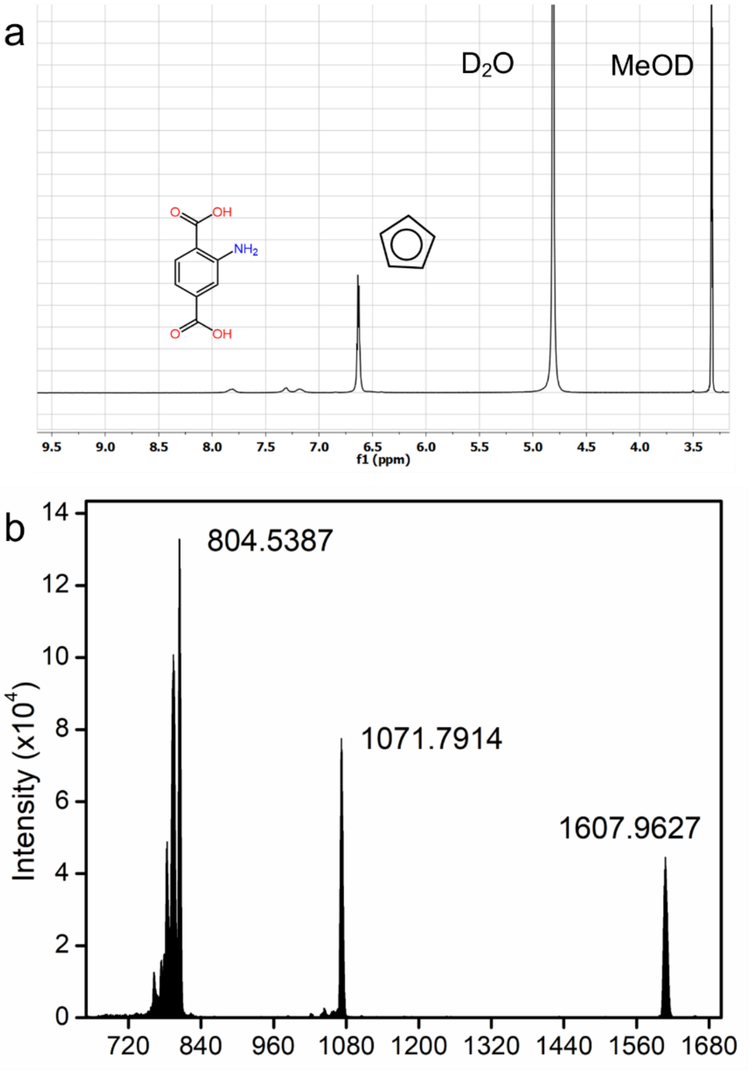

c

Samples	Zr (ppm)
Sample-1	5.89
Sample-2	2.17
Sample-3	8.22

Supplementary Figure 21: Characterizations data of IPcomp-7 treated supernatant, indicating the presence of guest-MOP molecules before washing IPcomp-7 with aqueous methanolic solution: (a) ^{1}H NMR spectra of the MOP in MeOD, D_2O mixture, (b) HRMS spectra of the MOP, (c) ICP analysis of Zr(IV) metal in the supernatant of hybrid material exposed to aqueous methanolic solution.

a

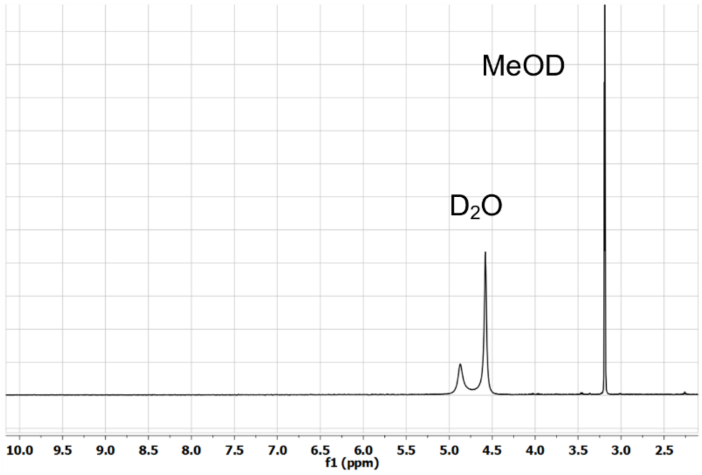

b

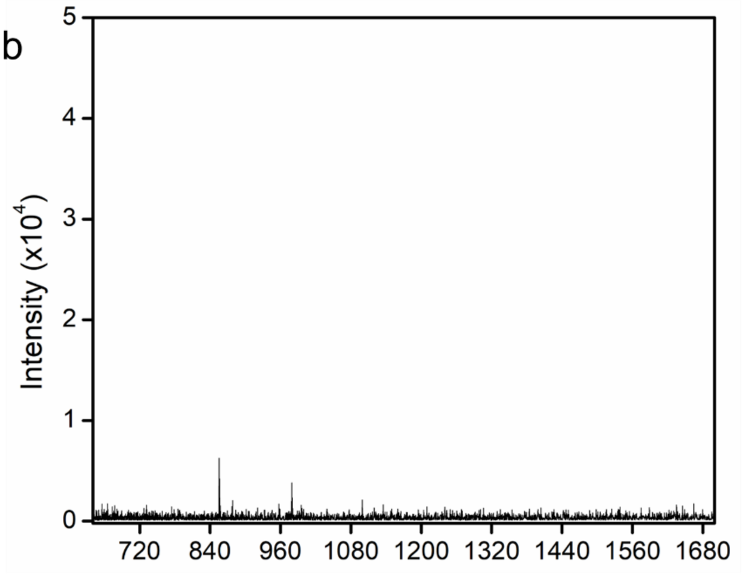

c

**Samples**	**Zr (ppm)**
Sample-1	NA
Sample-2	0.11
Sample-3	0.04

Supplementary Figure 22: Characterizations data of IPcomp-7 treated supernatant, indicating no presence of guest-MOP molecules after washing IPcomp-7 with aqueous methanolic solution: (a) ^{1}H NMR spectra, (b) HRMS spectra, (c) ICP analysis of Zr(IV) metal in the supernatant of hybrid material exposed to aqueous methanolic solution.

a

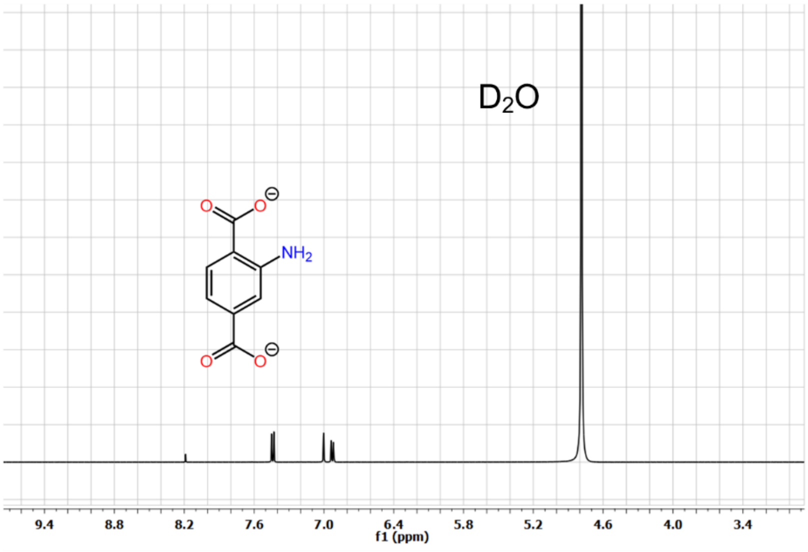

b

Samples	Zr (ppm)
Sample-1	0.97
Sample-2	3.84
Sample-3	1.06

Supplementary Figure 23: Characterizations of the digested sample of IPcomp-7: (a) ^{1}H NMR spectra of the organic linker of MOP in NaOD/D_2O solvent. (b) ICP analysis of Zr(IV) metal in the supernatant of the digested hybrid material.

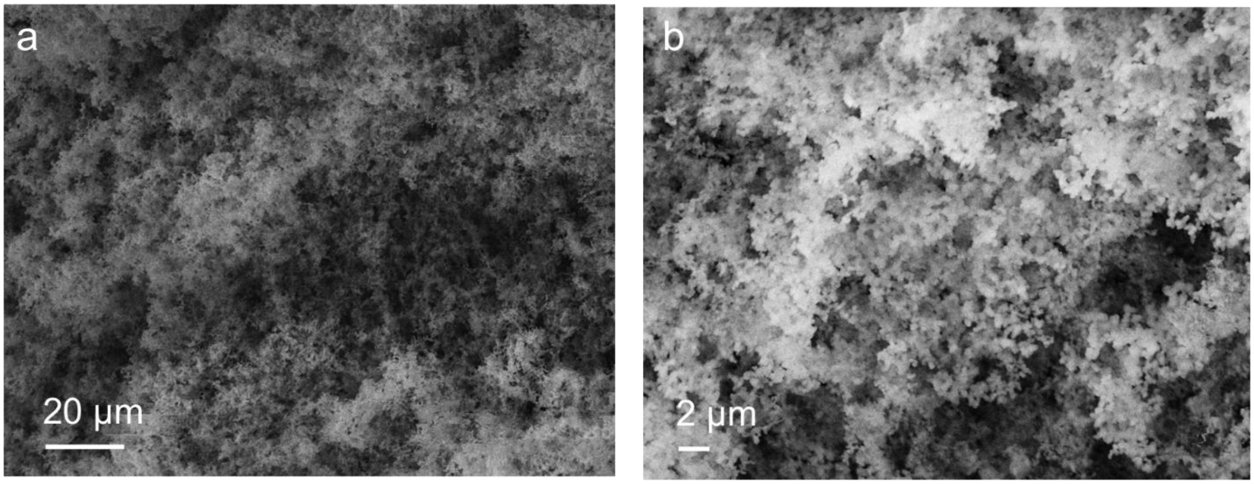

Supplementary Figure 24: FESEM images of the hybrid aerogel (IPcomp-7) (a) 20 µm, (b) 2 µm. (The numerical values represent the scale bars of the images).

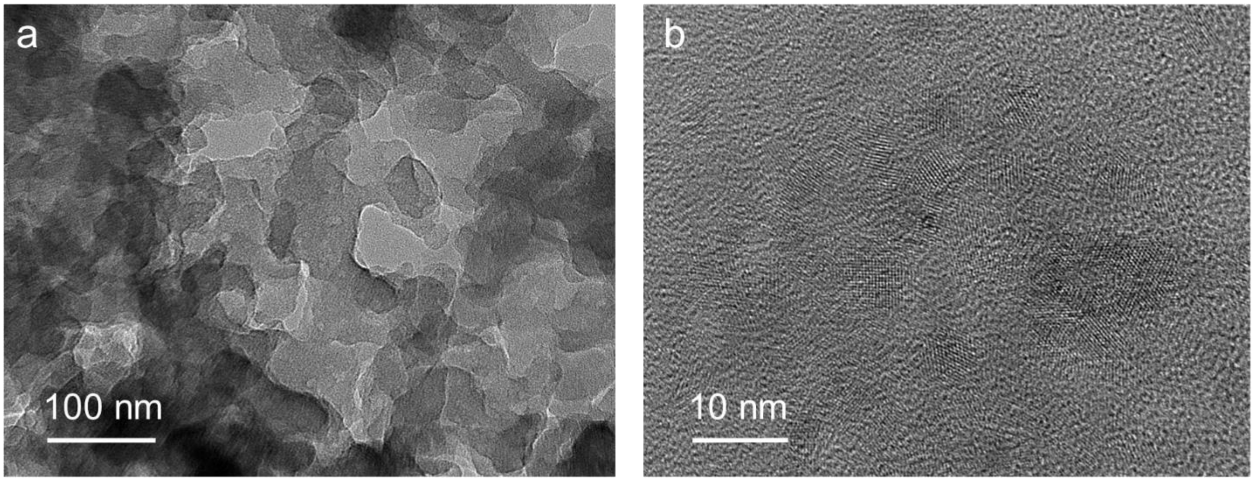

Supplementary Figure 25: TEM/HRTEM images of the hybrid composite (IPcomp-7) (a) 100 nm, (b) 10 nm. (The numerical values represent the scale bars of the images).

Supplementary Table 1: EDX elemental analyses from FESEM experiment of NH_2-Zr(IV)-MOP molecules showing presence of C, N, O, Cl and Zr elements (a).

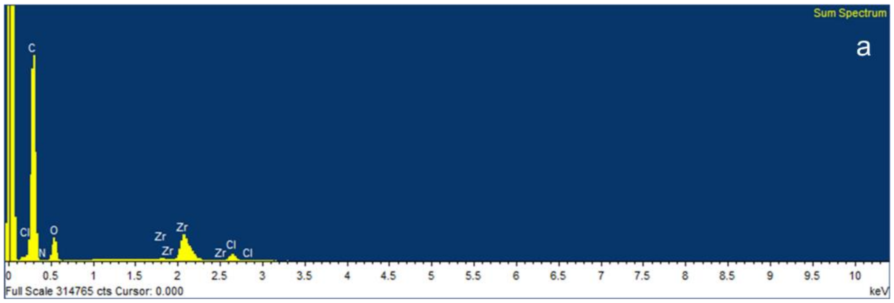

Element	Weight%	Atomic%
C K	71.95	82.52
N K	2.70	2.63
O K	15.37	13.09
Cl K	1.18	0.45
Zr L	8.80	1.31
Totals	100.00	

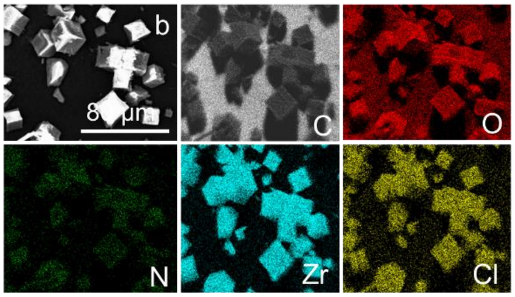

Supplementary Figure 26: (b) SEM elemental mapping images (scale bar 80 µm) of the NH_2-Zr(IV)-MOP molecules.

Supplementary Table 2: EDX elemental analyses from FESEM experiment of the COF-aerogel showing presence of C and N elements (a)

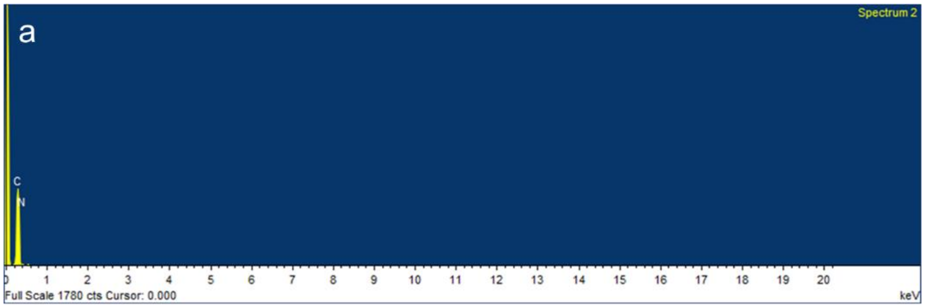

Element	Weight%	Atomic%
C K	90.54	91.77
N K	9.46	8.23
Totals	100.00	

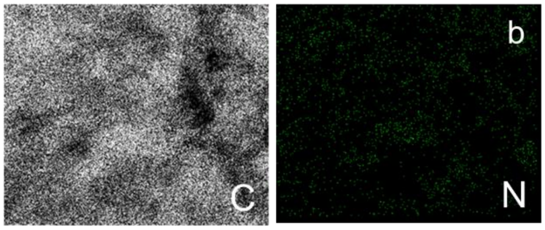

Supplementary Figure 27: (b) SEM elemental mapping images of the COF-aerogel.

Supplementary Table 3: EDX elemental analyses from (a) FESEM and (b) TEM experiment of IPcomp-7, constructed with ~15 mg of MOPs showing the presence of C, N, O, Al, Cl and Zr elements.

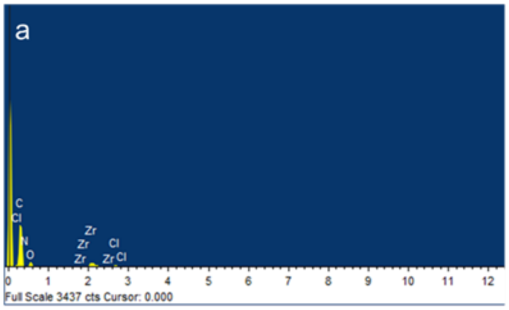

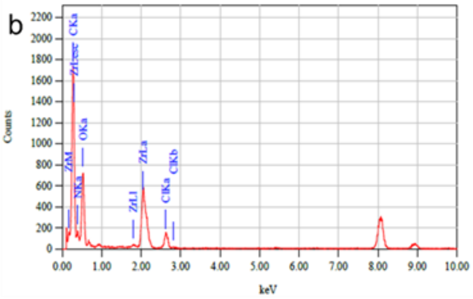

Element	Weight%	Atomic%
C K	72.11	79.79
N K	5.52	4.00
O K	19.77	15.61
Cl K	0.44	0.16
Zr L	2.16	0.44
Totals	100.00	

Element	Weight%	Atomic%
C K	74.13	77.02
N K	3.63	4.27
O K	12.79	13.17
Cl K	2.04	0.95
Zr L	7.41	4.59
Totals	100.00	

Supplementary Table 4: EDX elemental analyses from FESEM experiment of the hybrid composite, constructed with ~35 mg of MOPs showing presence of C, N, O, Al, Cl and Zr elements.

Element	Weight%	Atomic%
C K	75.62	81.99
N K	6.29	5.66
O K	13.87	11.22
Cl K	0.74	0.27
Zr L	3.48	0.86
Totals	100.00	

Supplementary Table 5: EDX elemental analyses from FESEM experiment of the hybrid composite, constructed with ~50 mg of MOPs showing presence of C, N, O, Al, Cl and Zr elements

Element	Weight%	Atomic%
C K	76.92	85.84
N K	-0.77	-0.74
O K	15.42	12.98
Cl K	1.13	0.43
Zr L	7.30	1.49
Totals	100.00	

Supplementary Table 6: EDX elemental analyses from FESEM experiment of the hybrid composite, constructed with ~100 mg of MOPs showing presence of C, N, O, Al, Cl and Zr elements.

Element	Weight%	Atomic%
C K	74.77	79.33
N K	7.58	5.31
O K	11.66	10.56
Cl K	1.08	1.16
Zr L	4.91	3.64
Totals	100.00	

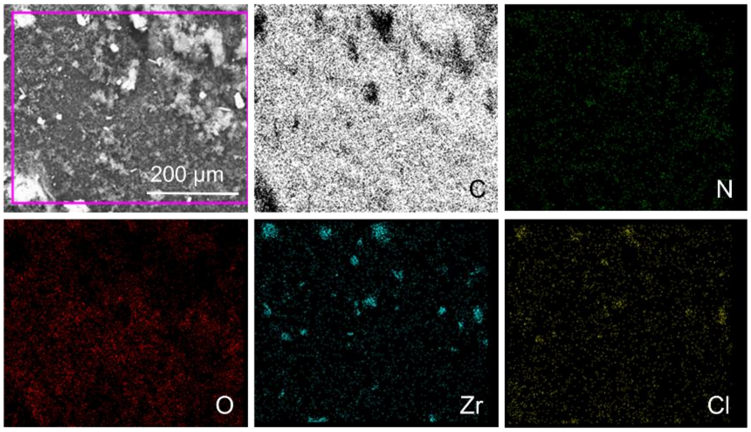

Supplementary Figure 28: SEM elemental mapping images (scale bar 200 µm) of IPcomp-7 indicated homogeneous distribution of all relevant elements (Carbon (C), Nitrogen (N), Oxygen (O), Zirconium (Zr) and Chlorine (Cl)).

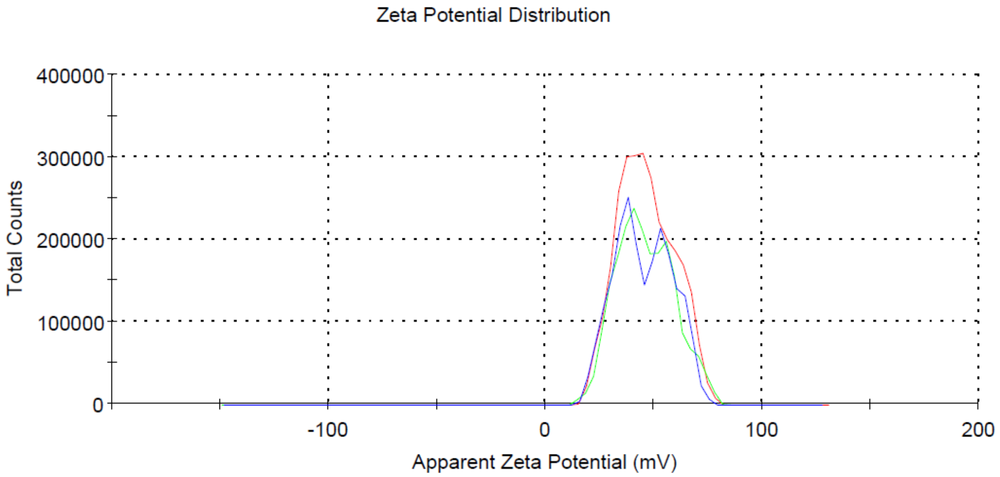

Supplementary Figure 29: Zeta potential measurement of IPcomp-7 indicated cationic nature of the composite with potential value of +46.3 mV.

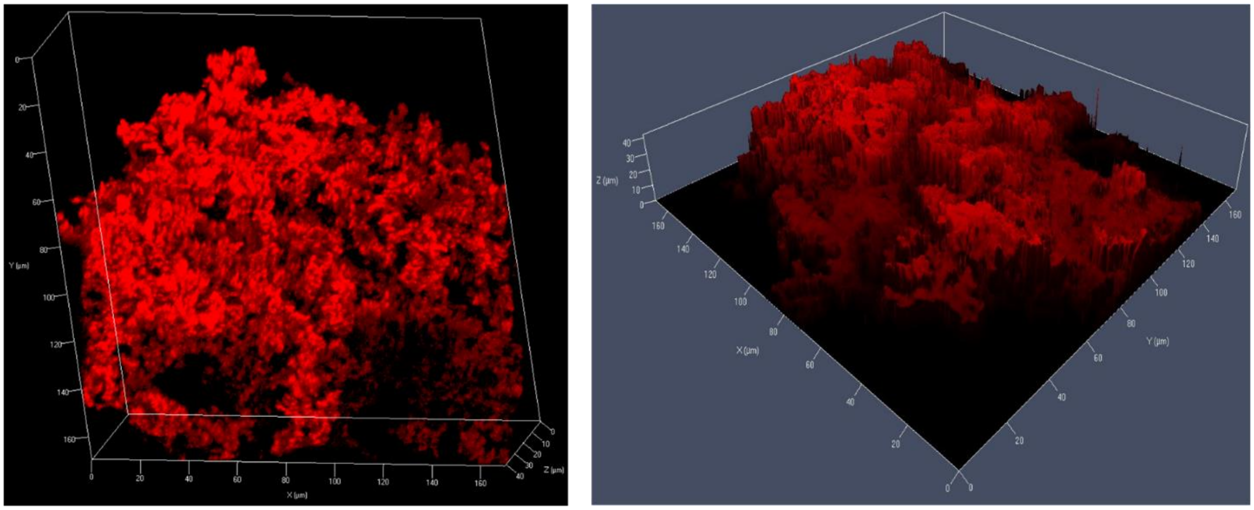

Supplementary Figure 30: Confocal fluorescence 3D images of IPcomp-7 indicated the distribution of extrinsic macropores throughout the surface of the composite.

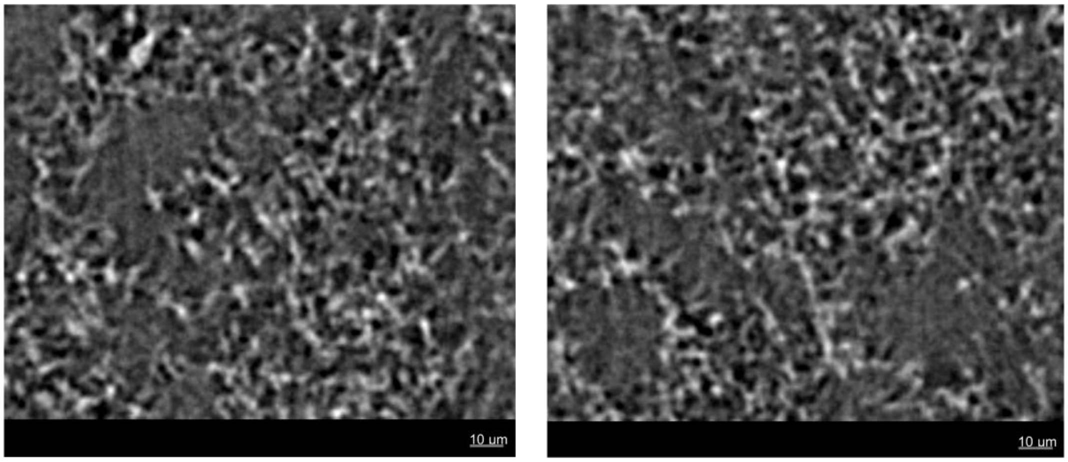

Supplementary Figure 31: Two-dimensional cross-sectional tomography images of IPcomp-7 indicated distribution of large macropores throughout the composite matrix.

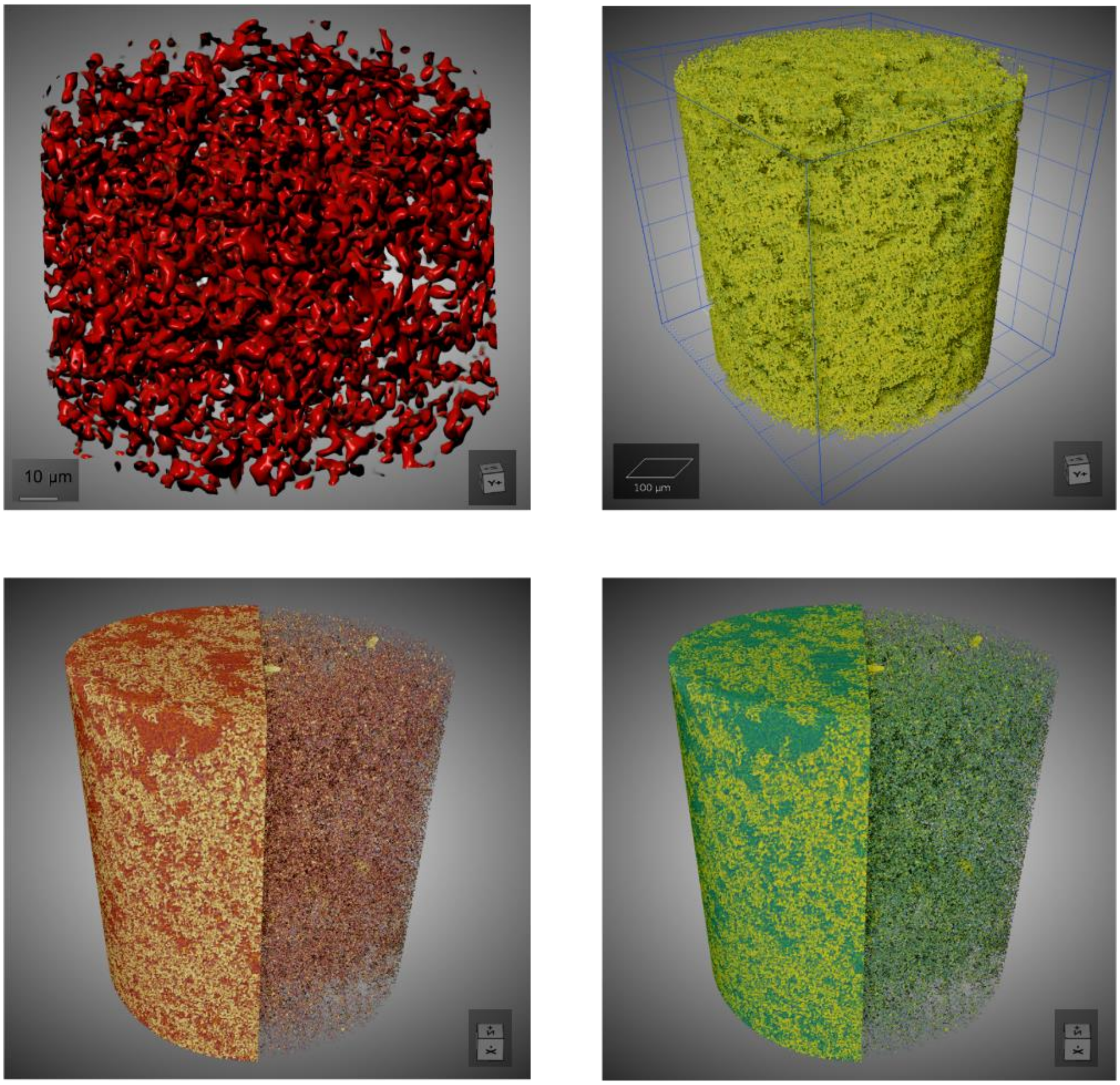

Supplementary Figure 32: Computed X-ray tomographic segmented 3D models of IPcomp-7 showing the distribution of large open voids (macropores) throughout the structure of the composite.

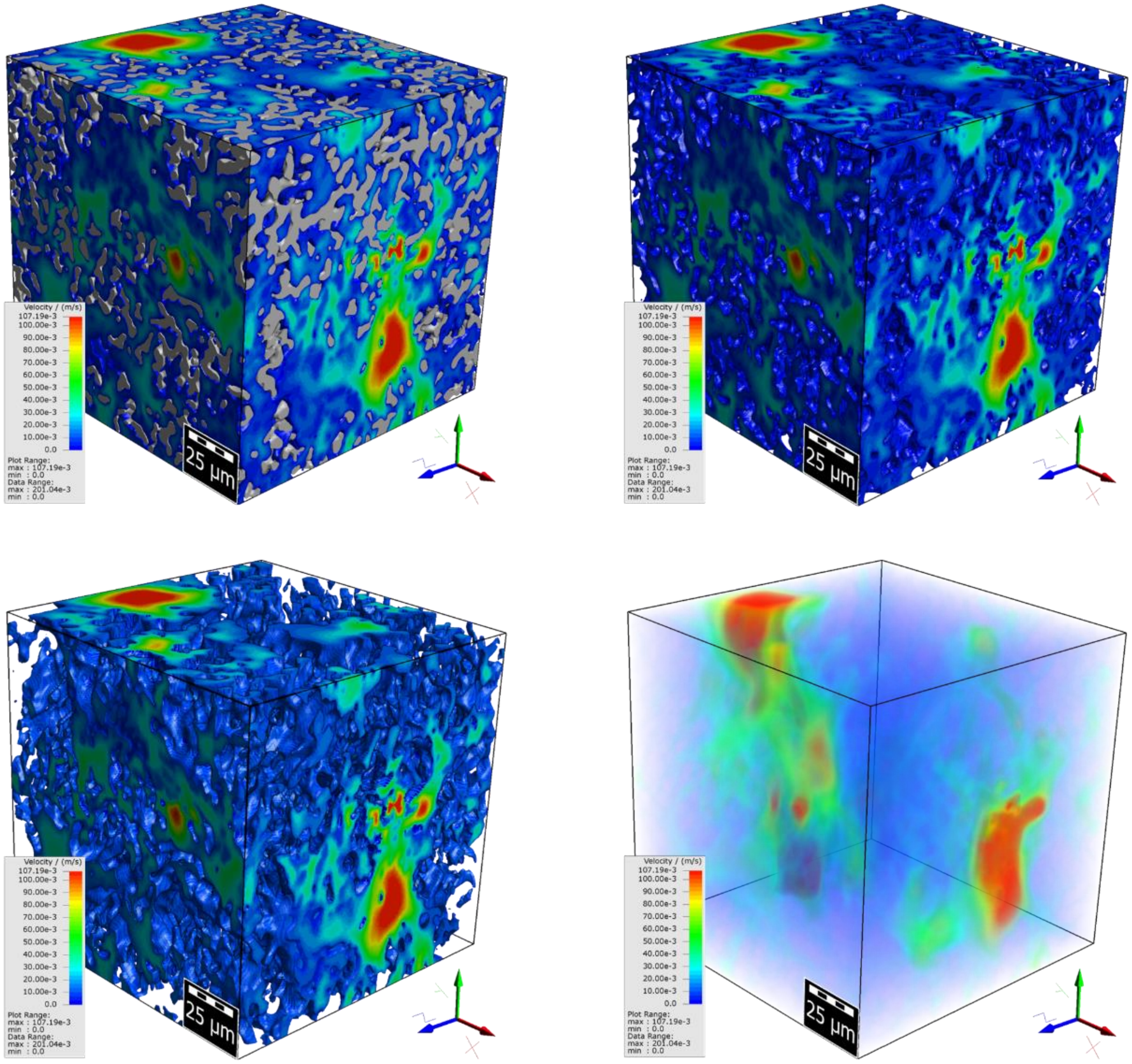

Supplementary Figure 33: Color-coded 3D CT images of IPcomp-7 demonstrating the visualization of pore size distribution of the large macropores throughout the structure.

Supplementary Note 4

The optical characteristics of IPcomp-7 were further utilized to demonstrate the successful grafting of amino-functionalized cationic MOPs in the structure of the COF aerogel. The UV-vis diffuse reflectance spectroscopy (DRS) spectra exhibited typical adsorption bands for MOP and COF-aerogel, demonstrating the presence of both constituents in the hybrid nanocomposite (Supplementary Figure 34). The solid-state photoluminescence spectra of IPcomp-7 yielded identical observation with a noticeable blue shift in the characteristic peak due to the entrapment of discrete MOP molecules inside the COF aerogel matrix (Supplementary Figure 35).[2, 3] In addition, once the amino group containing MOP molecules were successfully grafted, the weakly emissive COF aerogel was transformed into a highly emissive material, which is further emphasized by fluorescence microscopic imaging (Supplementary Figure 36). Moreover, the compression measurements were performed to study the mechanical properties of the hybrid aerogels and produce stress-strain curves (Supplementary Figure 37).

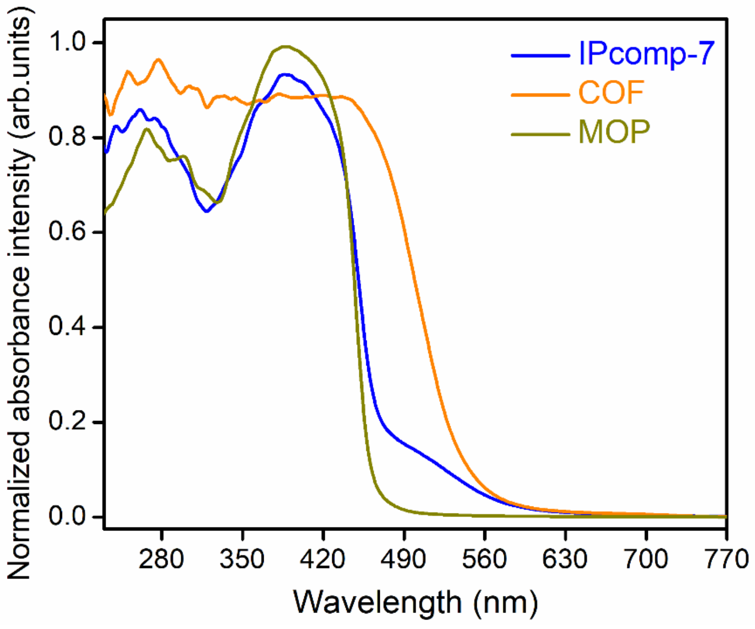

Supplementary Figure 34: Solid-state UV-vis spectra of IPcomp-7, along with pristine MOP and COF-aerogel.

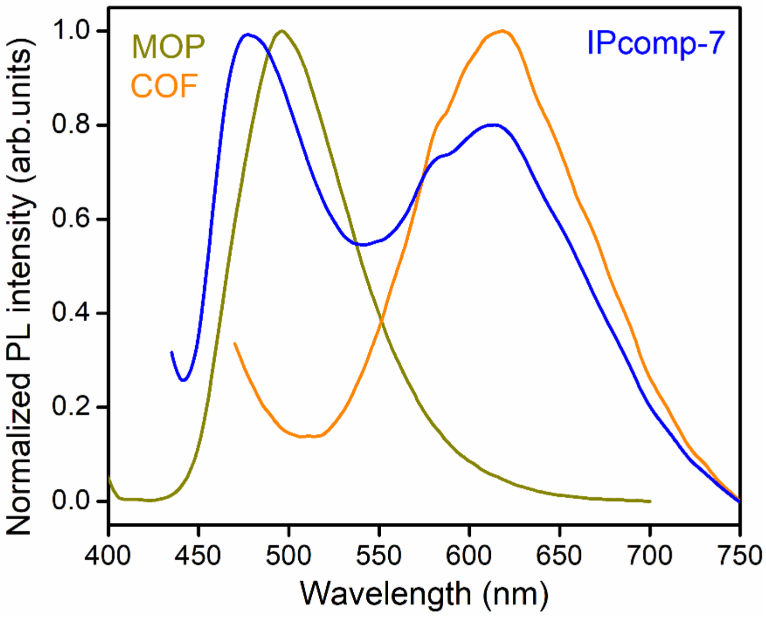

Supplementary Figure 35: Solid-state PL spectra of IPcomp-7, along with pristine MOP and COF-aerogel.

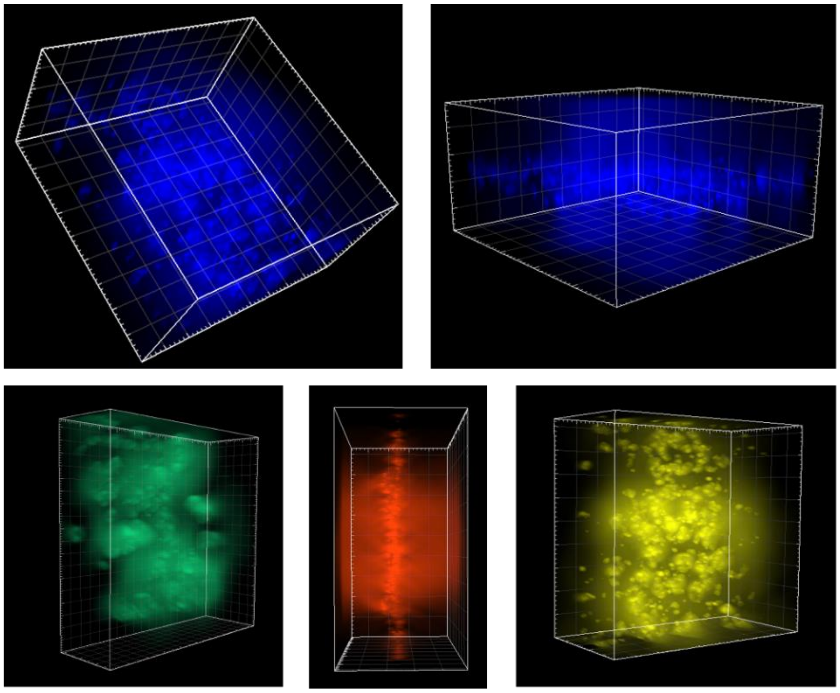

Supplementary Figure 36: 3D fluorescence images of IPcomp-7 showing the highly emissive nature of the composite.

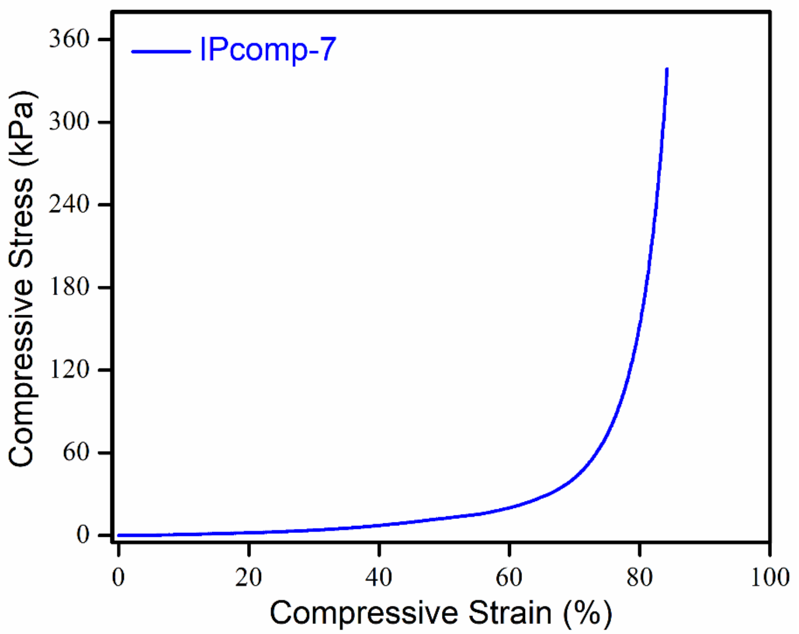

Supplementary Figure 37: Compressive stress-strain curves of the hybrid aerogel show promising mechanical stability**.**

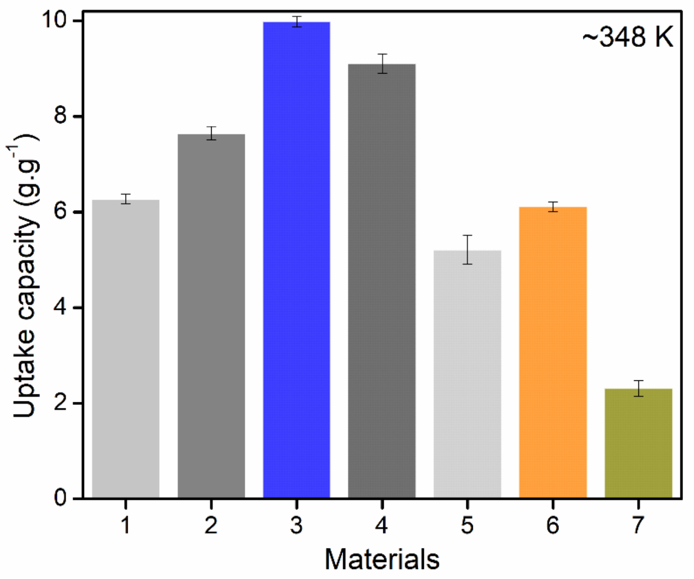

Supplementary Figure 38: An optimization study of iodine capture, using different amounts of guest-MOP molecules in the hybrid composite exhibiting, IPcomp-7 constructed by ~15 mg MOPs showed highest sequestration capacity. (1 = 1 mg, 2 = 5 mg, 3 = 15 mg (IPcomp-7), 4 = 25 mg, 5 = 50 mg MOPs used in the synthesis of the composite, 6 = COF, and 7 = MOP) [Condition: adsorbent amount = 10 mg, contact time = 24 hours]. (The error bars are the standard deviations from three parallel measurements).

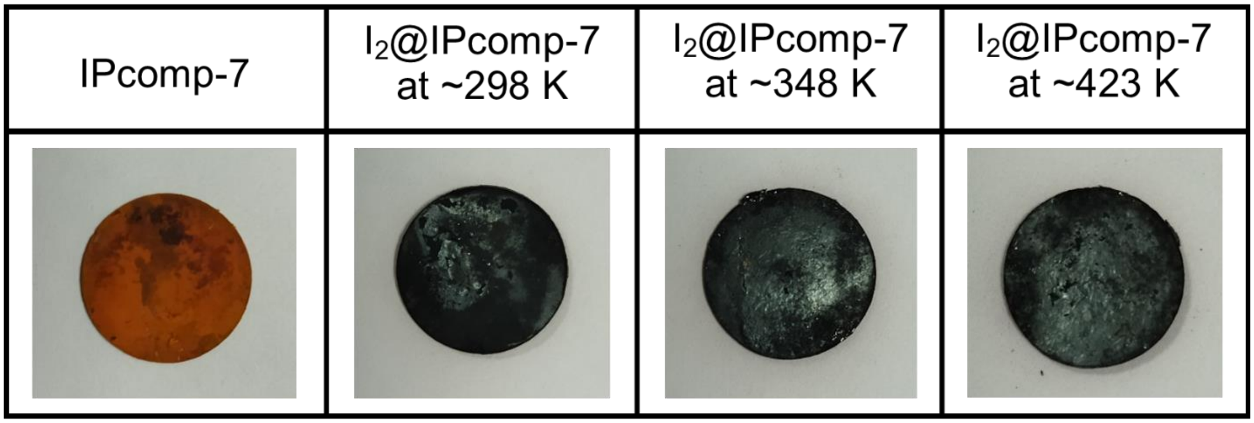

Supplementary Figure 39: Digital images of IPcomp-7 before and after exposing to iodine at different temperatures for 24 hours.

Supplementary Table 7: Comparison table of vapor phase iodine adsorption capacities of various adsorbents in static condition.

Materials	Temp. (°C)	Capacity ($g.g^{-1}$)	Material type	Ref.
IPcomp-7	75	9.98	MOP/COF composite	This work
COF-OH-33	75	6.81	COF	4
COF-OH-50	75	6.49	COF	
iCOF-AB-33	75	9.00	COF	
iCOF-AB-50	75	10.21	COF	
ETTA-TPA	75	4.79	COF	5
TPB-DMTP	75	6.26	COF	
TTA-TTB	75	4.95	COF	
Meso-COF-2	75	3.5	COF	6
Meso-COF-3	75	4.0	COF	
COF-300	75	3.5	COF	
COF-320	75	4.0	COF	
COF-DL229	75	4.7	COF	7
SCU-COF-2	75	6.0	COF	8
JUC-560	75	5.20	COF	9
JUC-561	75	8.19	COF	
QTD-COF-V	75	6.29	COF	10
COF-LZU1	77	5.30	COF	11
TAPA-PDA COF	77	5.09	COF	12
TJNU-201	77	5.625	COF	13
TFB-DB-COF	75	6.4	COF	14
COF-SIO-COF-7	75	4.81	COF	15
HcOFs	75	4.73	HOF	16
C[4]P-BTP	75	3.38	POP	17
CMP-4	75	2.08	POP	18
CalP2-Li	75	1.08	POP	19
CalP3-Li	75	2.48	POP	
CalP4-Li	75	3.12	POP	
CalPOF-1	75	4.77	POP	20
CalPOF-3	75	3.53	POP	
PAF-23	75	2.71	POP	21
PAF-24	75	2.76	POP	
PAF-25	75	2.60	POP	
COP_1^0	60	3.80	POP	22
CMP-LS4	70	3.32	POP	23
CMP-LS5	70	4.4	POP	
Azo-PPN	77	2.90	POP	24
Azo-Trip	77	2.38	POP	25
NiMoS	60	2.25	Chalcogels	26

CMPN	70	2.08	POP	27
NiP-CMP	77	2.02	POP	28
NTP	75	1.80	POP	29
BisImi-POP@2	77	10.30	POP	30
TBIM	77	9.43	POP	31
PSIF-5a	75	5.75	POP	32
CMPN	75	5.03	POP	33
CSU-CPOPs-1	75	4.95	POP	34
ZIF-8	75	1.20	MOF	35
HKUST-1	75	0.64	MOF	36
MOF-808	80	2.18	MOF	37
PCN-333(Al)	75	4.42	MOF	38
HKUST-1@PES	75	5.38	MOF	39
Ag^{+}@Zeolite	95	0.28	Zeolite	40

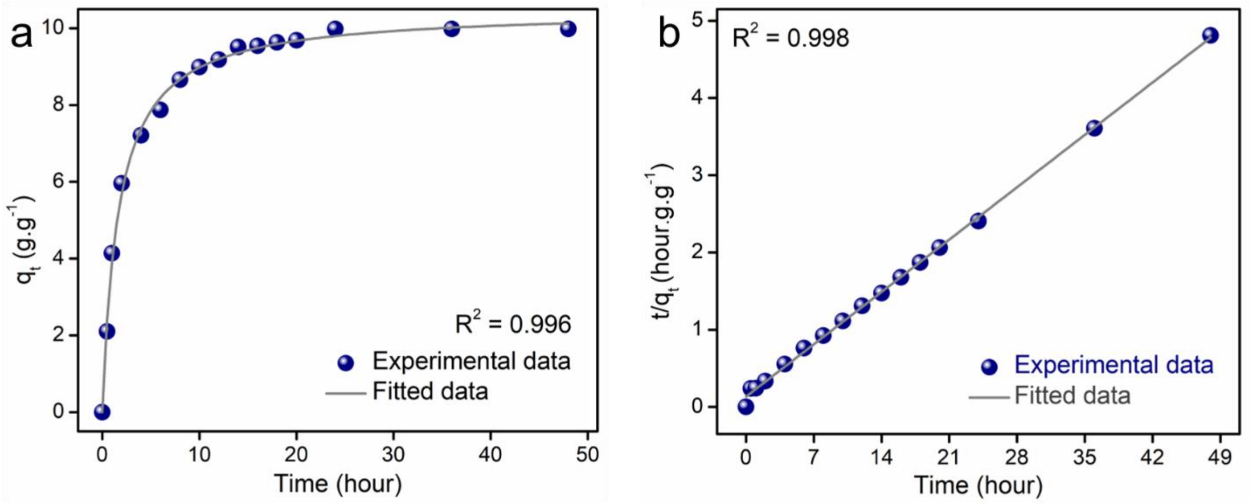

Kinetic parameters for the vapor phase static iodine adsorption by IPcomp-7

q_e (exp) = 9.98 g g^{-1}

Pseudo-second-order model

$q_t = (q_e^2 k_2 t)/(1+q_e k_2 t)$

q_e (g g^{-1})	k_2 (g g^{-1} hour^{-1})	R^2
10.484	0.0567	0.996

Supplementary Figure 40: Pseudo-second-order model (a) non-linear and (b) linear fitting and kinetics parameters (from non-linear equation) for vapor phase static iodine capture study by IPcomp-7 at 75 ^oC.

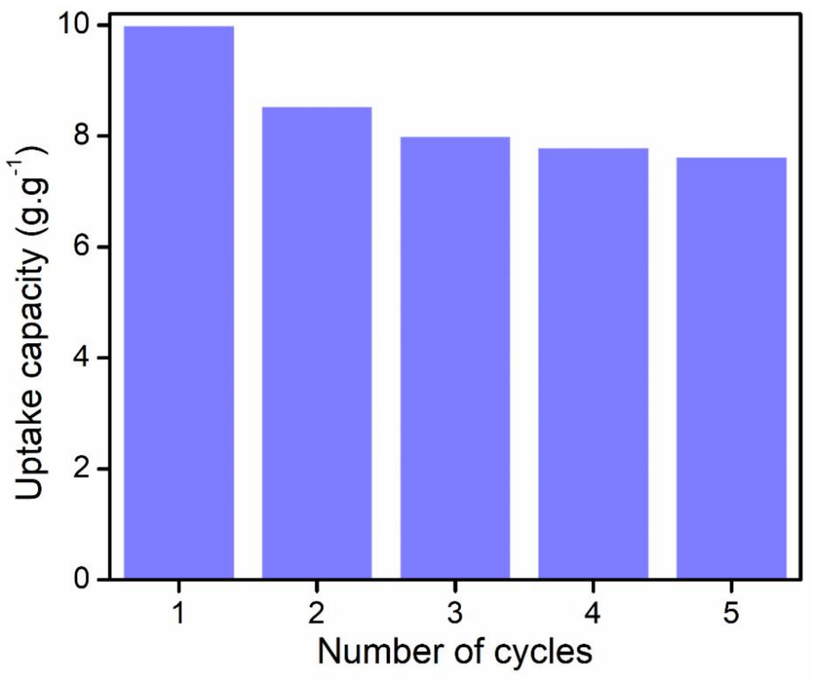

Supplementary Figure 41: The recyclability result of IPcomp-7 towards vapor phase iodine capture in static system.

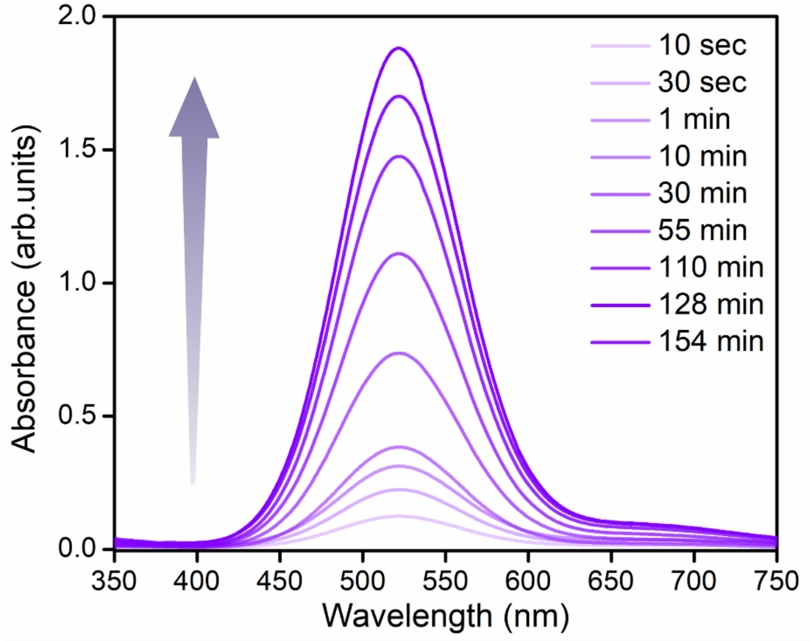

Supplementary Figure 42: Time-dependent UV-vis spectra of iodine released from I_2@IPcomp-7 in n-hexane solution.

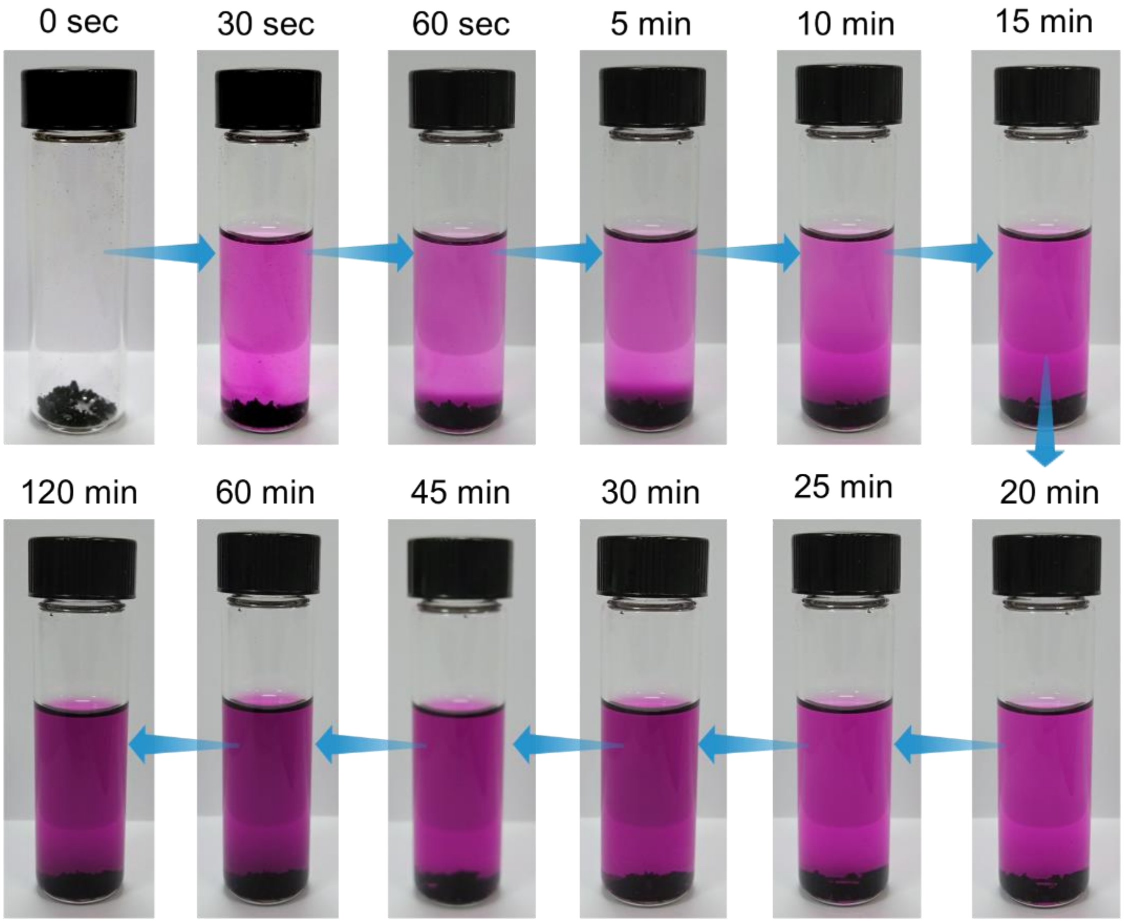

Supplementary Figure 43: Time-dependent images of the iodine released process from I_2@IPcomp-7 in n-hexane solution as the treatment progressed.

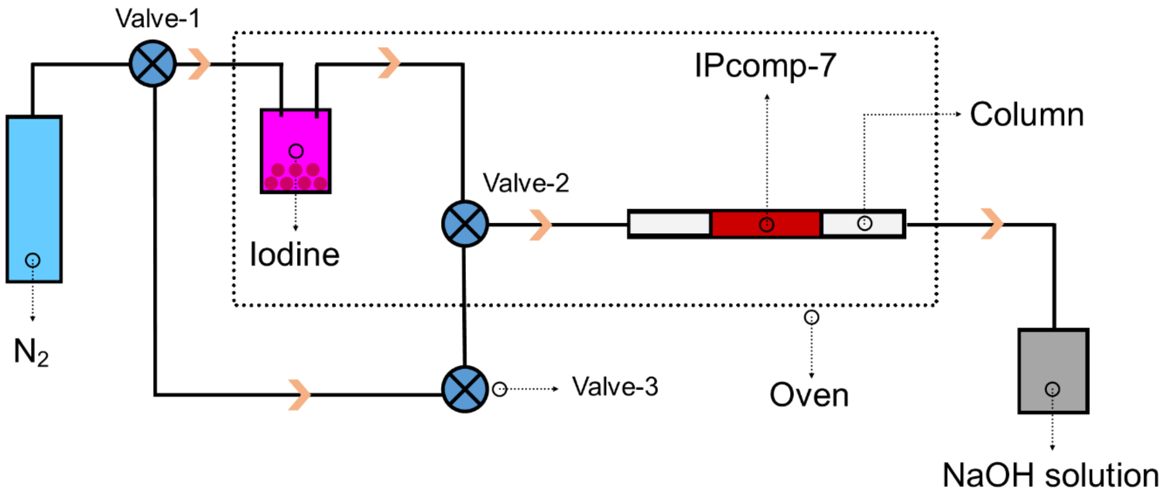

Supplementary Figure 44: Schematic representation of the instrument setup for dynamic gaseous iodine adsorption experiment at high temperature.

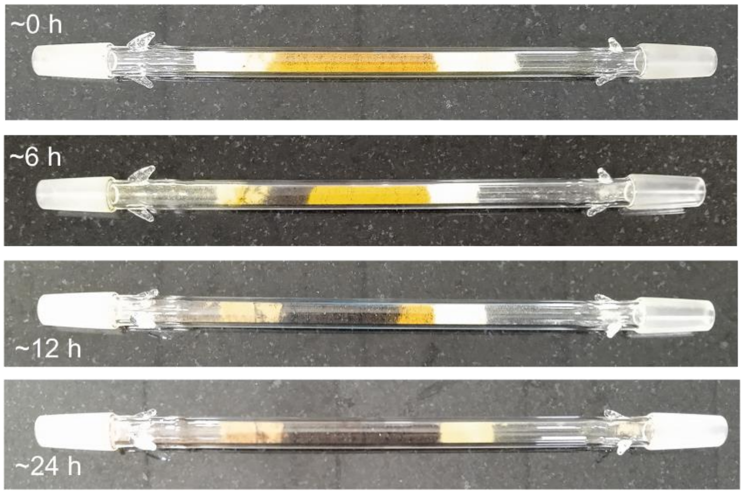

Supplementary Figure 45: Digital images of IPcomp-7 packed glass columns before and after dynamic gaseous iodine adsorption experiment at 348 K.

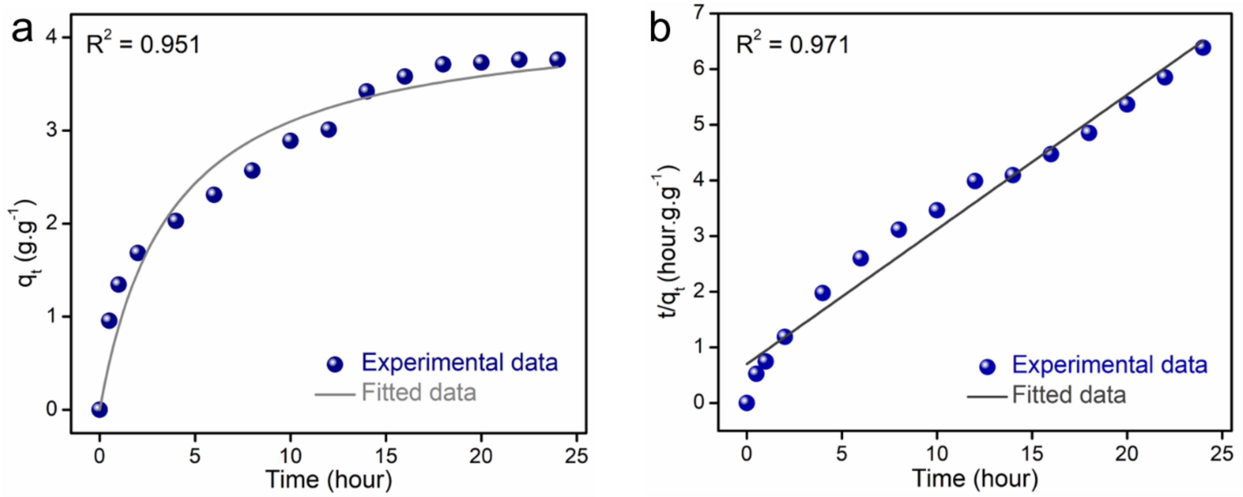

Kinetic parameters for the vapor phase dynamic iodine adsorption by IPcomp-7

q_e (exp) = 3.76 g g^{-1}

Pseudo-second-order model

$q_t = (q_e^2 k_2 t)/(1+q_e k_2 t)$

q_e (g g^{-1})	k_2 (g g^{-1} hour^{-1})	R^2
4.255	0.0627	0.951

Supplementary Figure 46: Pseudo-second-order model (a) non-linear and (b) linear fitting and kinetics parameters (from non-linear equation) for vapor phase dynamic iodine capture study by IPcomp-7 at 75 ^oC.

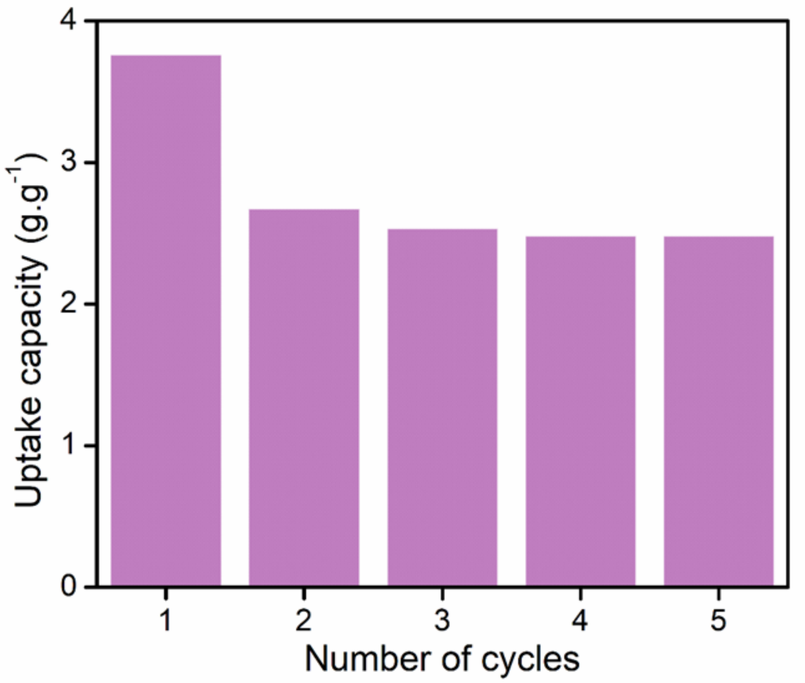

Supplementary Figure 47: The recyclability result of IPcomp-7 towards vapor phase dynamic iodine capture.

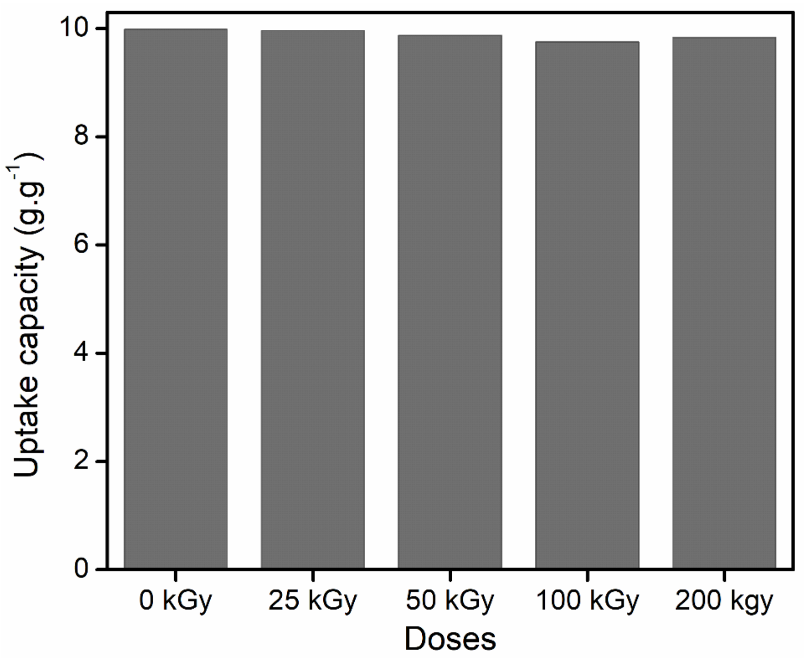

Supplementary Figure 48: Vapor phase static iodine capture capacities of IPcomp-7 at 75 ^{o}C upon exposer of various doses of irradiation.

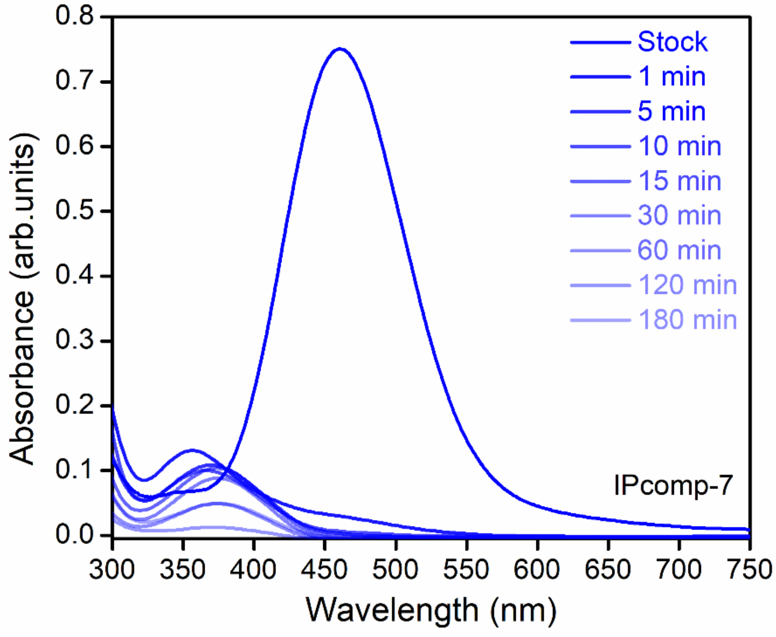

Supplementary Figure 49: Time-dependent UV-vis spectra of molecular iodine (I_2) in water showing rapid dismissing by the treatment of IPcomp-7.

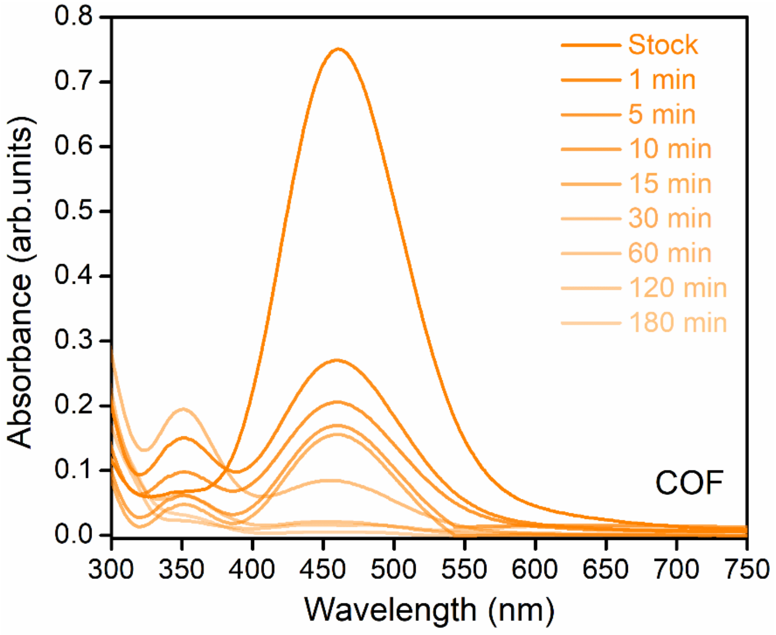

Supplementary Figure 50: Time-dependent UV-vis spectra of molecular iodine (I_2) in water showing rapid dismissing by the treatment of COF-aerogel.

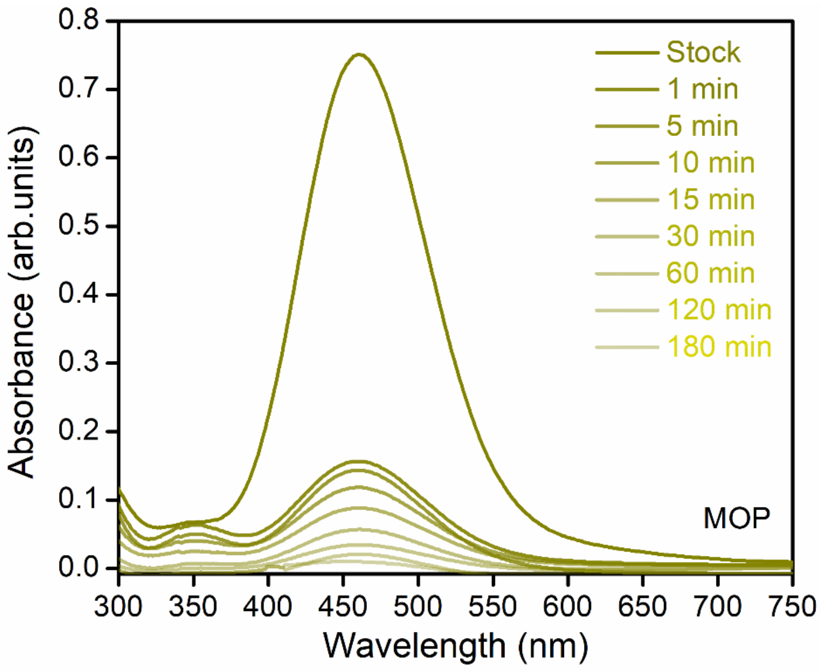

Supplementary Figure 51: Time-dependent UV-vis spectra of molecular iodine (I_2) in water showing rapid dismissing by the treatment of MOP.

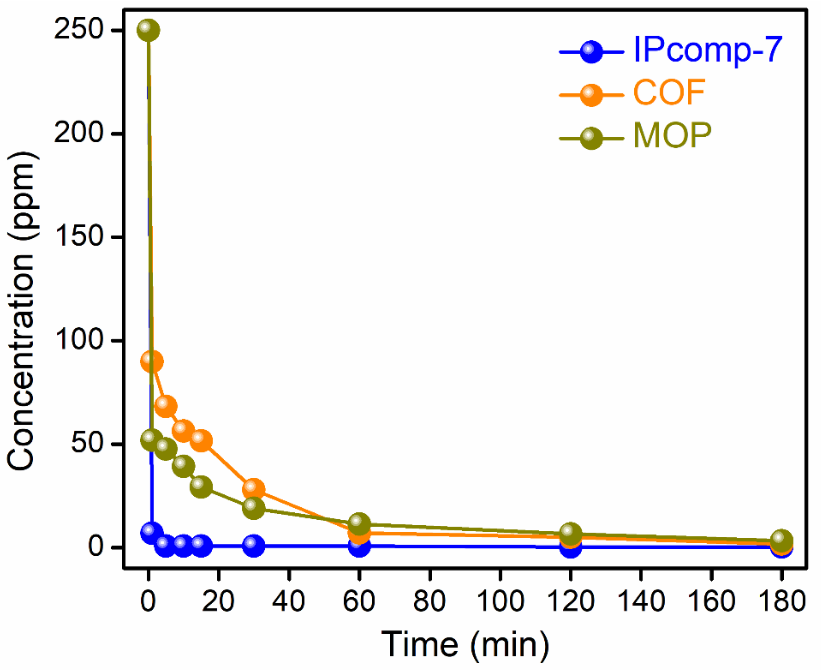

Supplementary Figure 52: Profile of decrease in the concentration of iodine in water with time by the treatment of IPcomp-7, COF-aerogel and MOP.

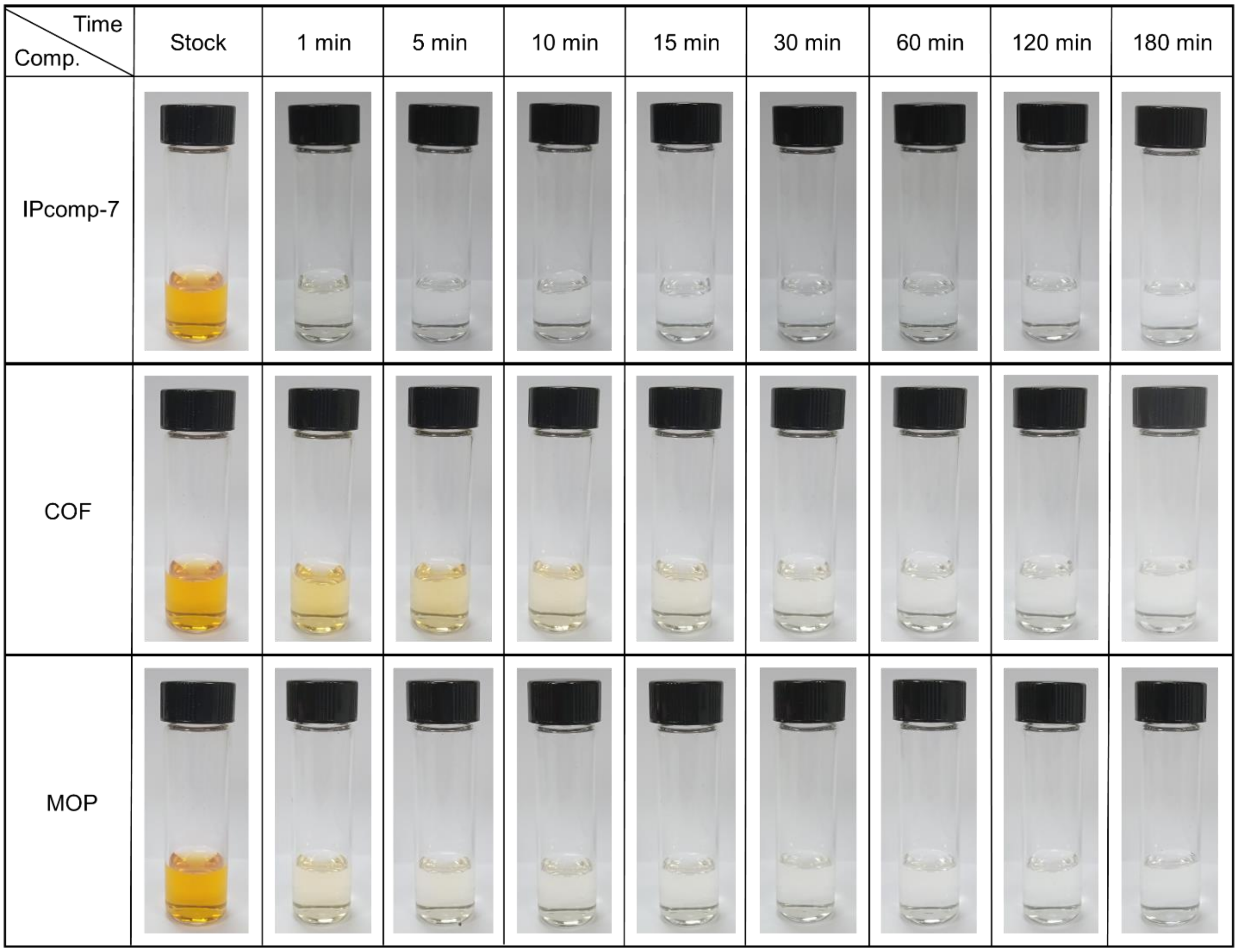

Supplementary Figure 53: Digital images of color changes of the saturated iodine aqueous solutions with time upon treatment of IPcomp-7, COF-aerogel and MOP.

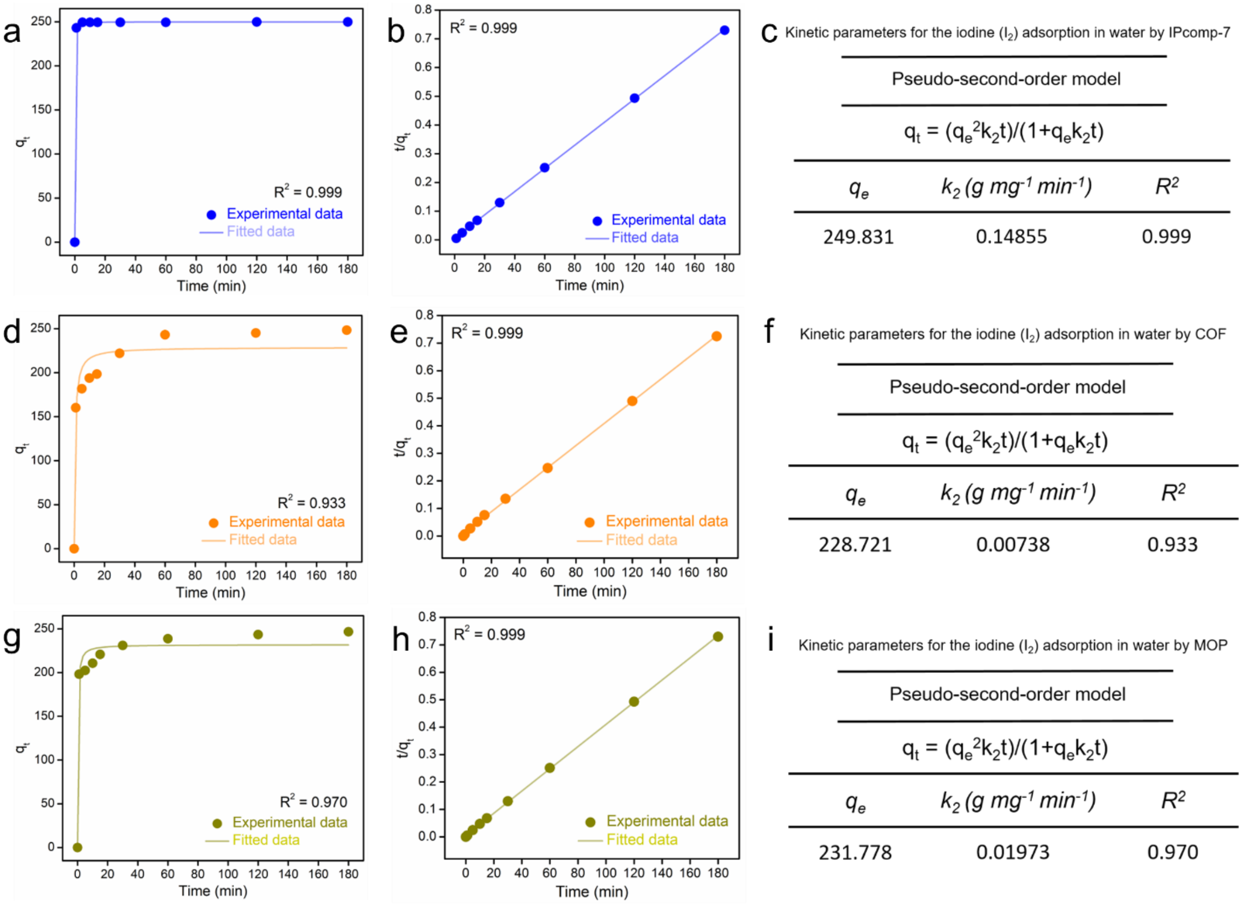

Supplementary Figure 54: Pseudo-second-order kinetic model fitting plots (nonlinear and linear) and parameters (from non-linear equation) for capture of a saturated iodine aqueous solution by (a, b, c) IPcomp-7, (d, e, f) COF-aerogel and (g, h, i) MOP.

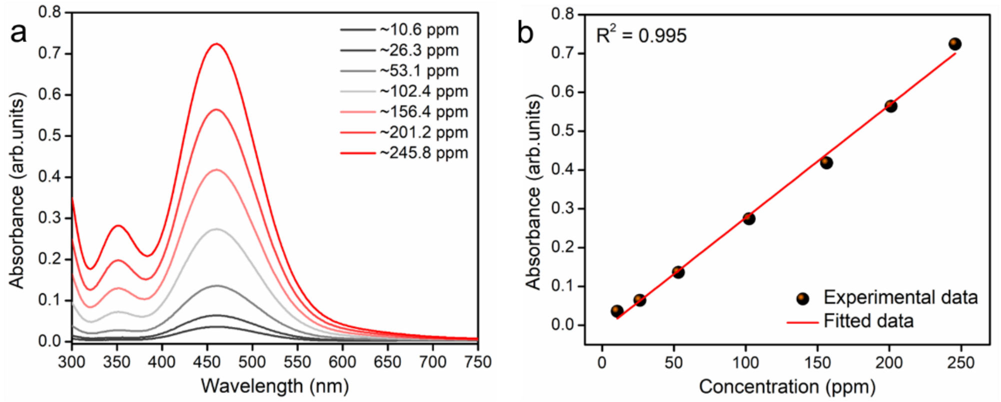

Supplementary Figure 55: (a) UV-vis spectra of aqueous solutions of iodine at different concentrations, (b) Standard curve plotted based on the corresponding absorbance.

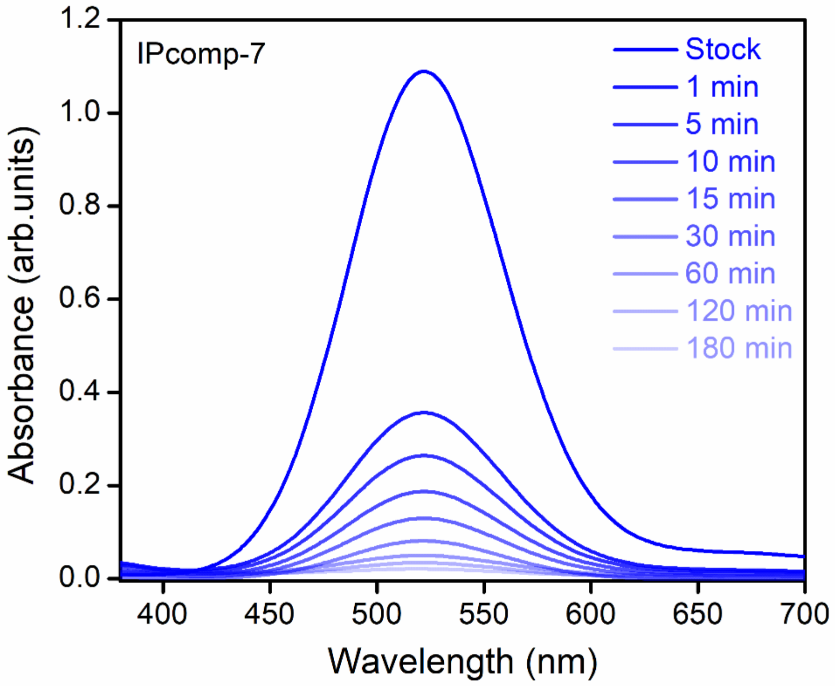

Supplementary Figure 56: Time-dependent UV-vis spectra of molecular iodine (I_2) in n-hexane showing rapid dismissing by the treatment of IPcomp-7.

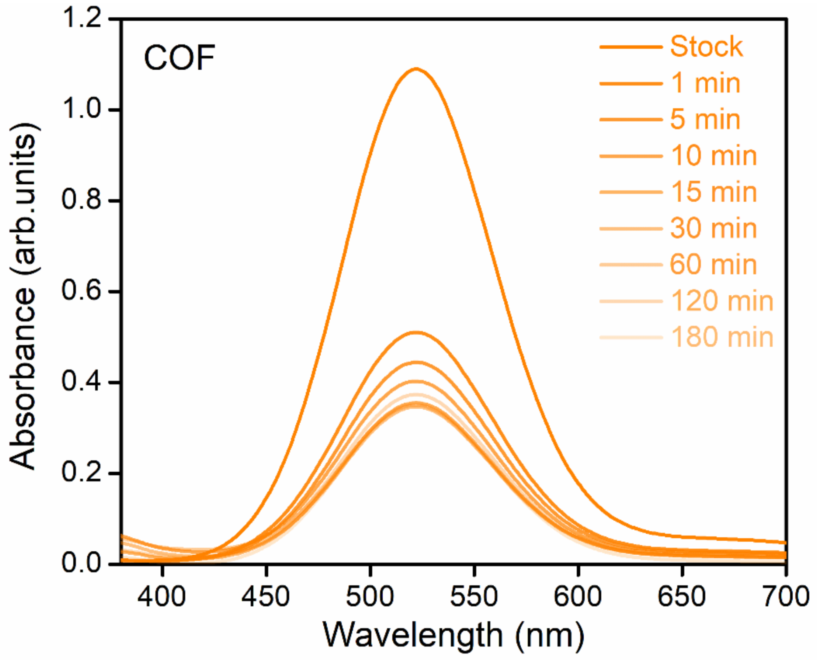

Supplementary Figure 57: Time-dependent UV-vis spectra of molecular iodine (I_2) in n-hexane showing rapid dismissing by the treatment of COF-aerogel.

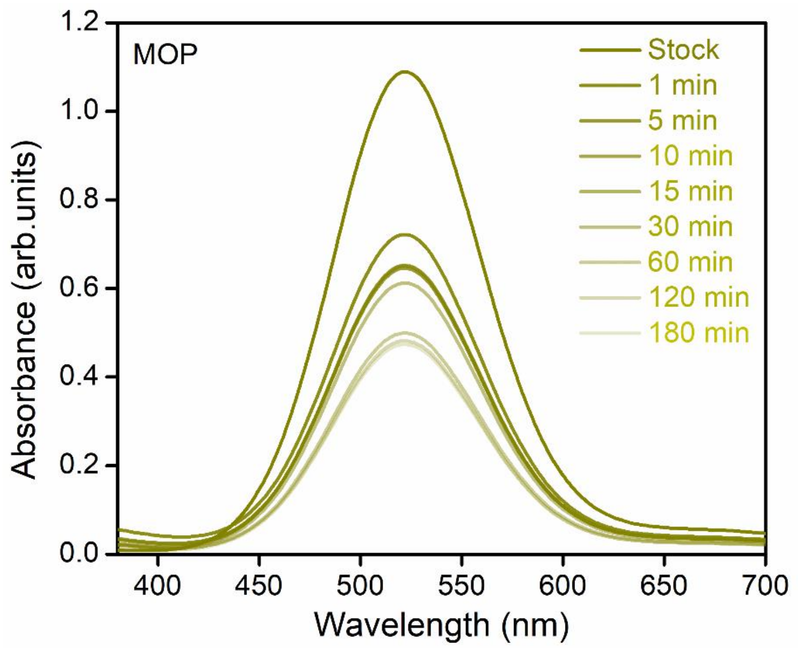

Supplementary Figure 58: Time-dependent UV-vis spectra of molecular iodine (I_2) in n-hexane showing rapid dismissing by the treatment of MOP.

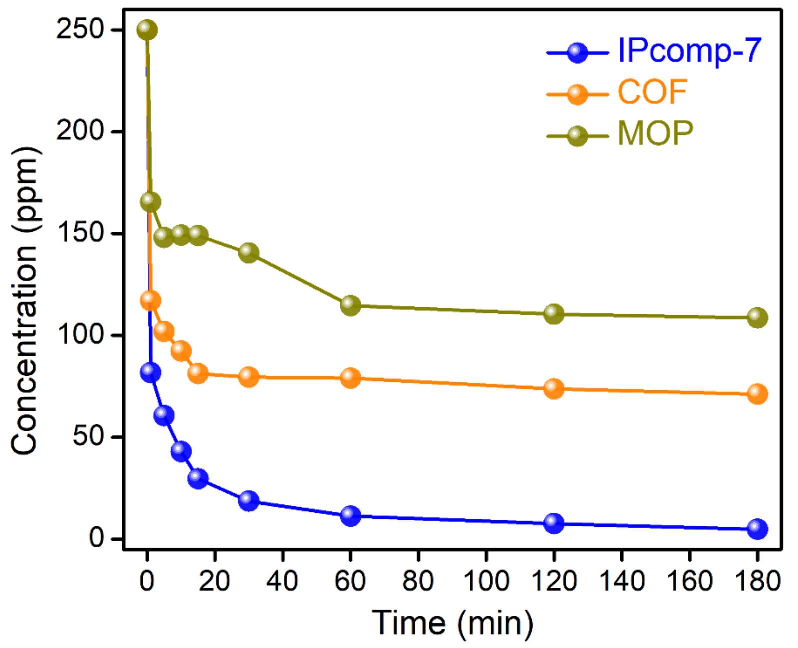

Supplementary Figure 59: Profile of decrease in the concentration of iodine in n-hexane with time by the treatment of IPcomp-7, COF-aerogel and MOP.

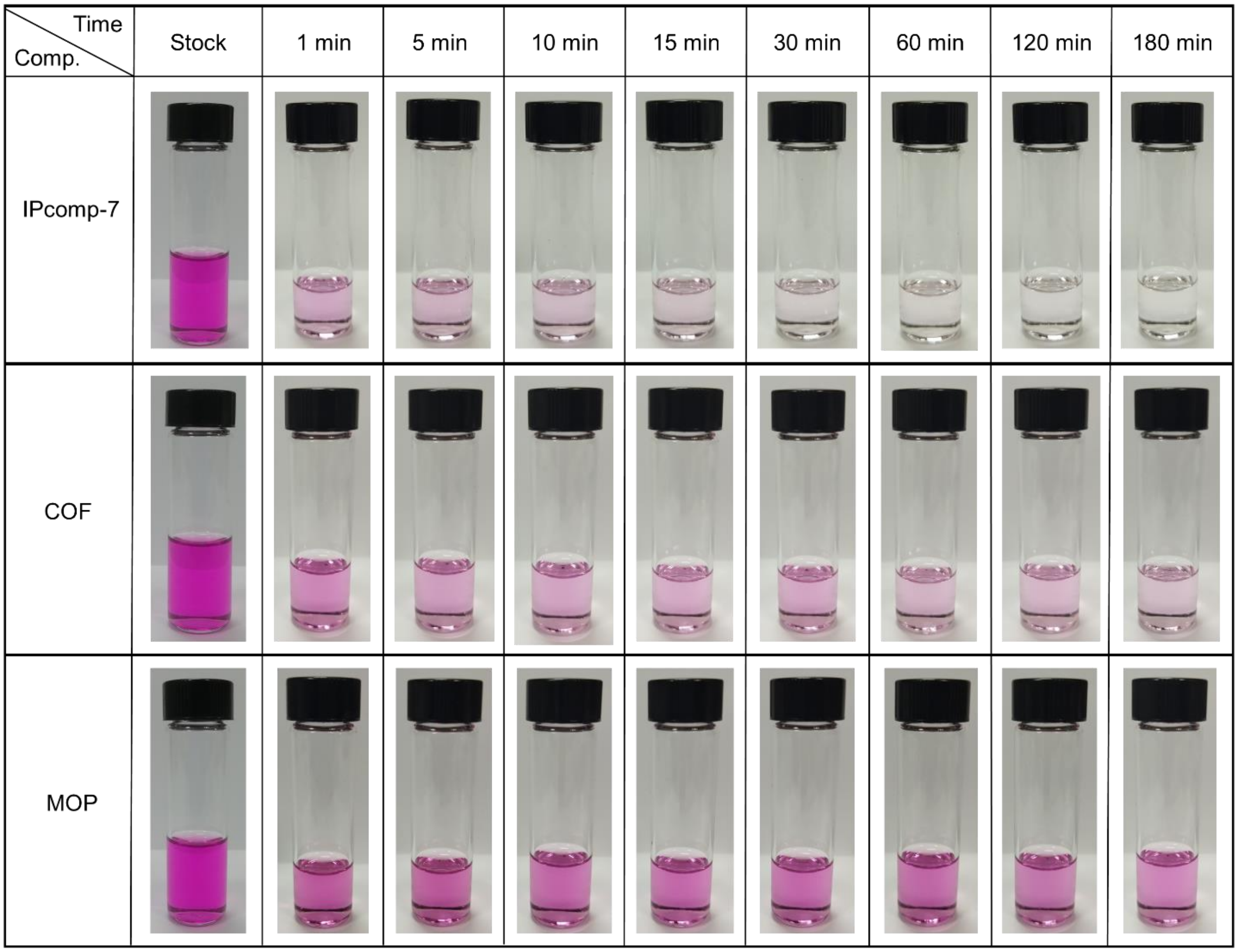

Supplementary Figure 60: Digital images of color changes of the iodine solutions in n-hexane with time upon treatment of IPcomp-7, COF-aerogel and MOP.

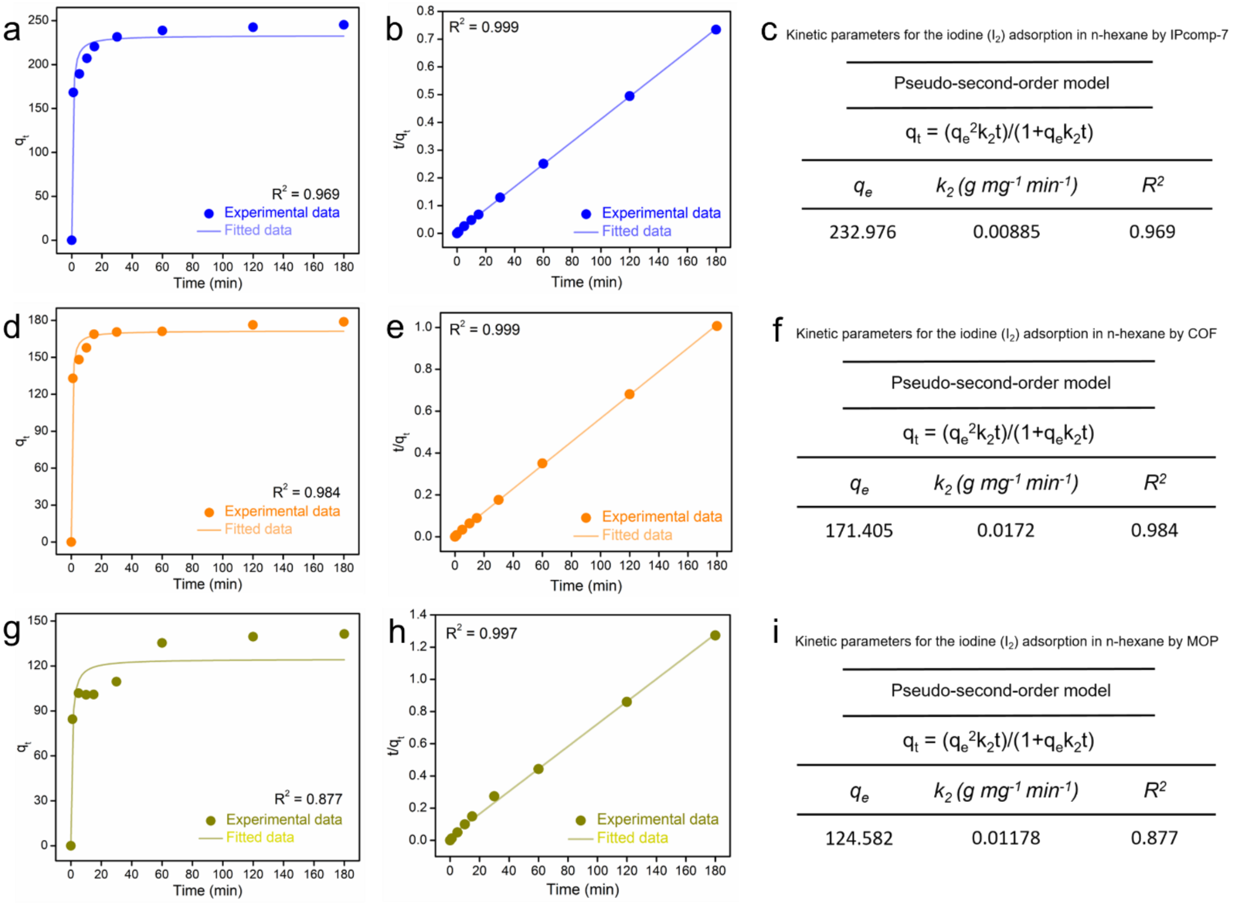

Supplementary Figure 61: Pseudo-second-order kinetic model fitting plots (linear and nonlinear) and parameters (from non-linear equation) for capture of iodine solution in n-hexane by (a, b, c) IPcomp-7, (d, e, f) COF-aerogel and (g, h, i) MOP.

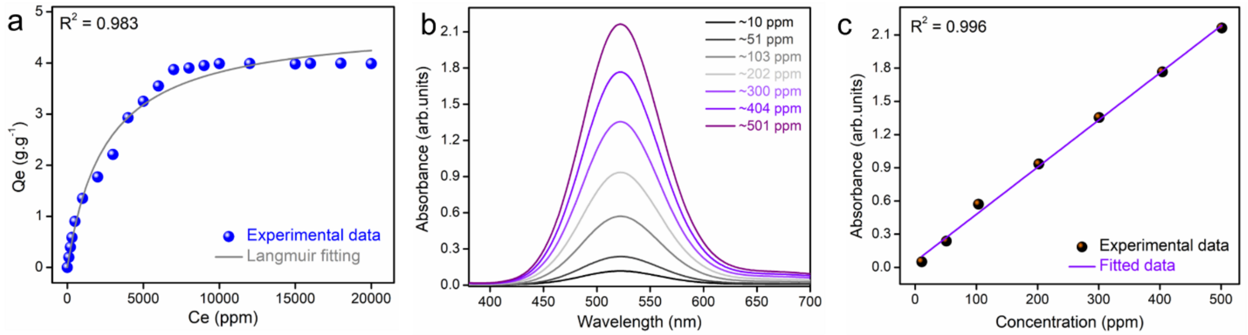

Supplementary Figure 62: (a) Concentration dependent I_2 sorption isotherm fitting with Langmuir model, (b) UV-vis spectra of n-hexane solutions of iodine at different concentrations, (c) Standard curve plotted based on the corresponding absorbance.

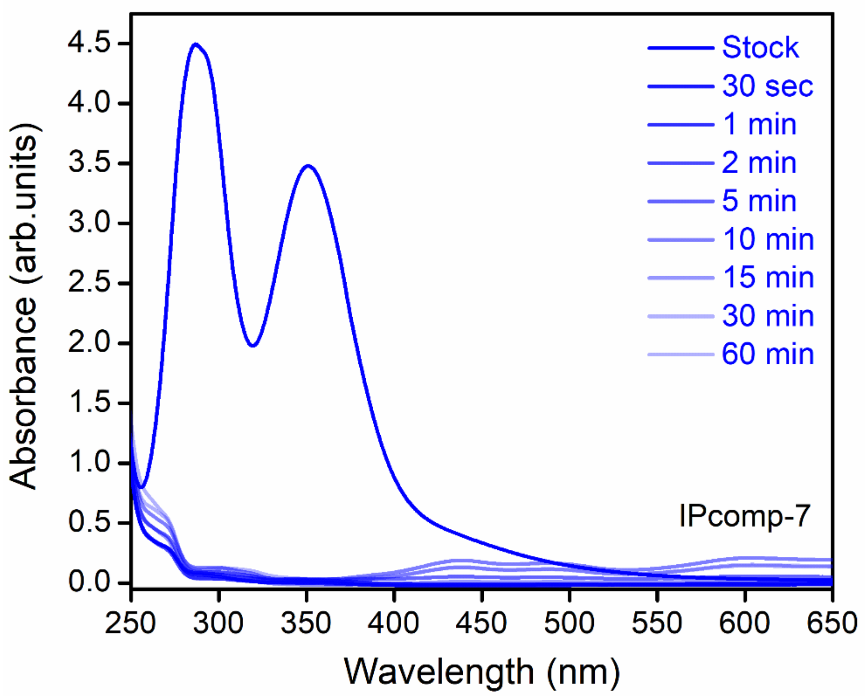

Supplementary Figure 63: Time-dependent UV-vis spectra of triiodides (I_3^-) (KI/I_2) in water showing rapid dismissing by the treatment of IPcomp-7.

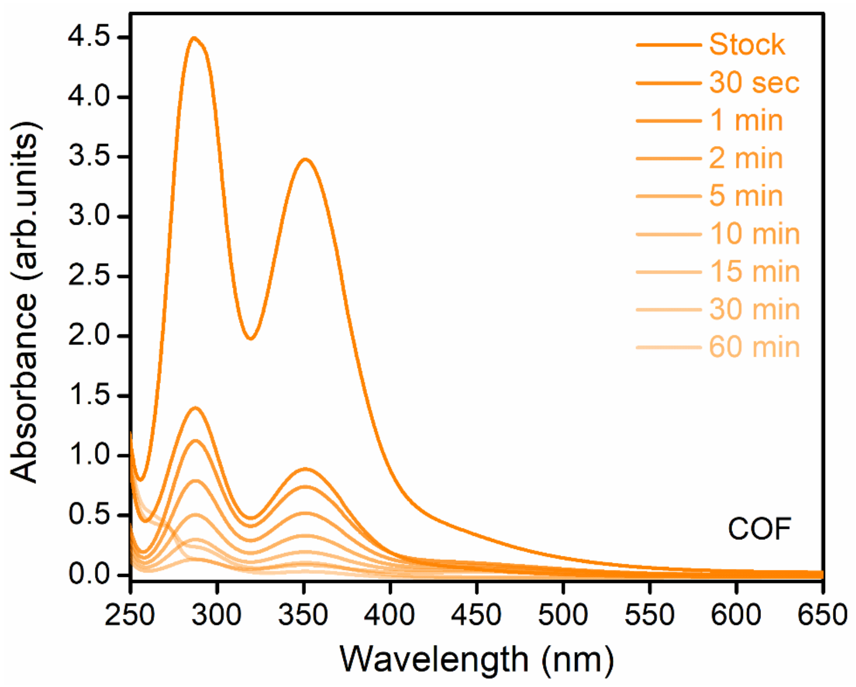

Supplementary Figure 64: Time-dependent UV-vis spectra of triiodides (I_3^-) (KI/I_2) in water showing rapid dismissing by the treatment of COF-aerogel.

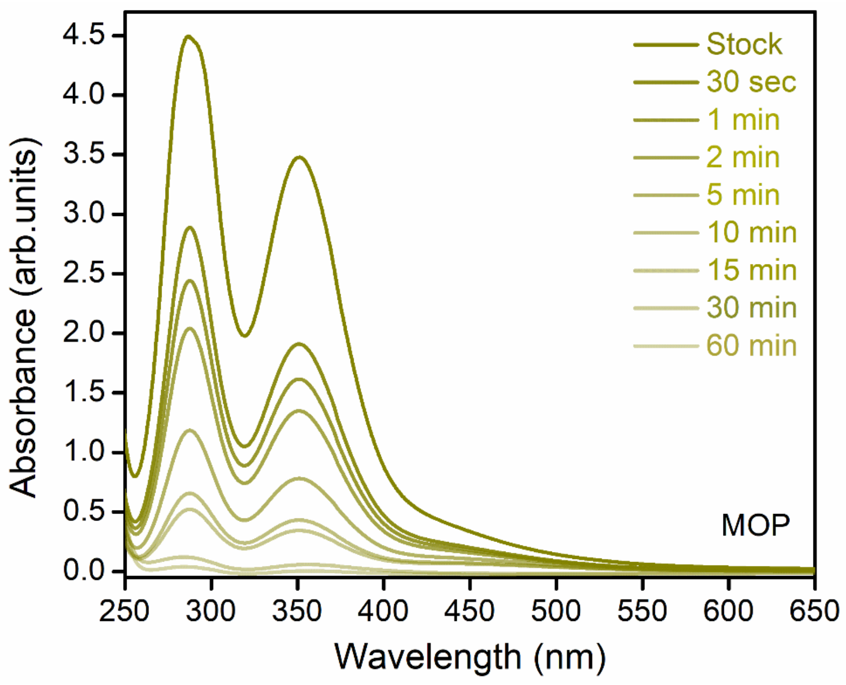

Supplementary Figure 65: Time-dependent UV-vis spectra of triiodides (I_3^-) (KI/I_2) in water showing rapid dismissing by the treatment of MOP.

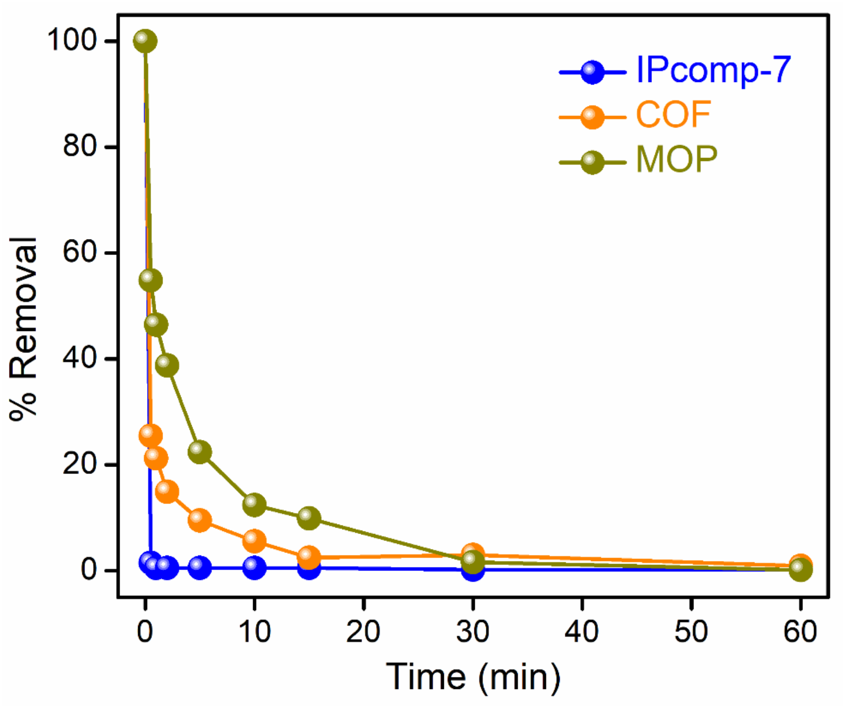

Supplementary Figure 66: Profile of decrease in the concentration of triiodides (I_3^-) (KI/I_2) in water with time by the treatment of IPcomp-7, COF-aerogel and MOP.

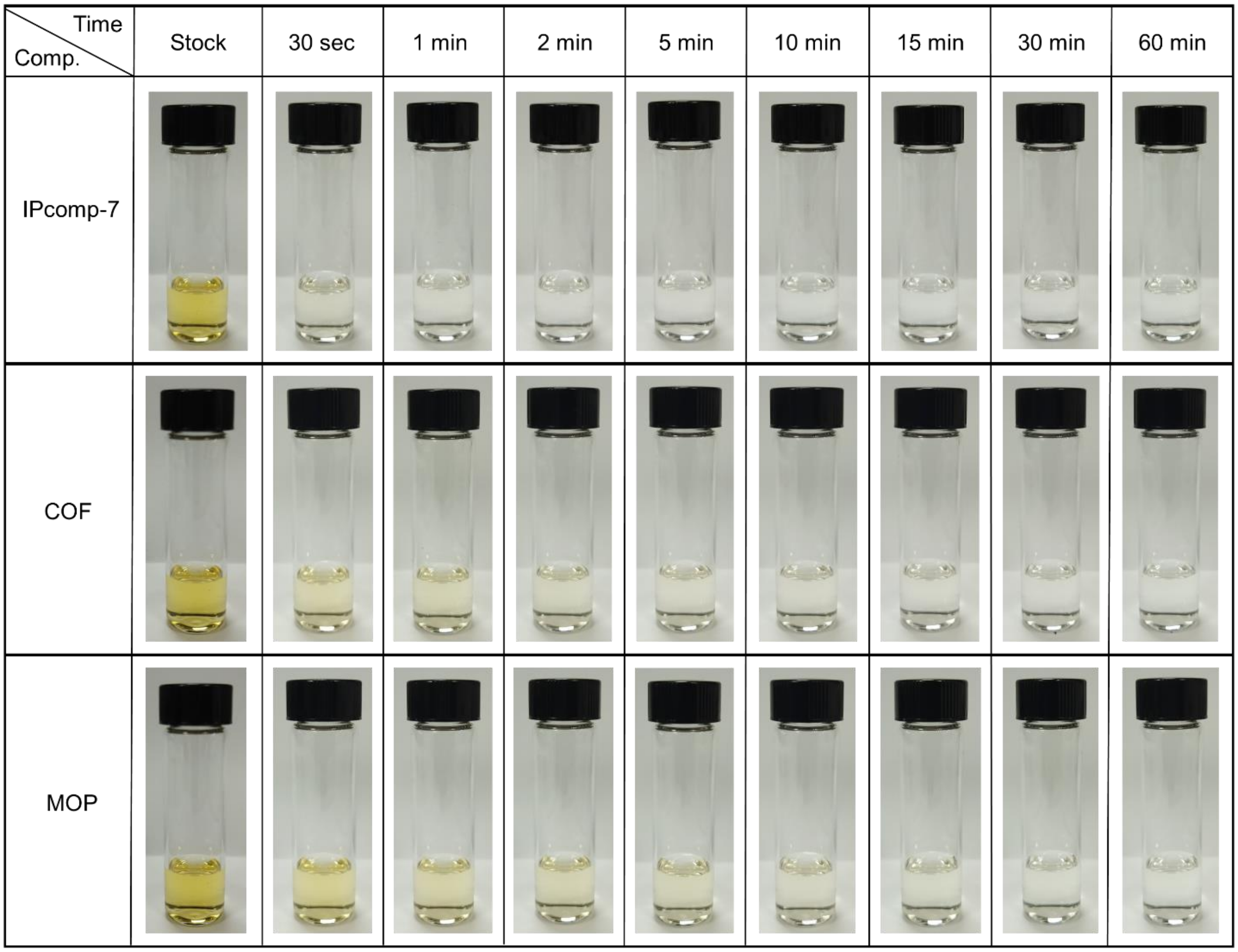

Supplementary Figure 67: Digital images of color changes of the triiodides (I_3^-) (KI/I_2) solution in water with time upon treatment of IPcomp-7, COF-aerogel and MOP.

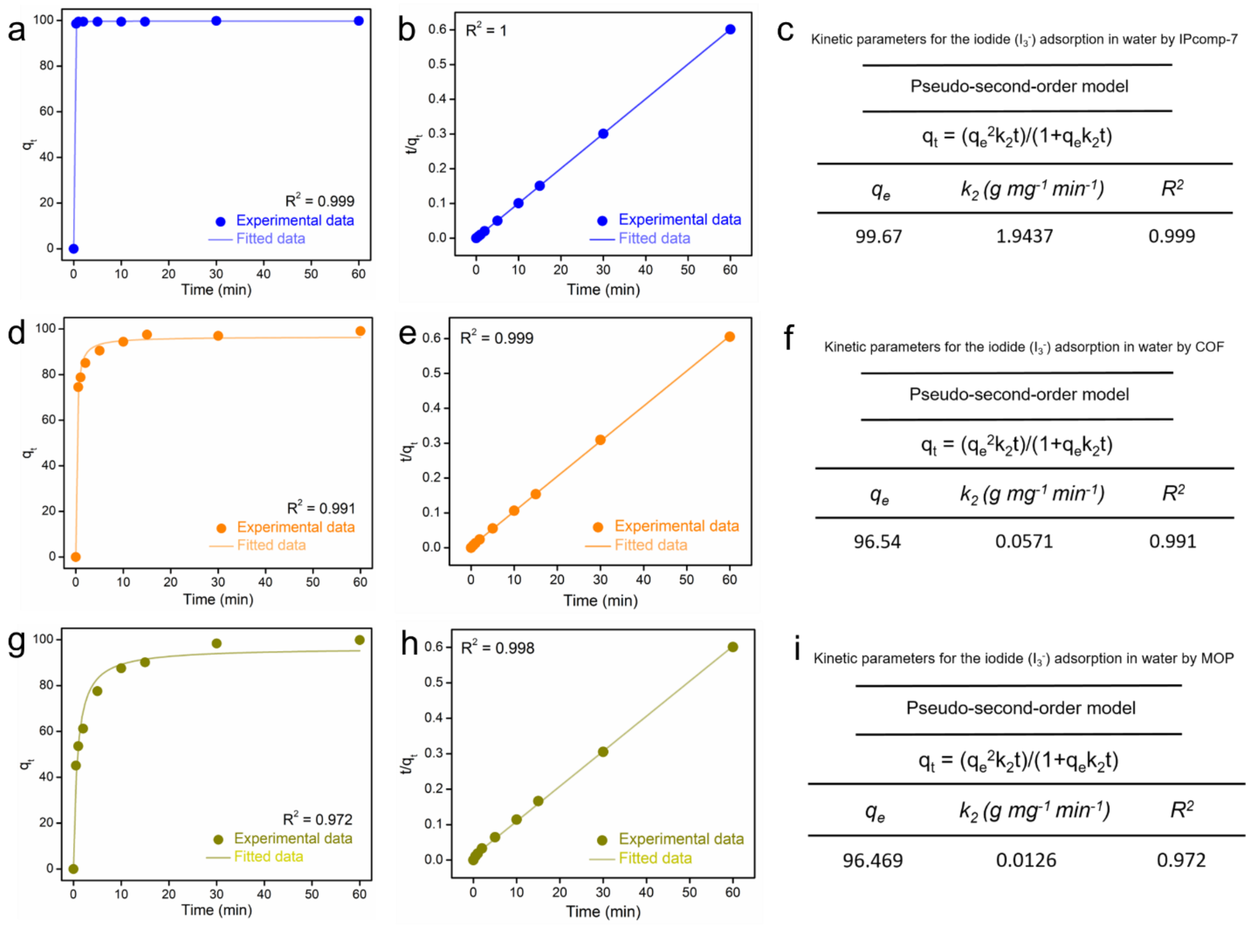

Supplementary Figure 68: Pseudo-second-order kinetic model fitting plots (linear and nonlinear) and parameters (from non-linear equation) for capture of triiodides (I_3^-) (KI/I_2) solution in water by (a, b, c) IPcomp-7, (d, e, f) COF-aerogel and (g, h, i) MOP.

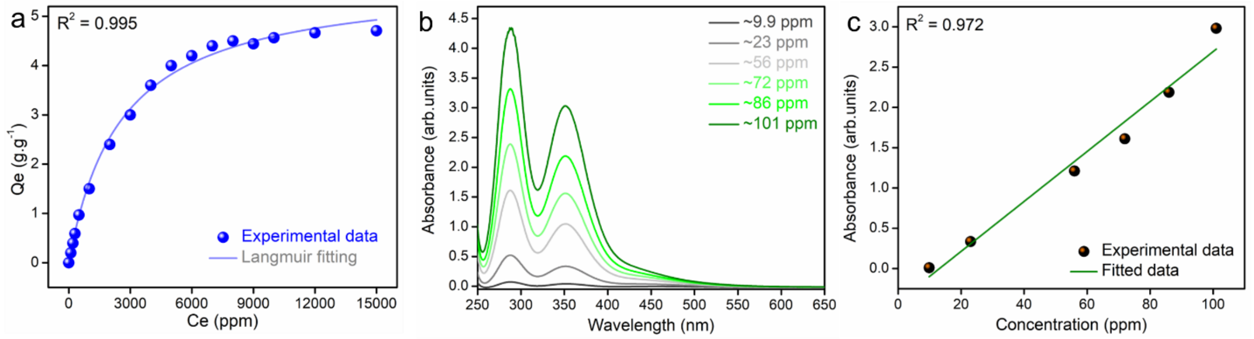

Supplementary Figure 69: (a) Concentration dependent I_3^- sorption isotherm fitting with Langmuir model, (b) UV-vis spectra of aqueous solutions of triiodides (I_3^-) (KI/I_2) at different concentrations, (c) Standard curve plotted based on the corresponding absorbance.

Supplementary Table 8: Comparison table of solution phase iodine adsorption capacities of various materials.

Materials	Medium	Capacity ($g.g^{-1}$)	I_2/I_3^-	Material type	Ref.
IPcomp-7	Water	5.16	I_2/KI	Hybrid composite	This work
IPcomp-7	Water	4.74	I_2	Hybrid composite	This work
C[4]P-BTP	Water	3.24	I_2/KI	POP	17
C[4]P-BTP	Water	3.03	I_2	POP	
C[4]P-TPE	Water	2.99	I_2/KI	POP	
C[4]P-BT	Water	2.32	I_2/KI	POP	
C[4]P-TTP	Water	2.51	I_2/KI	POP	
C[4]P-BP	Water	2.37	I_2/KI	POP	
C[4]P-DPP	Water	1.58	I_2/KI	POP	
HcOF-1	Water	2.10	I_2/KI	HOF	41
HcOF-2	Water	3.2	I_2/KI	HOF	16
HcOF-3	Water	3.0	I_2/KI	HOF	
HcOF-4	Water	3.6	I_2/KI	HOF	
HcOF-7	Water	1.39	I_2/KI	HOF	42
CaCOP1	Water	2.4	I_2	POP	43
CaCOP2	Water	2.81	I_2	POP	
CaCOP3	Water	3.1	I_2	POP	
3D MOF-1	Water	1.10	I_2/KI	MOF	44
MBM-MOF	Water	0.88	I_2/KI	MOF	45
Cd(II)-triazole MOF	Water	0.18	I_2/KI	MOF	46
G-QP6	Water	0.247	I_2/KI	POP	47
G-QP5	Water	0.245	I_2/KI	POP	
G-TP-5	Water	0.243	I_2/KI	POP	
G-TP6	Water	0.24	I_2/KI	POP	
COFP-1	Water	5.05	I_2	COF	48
COFP-1	Water	4.81	I_2/KI	COF	
COFA-1	Water	6.78	I_2	COF aerogel	
COFA-1	Water	7.13	I_2/KI	COF aerogel	
Compound-1	Water	3.5	I_2/KI	POP	49
Compound-2	Water	3.04	I_2/KI	POP	
CalCOP-1	Water	2.318	I_2/KI	POP	50
CalCOP-2	Water	1.758	I_2/KI	POP	

THPS-C	Water	0.926	I_2/KI	POP	51
DTTP5	Water	0.0943	I_2/KI	POP	52
IPcomp-7	Hexane	4.23	I_2	Hybrid composite	This work
COFP-1	Hexane	4.15	I_2	COF	48
COFA-1	Hexane	6.90	I_2	COF aerogel	
THPS-C	Hexane	0.93	I_2	POP	51
IL@PCN-333(Al)	Hexane	3.40	I_2	Composite	38
TatPOP-2	Hexane	0.12	I_2	POP	53
Me1POP-2	Hexane	0.76	I_2	POP	
SCMP-II	Cyclohexane	0.32	I_2	POP	54
$Zn_9(btc)_4(atz)_{12}$	Cyclohexane	0.40	I_2	MOF	55
[Bmim][Br]	Cyclohexane	2.10	I_2	Ionic Liquid	56

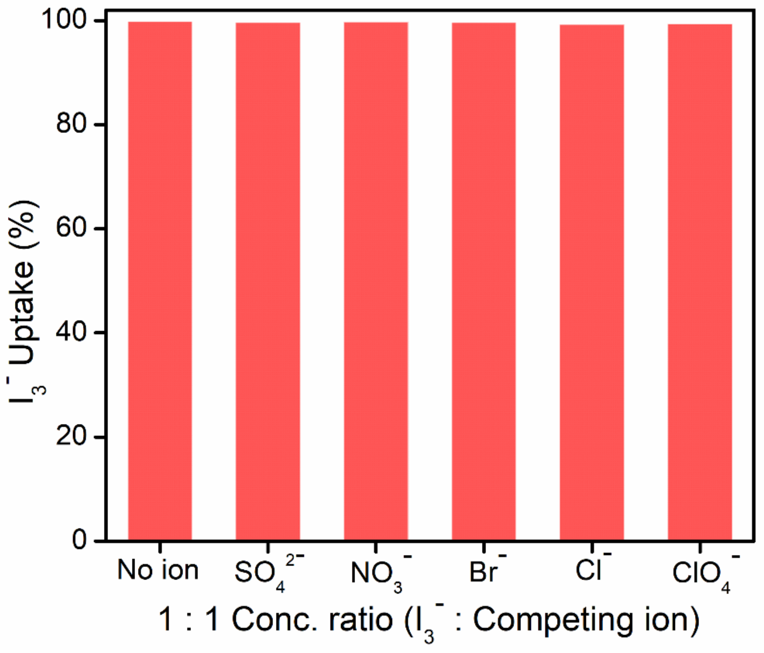

Supplementary Figure 70: Selectivity test: relative uptake for I_3^- anions in equimolar binary mixture of competing anions.

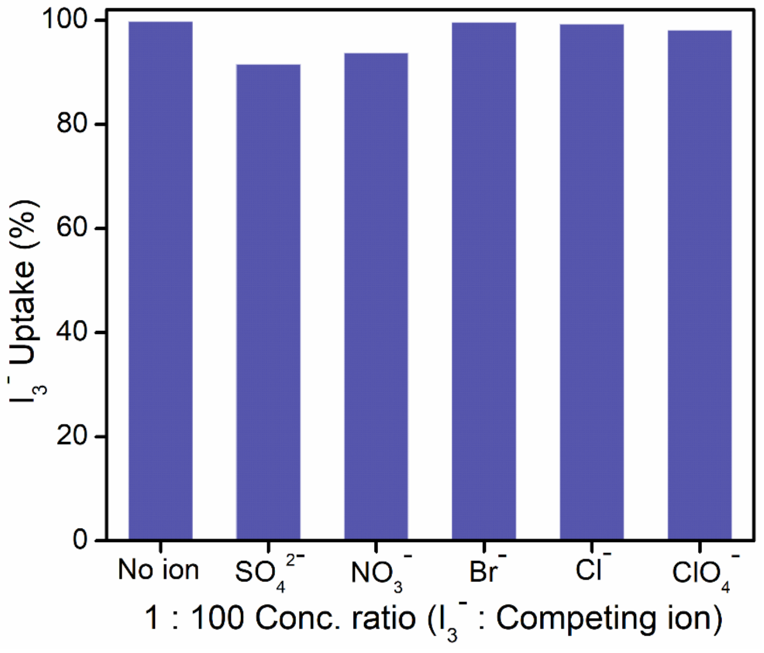

Supplementary Figure 71: Selectivity test: relative uptake for I_3^- anions in ~100-fold excess binary mixture of competing anions.

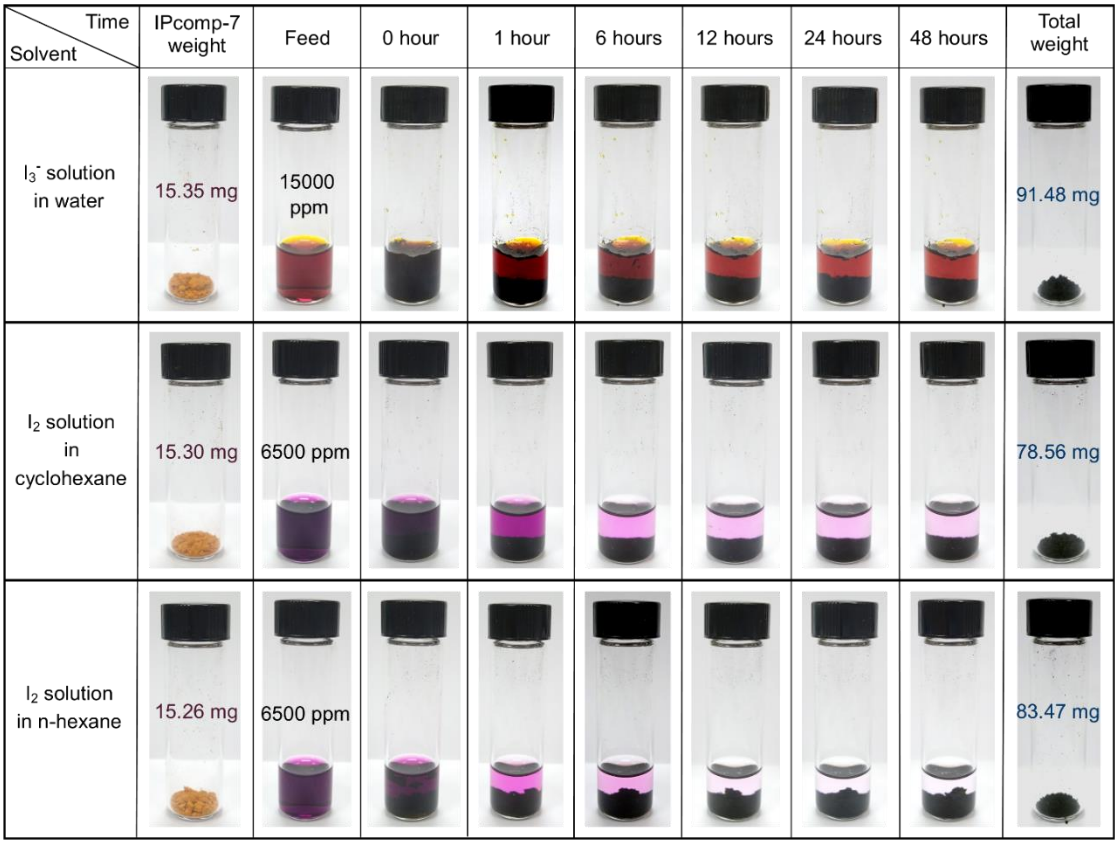

Supplementary Figure 72: Digital images of I_2 or I_3^- uptake study by IPcomp-7 in different solvents, 15000 ppm I_2/KI in water, 6500 ppm I_2 in cyclohexane, and 6500 ppm I_2 in n-hexane. [Condition: solution 2 mL, time 48 hours].

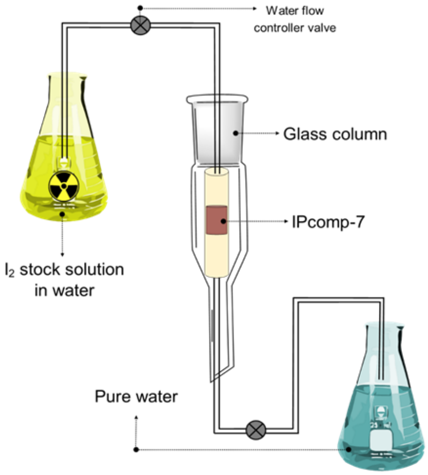

Supplementary Figure 73: Schematic representation of column-based aqueous phase iodine capture experiments.

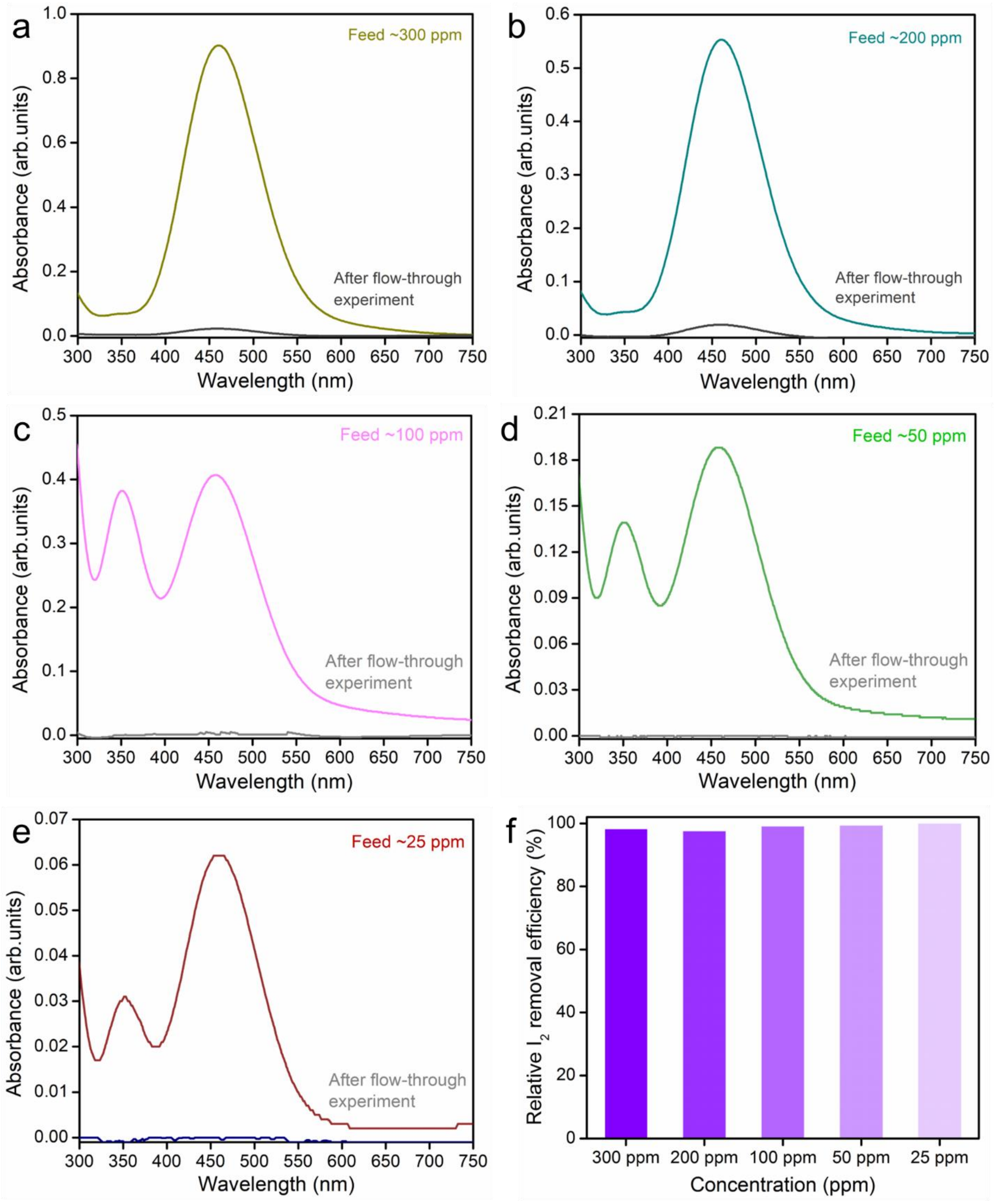

Supplementary Figure 74: (a-e) UV-vis spectra of aqueous solutions of iodine at different concentrations, before and after column-based capture experiments. (f) Relative I_2 uptake efficiency of IPcomp-7 embedded column at different concentrations.

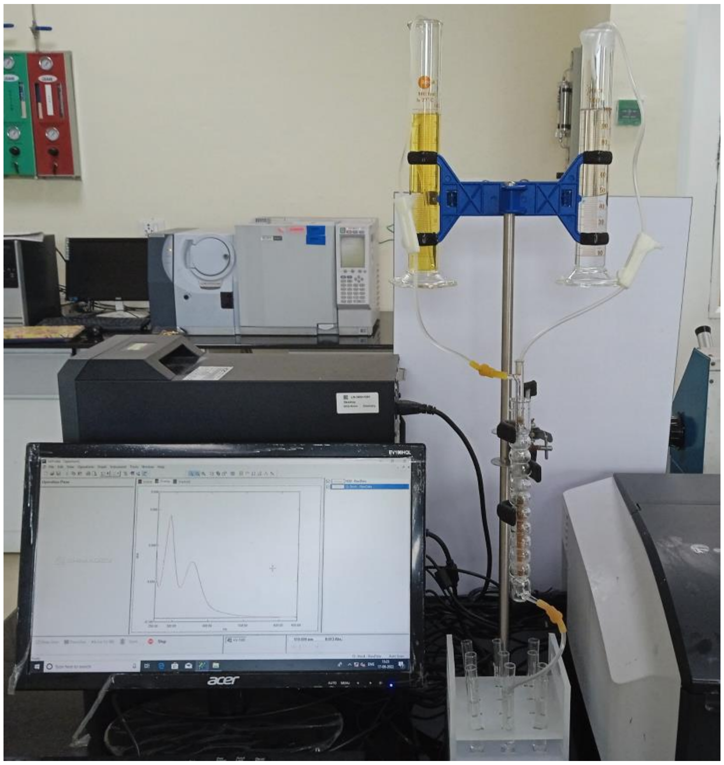

Supplementary Figure 75: Digital image of IPcomp-7 embedded column-based flow-through I_2/KI capture experiment.

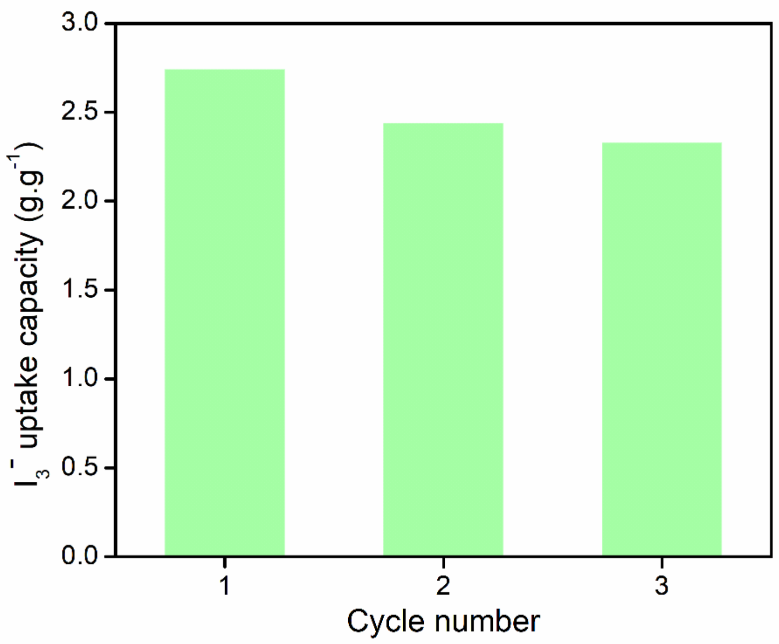

Supplementary Figure 76: Iodide uptake capacities in different cycles in case of column-based flow-through I_2/KI capture experiment.

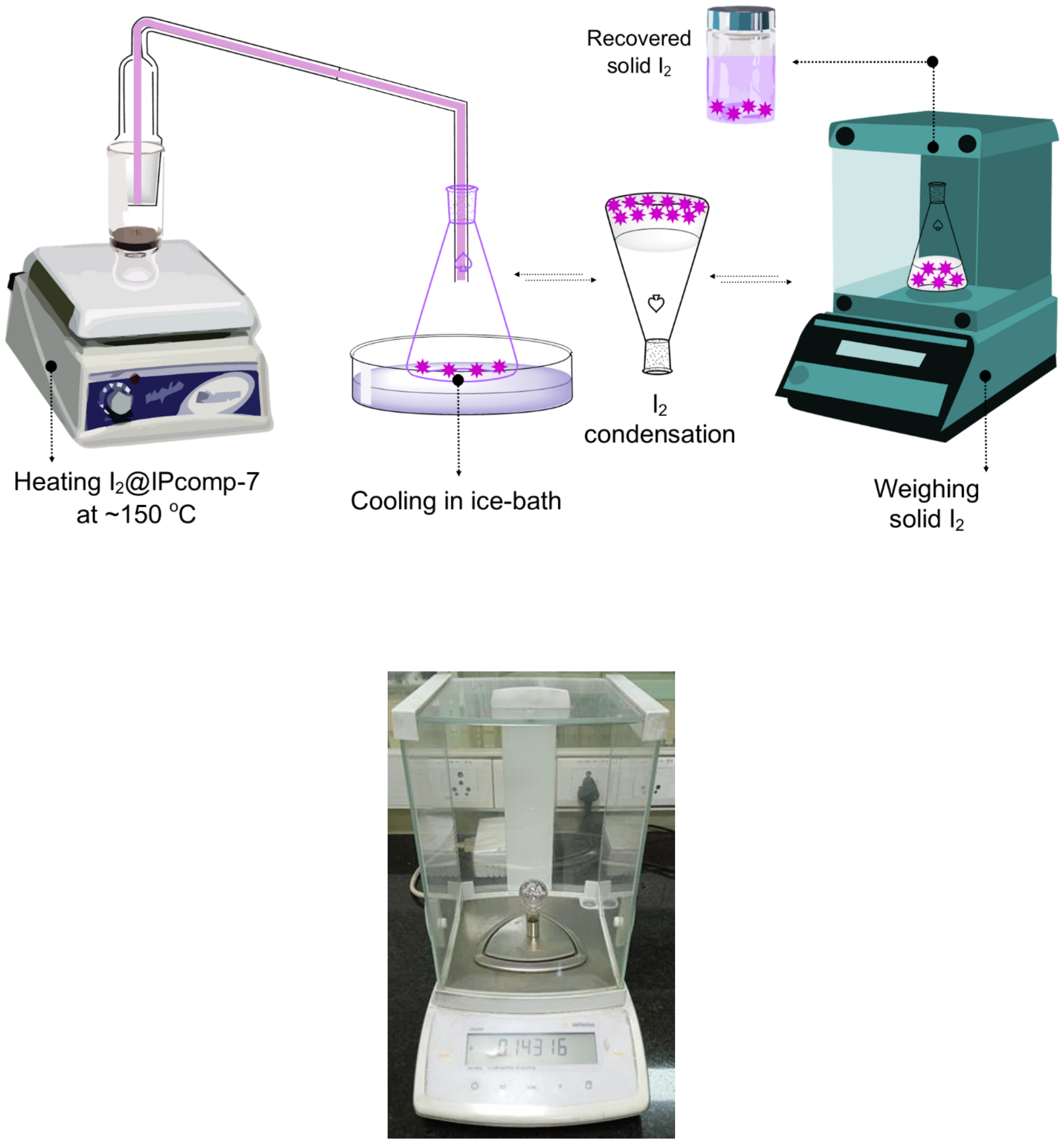

Supplementary Figure 77: Schematic representation and digital image of recovery of solid iodine setup by heating of I_2@IPcomp-7 at 150 ^oC.

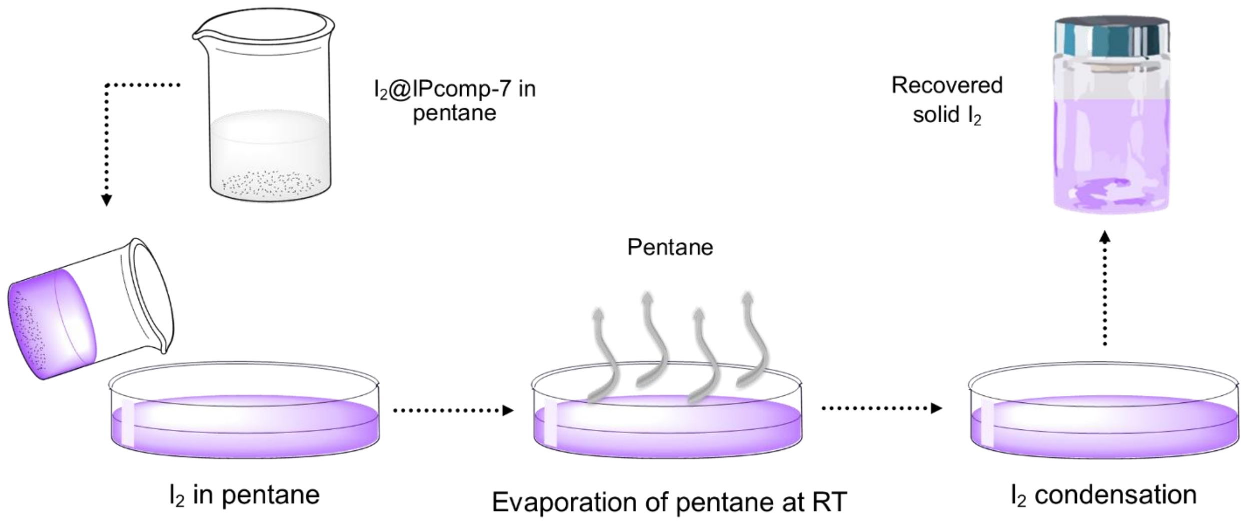

Supplementary Figure 78: Schematic representation of recovery of solid iodine crystals by treating of I_2@IPcomp-7 in pentane solvent.

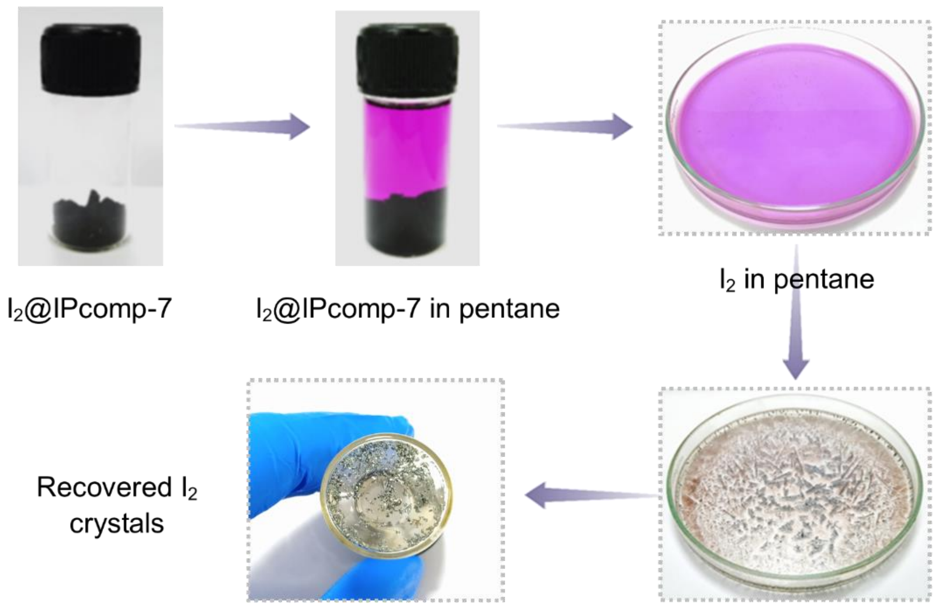

Supplementary Figure 79: Digital images of recovery of solid iodine crystals by treating of I_2@IPcomp-7 in pentane solvent.

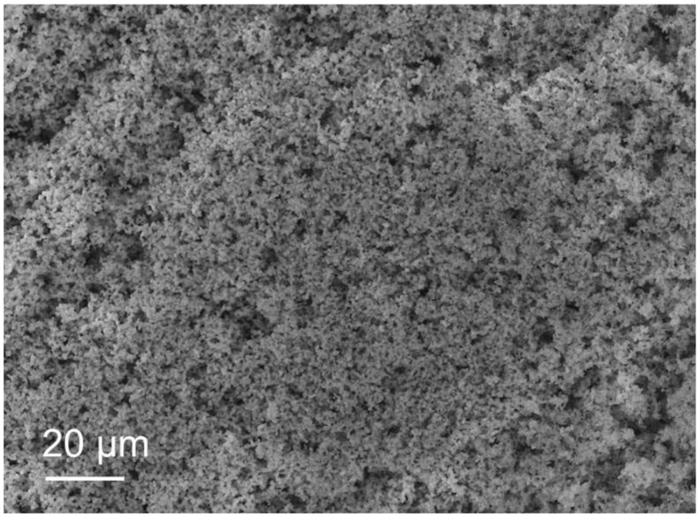

Supplementary Figure 80: FESEM image of IPcomp-7 after I_2 absorption test.

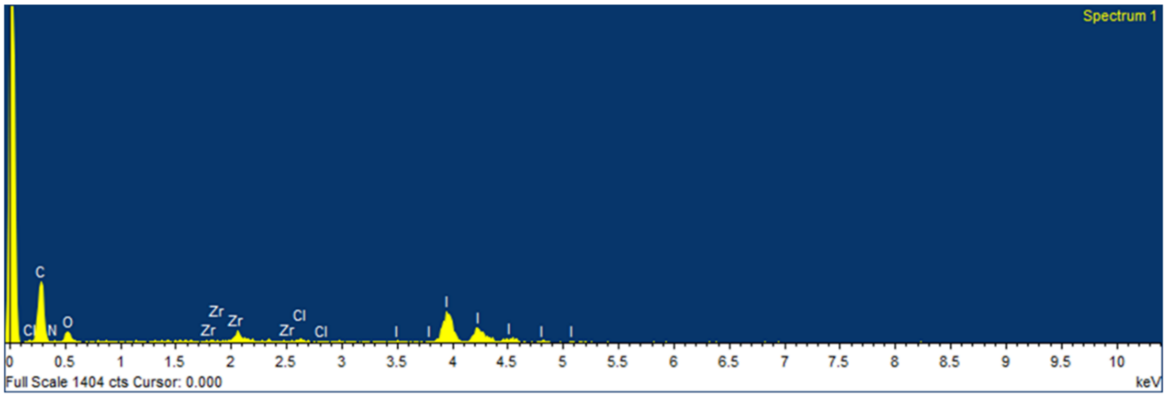

Element	Weight%	Atomic%
C K	40.55	79.50
N K	0.05	0.08
O K	6.67	9.82
Cl K	0.85	0.57
Zr L	5.50	1.42
I L	46.37	8.61
Totals	100.00	

Supplementary Figure 81: FESEM-EDX data of IPcomp-7 after I_2 absorption test.

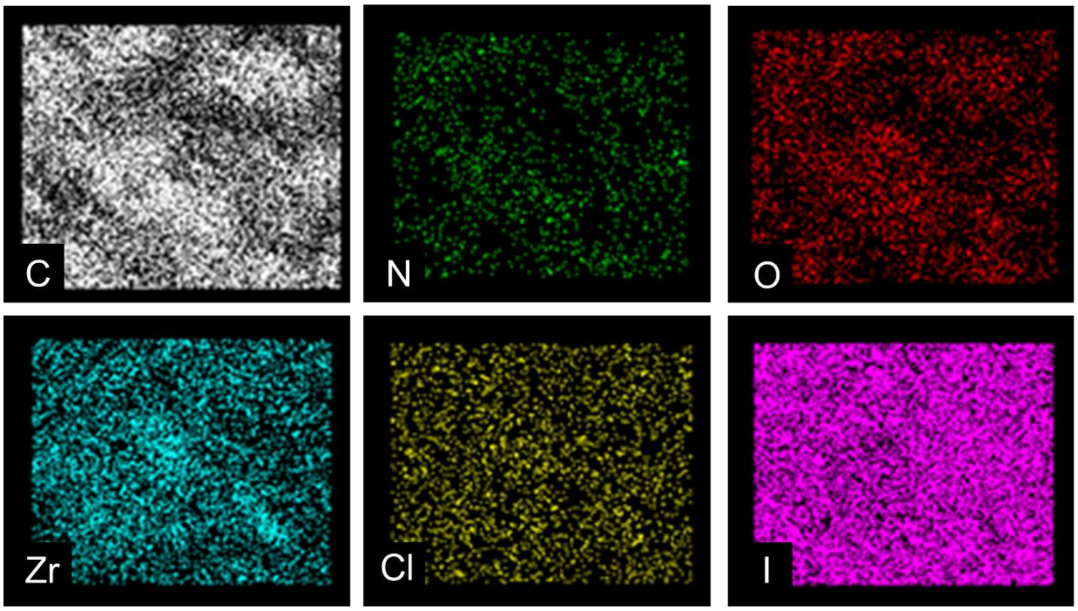

Supplementary Figure 82: Elemental mapping images from FESEM experiment of IPcomp-7 after I_2 absorption test.

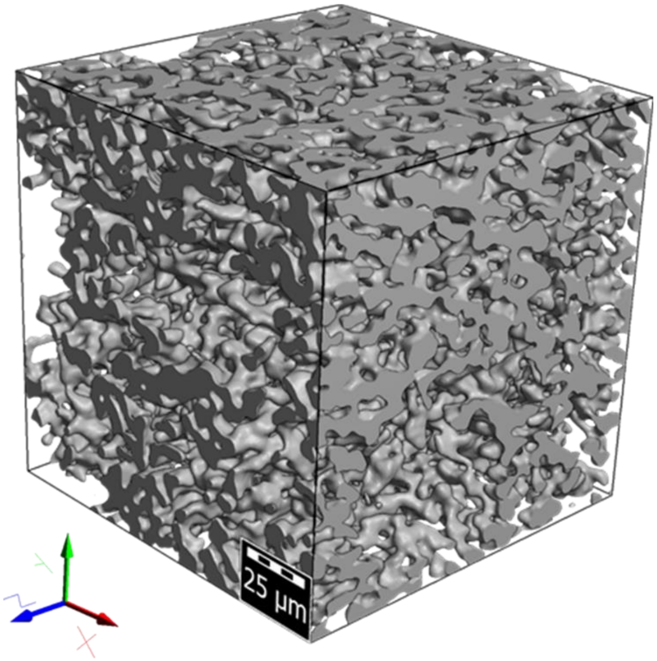

Supplementary Figure 83: X-ray 3D tomographic image of IPcomp-7 after I_2 absorption test.

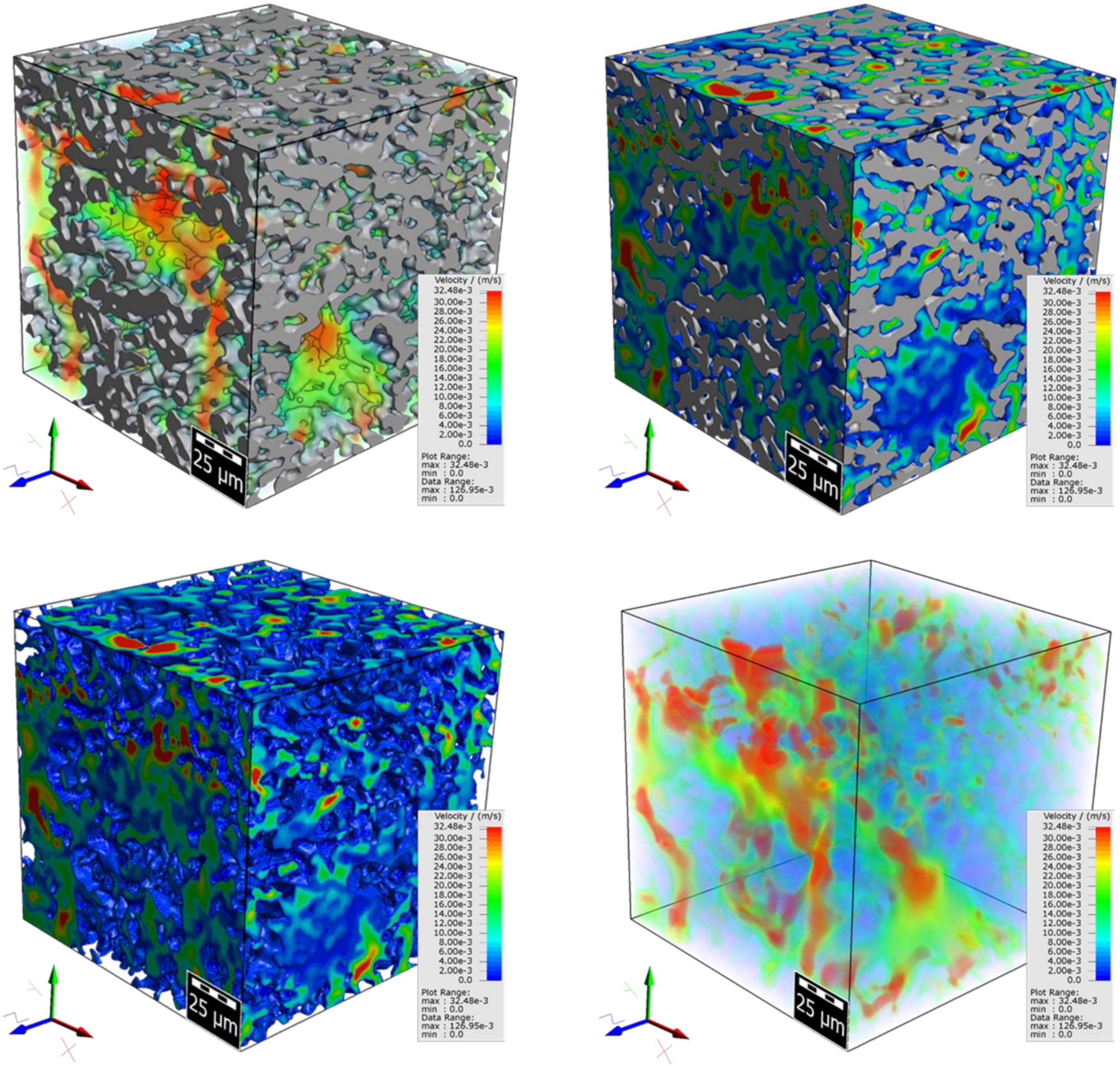

Supplementary Figure 84: Color-coded X-ray 3D tomographic image of I_2@IPcomp-7 showing the retention of micron size pores even after the iodine adsorption.

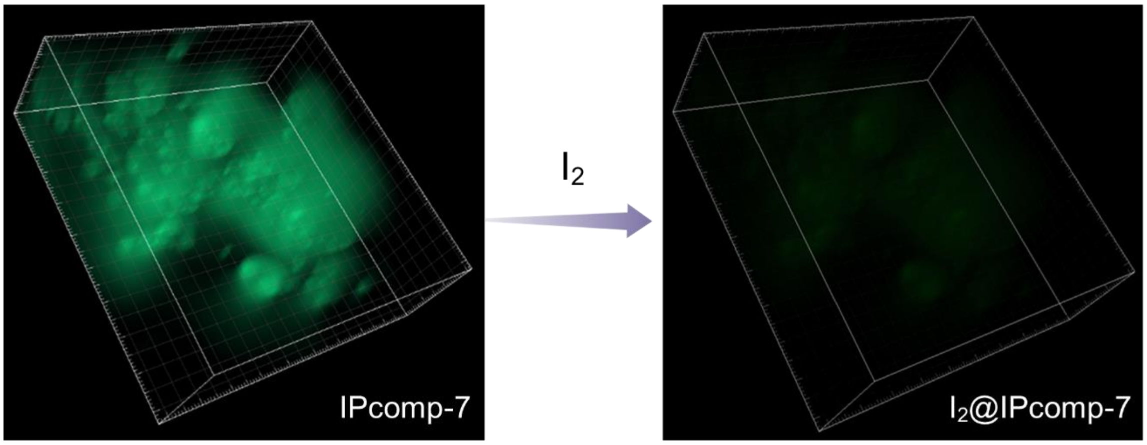

Supplementary Figure 85: Fluorescence microscopic images of IPcomp-7 before and after I_2 absorption test.

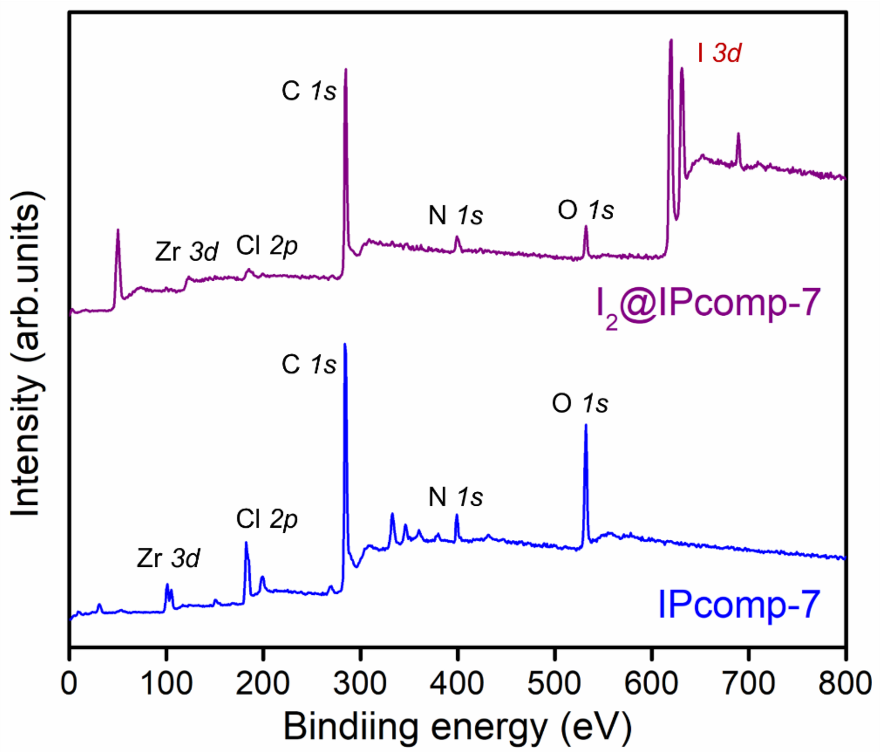

Supplementary Figure 86: XPS survey spectra of IPcomp-7 before and after I_2 absorption test.

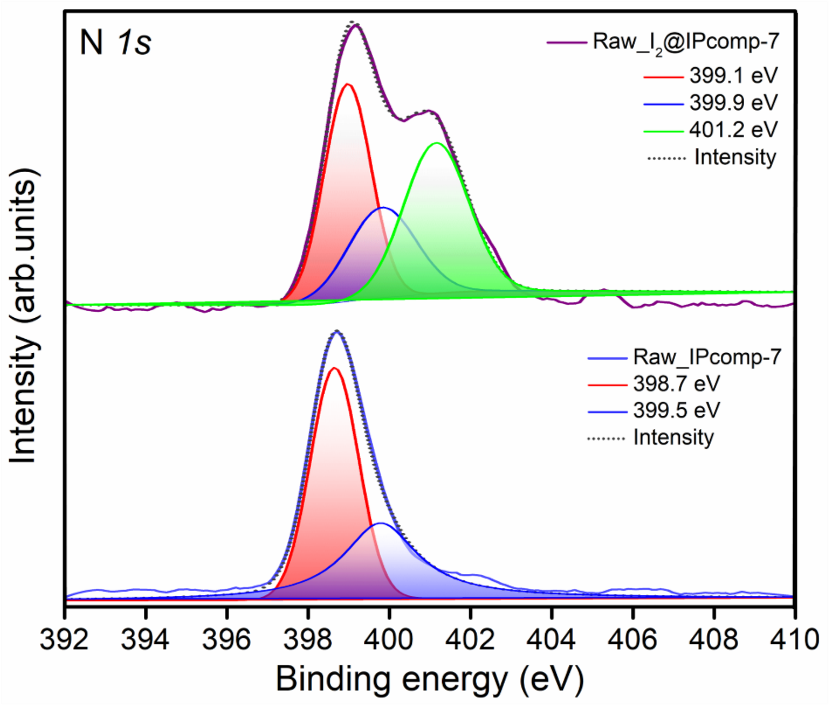

Supplementary Figure 87: N *1s* XPS spectra of IPcomp-7 before and after I_2 absorption test.

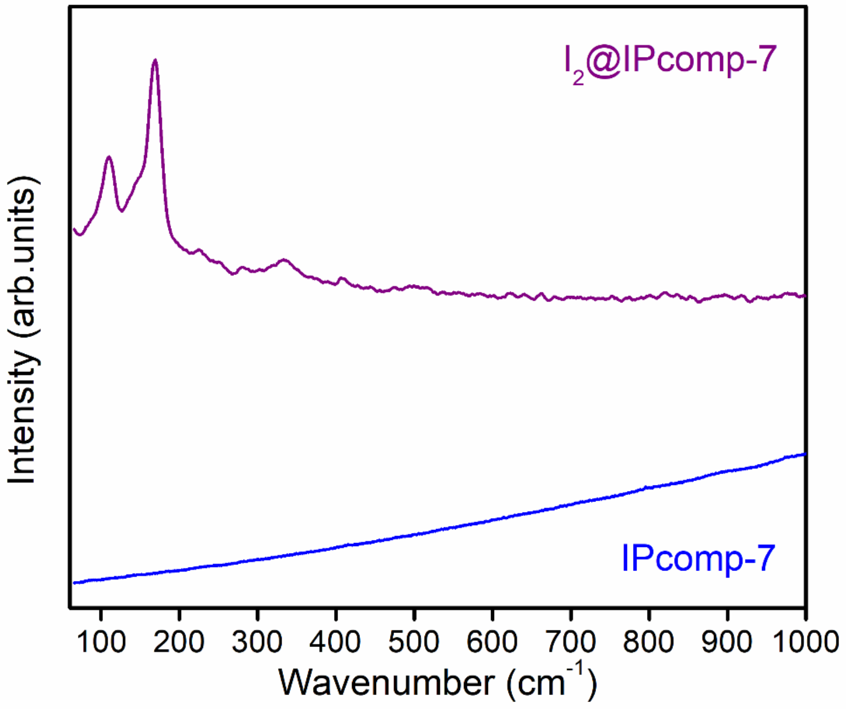

Supplementary Figure 88: Raman spectra of IPcomp-7 before and after I_2 absorption test.

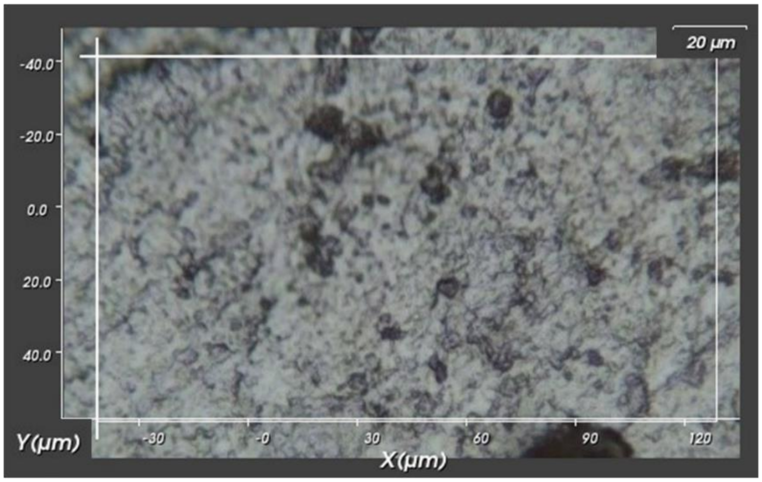

Supplementary Figure 89: Corresponding Raman spectroscopy image for mapping of IPcomp-7 after I_2 absorption test.

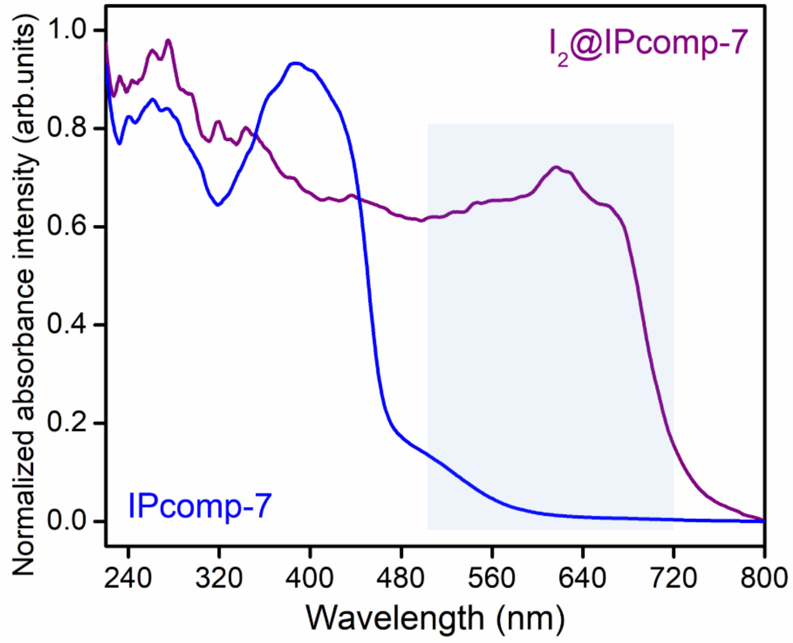

Supplementary Figure 90: Solid-state UV-vis spectra of IPcomp-7 before and after I_2 absorption test.

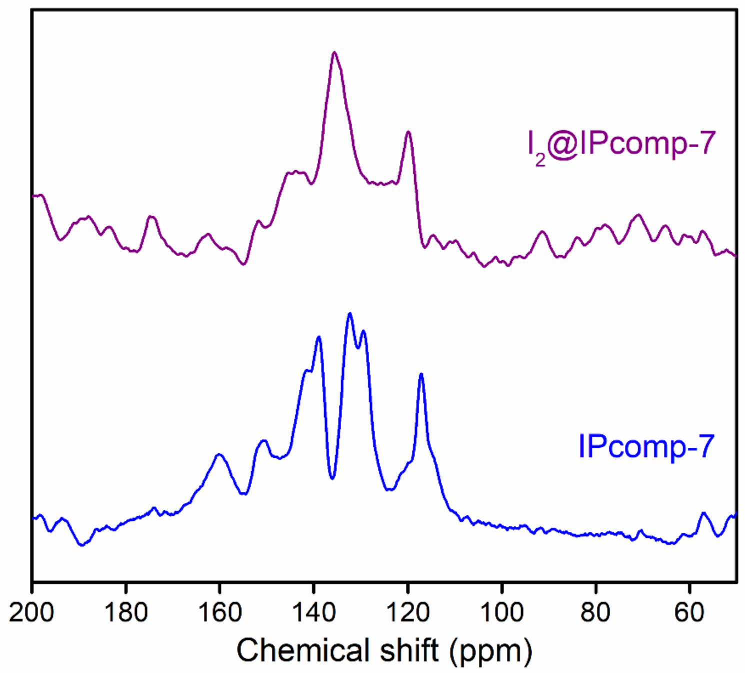

Supplementary Figure 91: Solid-state ^{13}C CP-MAS NMR spectra of IPcomp-7 before and after I_2 absorption test.

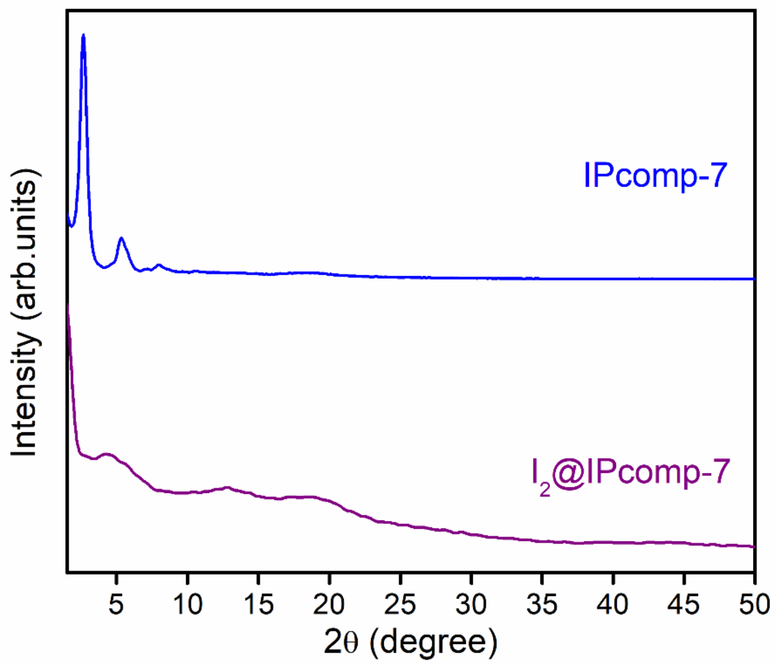

Supplementary Figure 92: PXRD profile of IPcomp-7 before and after I_2 absorption test.

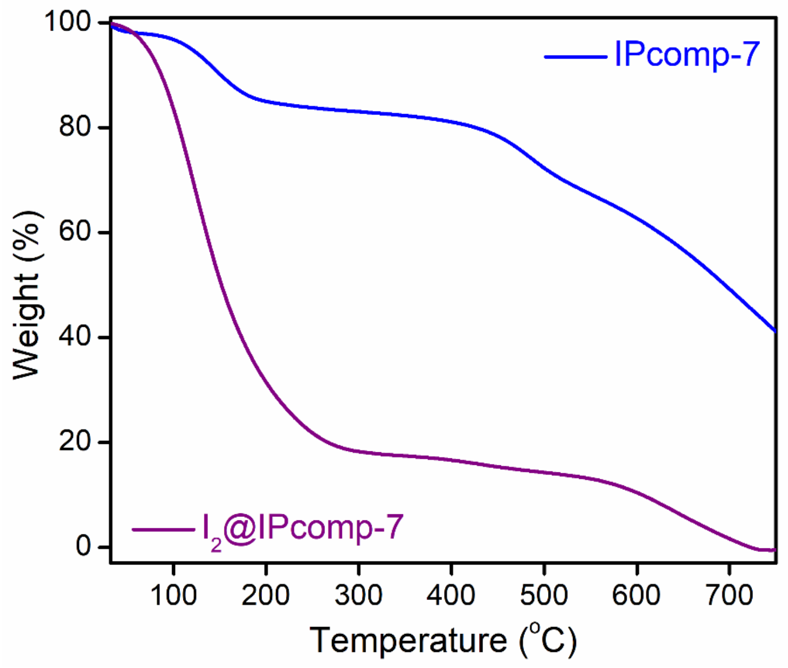

Supplementary Figure 93: TGA profile of IPcomp-7 before and after I_2 absorption test.

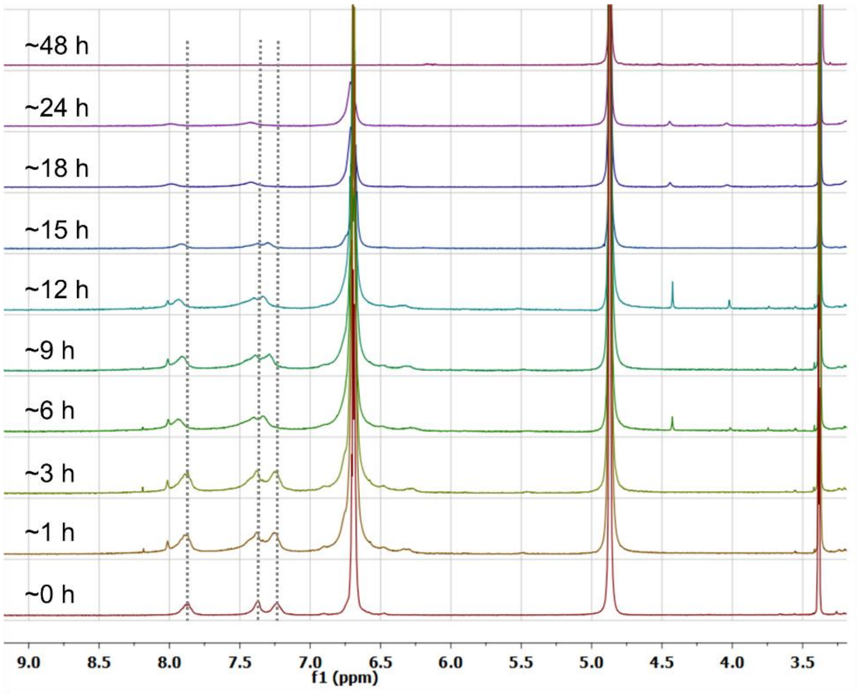

Supplementary Figure 94: Time-dependent ^{1}H NMR spectra of the stepwise treatment of I_2 to NH_2-Zr(IV)-MOP.

Supplementary Note 5

Confocal microscopy studies: Zeiss, Oberkochen, Germany Model LSM 710 has been used for background-free high resolution images of IPcomp-7. The instrument has EC Epiplan-APOCHROMAT objective of 50X magnification and a numerical aperture of 0.9. Fluorescence from the sample was excited with the 543-nm line of an He-Ne laser. The fluorescence was separated from the excitation light by band pass sliders (548 nm-703 nm) in front of spectral detectors. The microscope objective has been attached to an opto-electronically coded focus z-driver to scan the objective along the vertical z direction and produce three dimensional images. A pinhole was used to reject the out-of-plane light for background rejection hence better signal to noise ratio. We typically acquired 68 sliced stacks of 512 X 512 pixel images in the horizontal x–y plain separated by 0.6 um in the z direction. The length of the full-sized field of view is approximately 170 um and a depth approximately 42 μm. The images were processed using the Zen software (Zeiss, Oberkochen). Images of each plain were converted to 2.5 dimensional (2.5D) images to understand the absorbed dye intensity profile on the surface and internal microspores (graphical illustration of intensities per pixel for each plain using grayscale). Image processing software, ImageJ (Version 1.8.0) is used to generate volume-rendered three dimensional (3D) images of specimens.

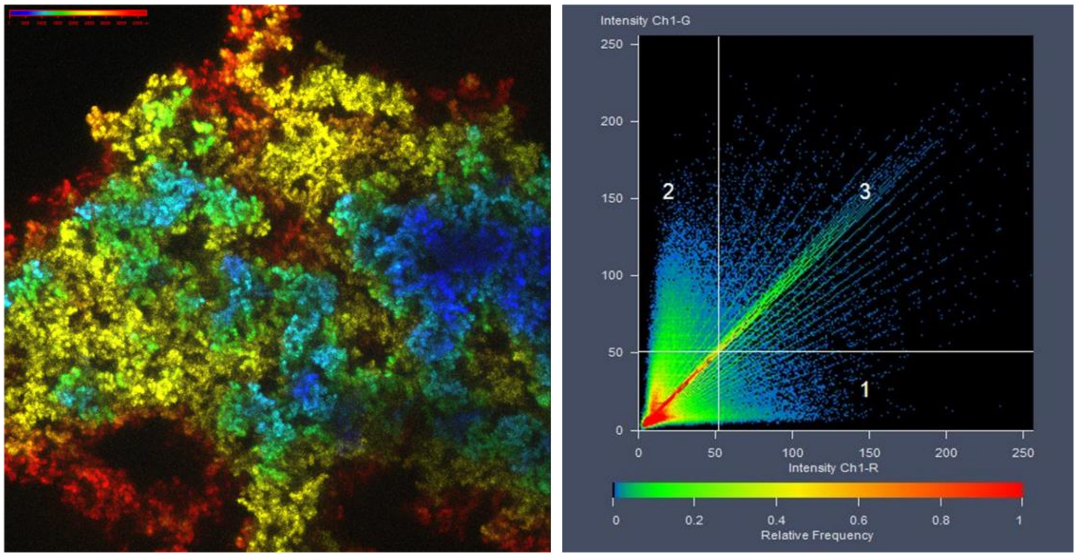

Supplementary Figure 95: Color-coded 3D confocal laser scanning microscopic images of IPcomp-7.

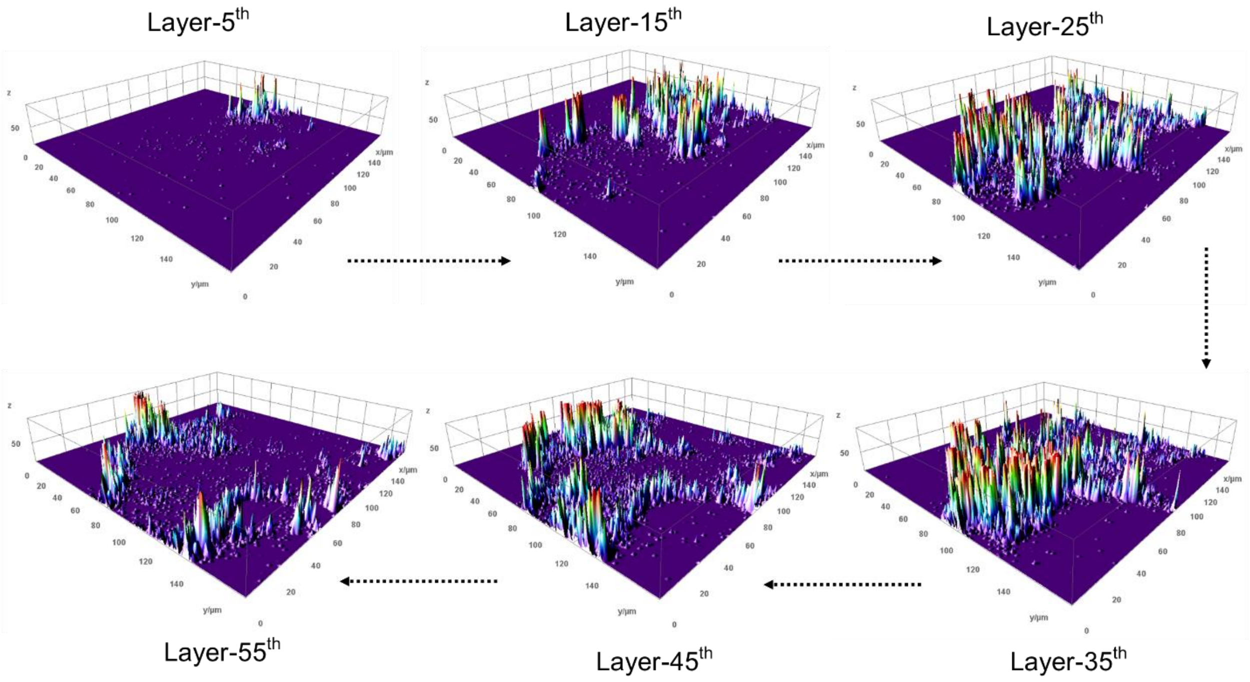

Supplementary Figure 96: 2.5 D confocal laser scanning microscopic processed images of different 2D layers of IPcomp-7 after treatment of dye.

Numerical simulation studies: Numerical simulation of mass transport properties in the hybrid aerogels were performed on their real 3D reconstructed model, using FlowDict® software package (GeoDict® 2018, Math2Market GmBH, Germany). Explicit jump solver is employed which follows Stokes equation, and are ideal for application in flows when the flow velocity dependence is linear. Using an appropriate numerical solver at 25°C, 20 Pa pressure-drop, etc., a simulation study was performed to visualize flow in the vertical axis (Y axis), and the outcome visualizes the average flow velocity profiles of the real 3D structure. Iterations in the range of 250 to 450 were undertaken with maximum error bound less than 0.8 %. Average flow velocity fields are calculated by explicit jump solver in the three directions by setting up the virtual pressure drop and selecting the computation directions or boundary conditions.

Considering the dynamic events taken place during the filtration process, these velocity profiles also predict the possible flow direction of I_2 stock solution through the stacked layers of the hybrid aerogel, in vertical axis (Y axis). Such events involving the transport of aqueous iodine solution, typically results in overall wetting of the hybrid aerogel walls with the solute. The process ultimately facilitates

the deposition or adsorption of solute across all the interconnected pores of the hybrid aerogel. Therefore, these velocity profiles itself may be considered as an effective evidence for the higher saturation of pore channels in the hybrid aerogels with iodine solution.

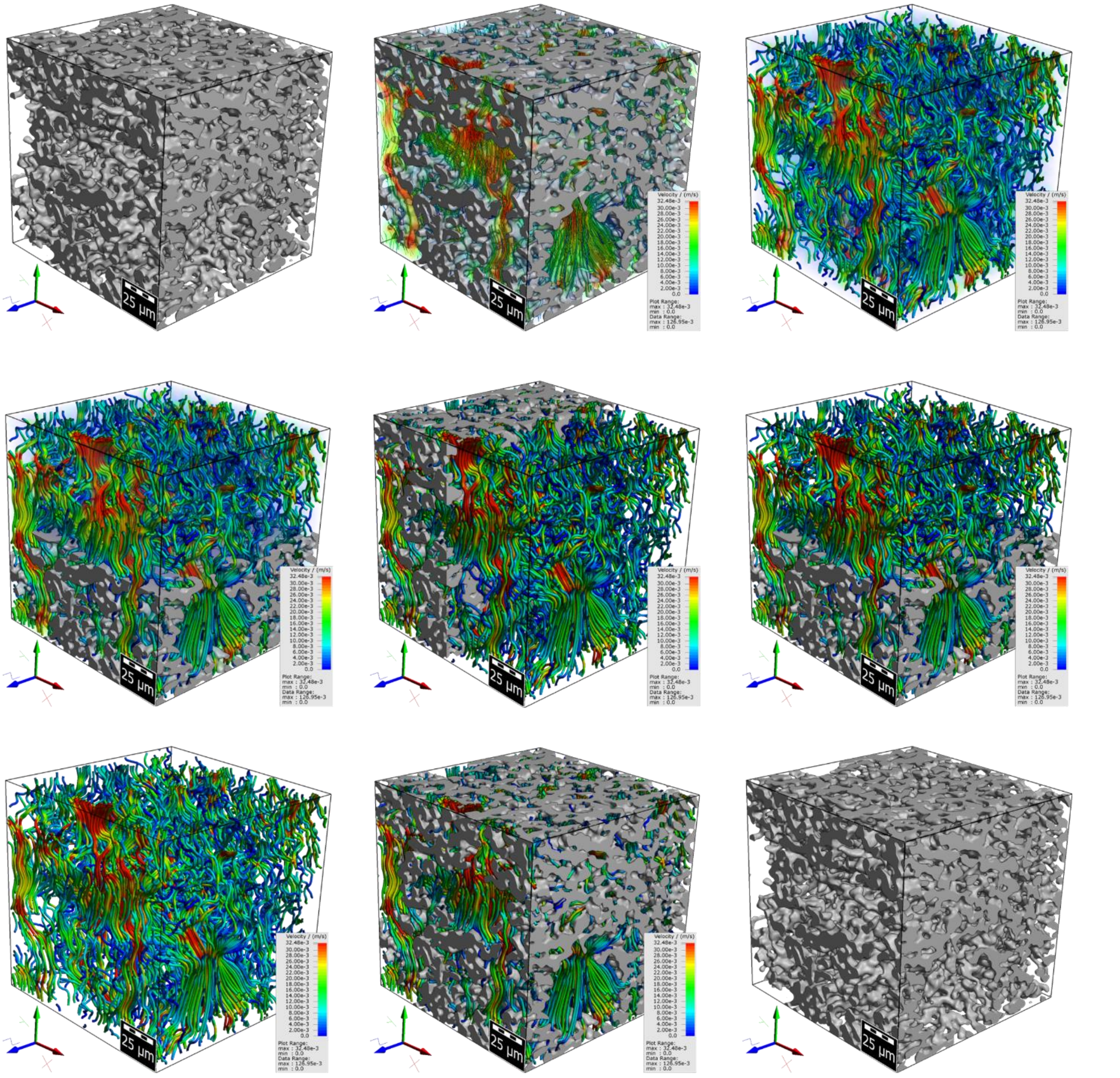

Supplementary Figure 97: The water flow velocity simulation analysis for IPcomp-7 by X-ray tomography. The average flow velocity has been calculated from the numerical simulation study.

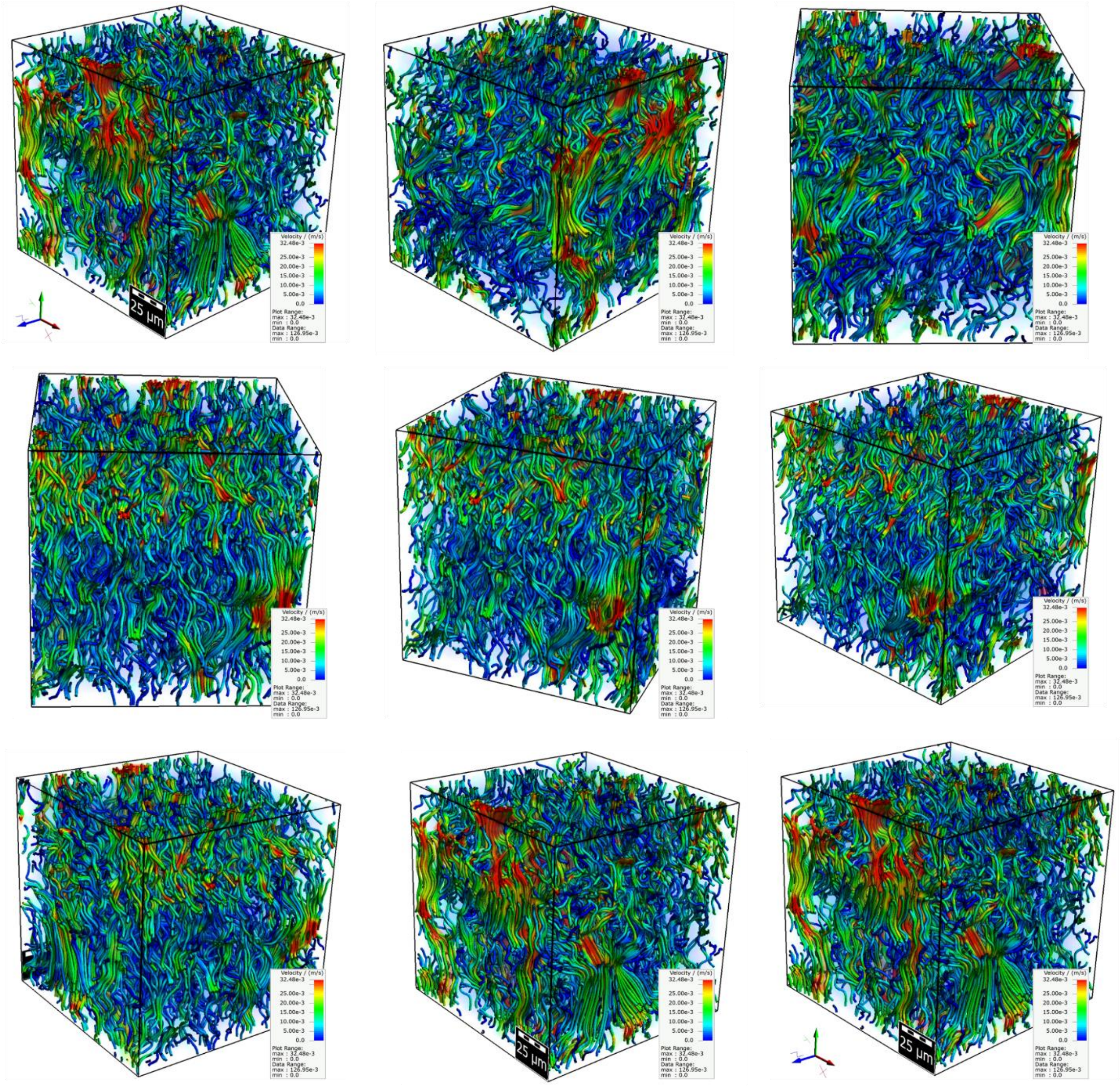

Supplementary Figure 98: The average water flow velocity simulation analysis in different direction for IPcomp-7 by X-ray tomography. The average flow velocity has been calculated from the numerical simulation study.

Supplementary Note 6

IPcomp-7 exhibited highly efficient selective uptake of iodine or triiodide anion from both vapor and aqueous phase. The multifunctional nature of the hybrid aerogel material is caused for such potent capture efficiencies. Among them, the hierarchical (micro-macro) porous structure of the composite material helps for fast mass diffusion of iodine/iodides through its large open pores towards the main active sites (guest- amino functionalized Zr(IV)-SBU-based cationic MOPs) of the hybrid composite. Thereafter, the iodine/iodides species interact with the Zr-SBU, free $-NH_2$ group and with the free chlorine anions of the cationic MOP. Such interactions are majorly attributed to the dispersive interactions or supramolecular interactions, dipole interactions of the iodine/iodides species (I_2/I_3^-) with the functional groups of MOP molecules of the composite material. To validate this, we have calculated the respective binding energies of I_2/I_3^- with the MOP molecules and unit of COF structure of the hybrid compound. The molecular-level interactions between the amino functionalized cationic MOP, imine functionalized 2D COF and iodine/iodides species were simulated using Discovery Studio 2017 (Accelrys). The DFT simulation was performed at fine quality calculation level. At first, using DMOL3 and B3LYP hybrid function the single unit of the MOP and COF was fully geometry relaxed by keeping the Multiplicity factor to Auto mode, double numeric plus polarizing (DNP+) basis set. Using simulated annealing technique structural simulation on the geometry relaxed MOP/COF (unit) structure was realized to find a feasible interaction site of the I_2/I_3^- within the relaxed MOP/COF unit. The electrostatic potential (ESP) on the van der Waals (VDW) surfaces (isodensity = 0.001 a.u.) of MOP/COF unit was derived from ground state electron density of the system. The DFT calculation reveals that I_2/I_3^- interact with Zr-SBU, free amine ($-NH_2$) groups, free Cl^- ions of the MOP and imine or other functional groups of COF unit with following major possible binding sites, (Supplementary Figure-99-105). Furthermore, we also optimized the Zr-SBU of the MOP interacting with H_2O and NO_2 molecules. These species are omnipresent with iodine vapor as major off-gas species. From the calculation, both the H_2O and NO_2 molecules were found to interact with terminal –OH groups of the Zr-SBU of the MOP, which have a strong tendency to form hydrogen bonding with H_2O and NO_2 molecules (Supplementary Figure-106-107).

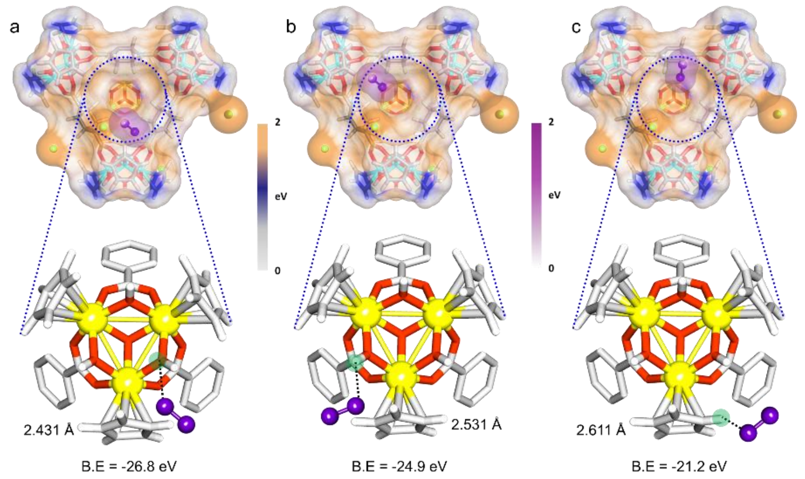

Supplementary Figure 99: Electrostatic Potential (ESP) diagram and corresponding DFT optimized structures with respective binding energies of different interaction between Zr-SBU of MOP in the hybrid composite with iodine molecule. (a) Interaction between iodine and oxygen of hydroxyl group of the Zr-SBU, (b) Interaction between iodine and hydrogen of hydroxyl group of the Zr-SBU, (c) Interaction between iodine and hydrogen of Cp-ring of the Zr-SBU, respectively. (Color code: zirconium: yellow, oxygen: red, carbon: gray, hydrogen: white, iodine: violet) (B.E.: binding energy).

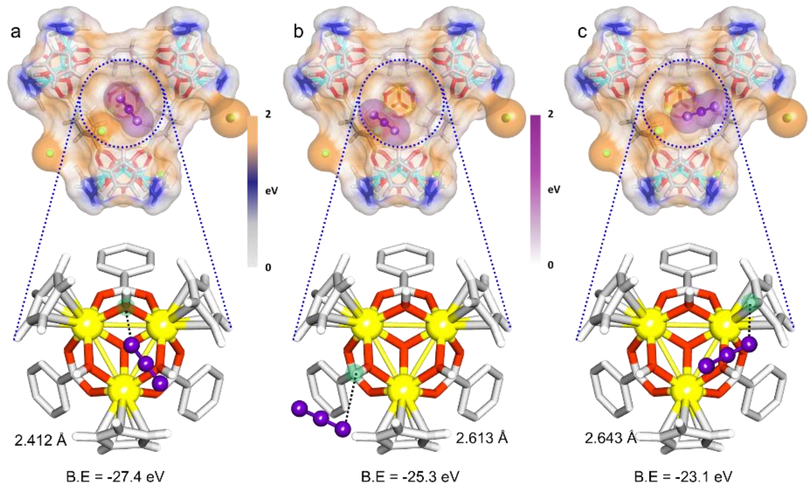

Supplementary Figure 100: Electrostatic Potential (ESP) diagram and corresponding DFT optimized structures with respective binding energies of different interaction between Zr-SBU of MOP in the hybrid composite with triiodide anions. (a) Interaction between triiodide and oxygen of hydroxyl group of the Zr-SBU, (b) Interaction between triiodide and hydrogen of hydroxyl group of the Zr-SBU, (c) Interaction between triiodide and hydrogen of Cp-ring of the Zr-SBU,

respectively. (Color code: zirconium: yellow, oxygen: red, carbon: gray, hydrogen: white, iodine: violet) (B.E.: binding energy).

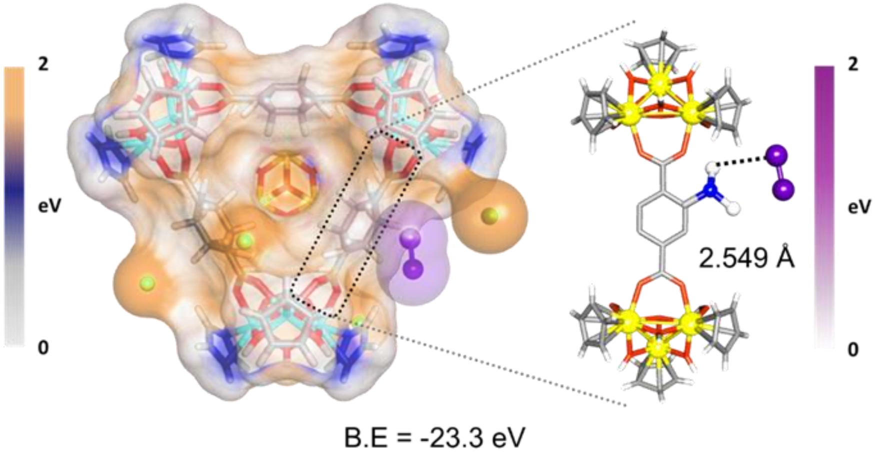

Supplementary Figure 101: Electrostatic Potential (ESP) diagram and corresponding DFT optimized structures with respective binding energies of interaction between -NH_2 groups of MOP in the hybrid composite with iodine molecule. (Color code: zirconium: yellow, oxygen: red, nitrogen: blue, carbon: gray, hydrogen: white, iodine: violet) (B.E.: binding energy).

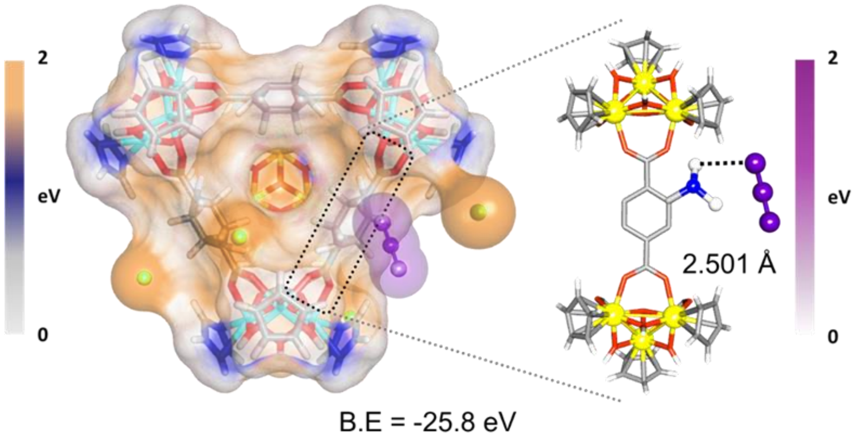

Supplementary Figure 102: Electrostatic Potential (ESP) diagram and corresponding DFT optimized structures with respective binding energies of interaction between –NH_2 groups of MOP in the hybrid composite with triiodide anions. (Color code: zirconium: yellow, oxygen: red, nitrogen: blue, carbon: gray, hydrogen: white, iodine: violet) (B.E.: binding energy).

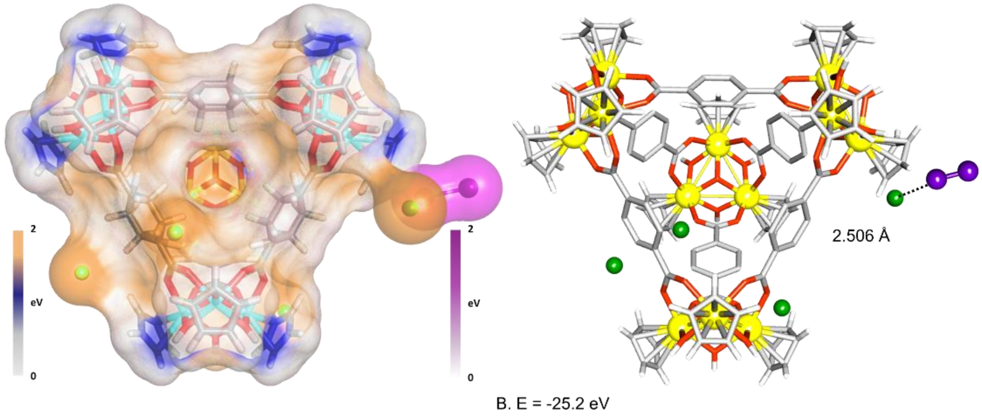

Supplementary Figure 103: Electrostatic Potential (ESP) diagram and corresponding DFT optimized structures with respective binding energies of interaction between free chlorine (Cl^-) anions of MOP in the hybrid composite with iodine molecule. (Color code: zirconium: yellow, oxygen: red, carbon: gray, hydrogen: white, chlorine: green, iodine: violet) (B.E.: binding energy).

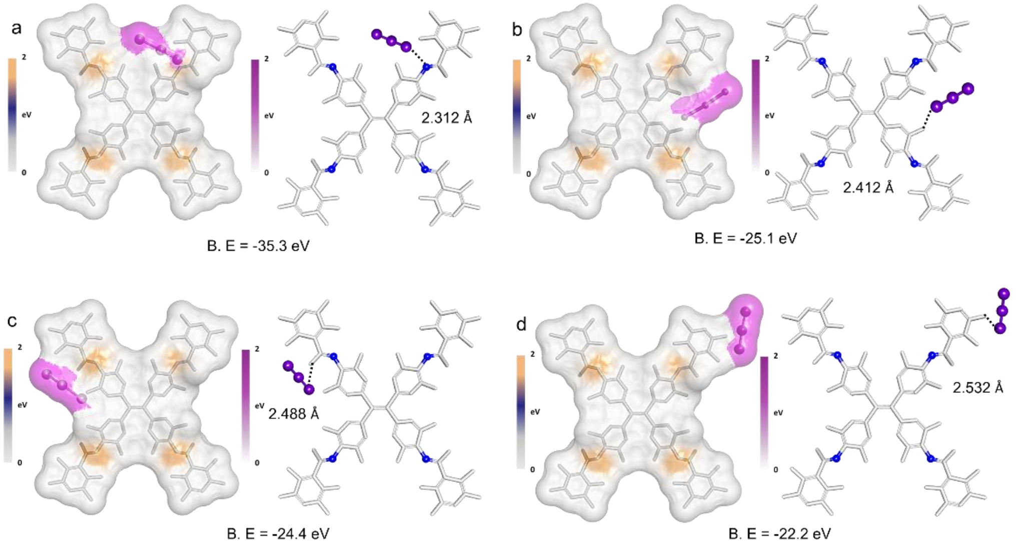

Supplementary Figure 104: Electrostatic Potential (ESP) diagram and corresponding DFT optimized structures with respective binding energies of different interaction between the TPE-core based COF units of the hybrid composite with iodine molecule. (a) Interaction between iodine and imine-N site of the COF, (b) Interaction between iodine and hydrogen of TPE-core of the COF, (c, d) Interaction between iodine and hydrogen of TPD-core of the COF, respectively. (Color code: nitrogen: blue, carbon: gray, hydrogen: white, iodine: violet) (B.E.: binding energy).

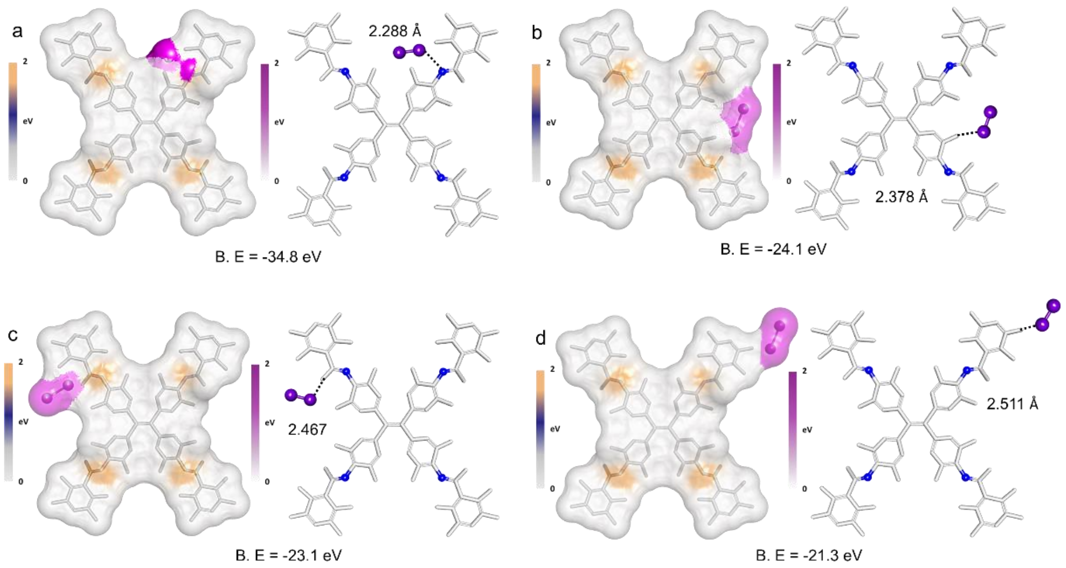

Supplementary Figure 105: Electrostatic Potential (ESP) diagram and corresponding DFT optimized structures with respective binding energies of different interaction between the TPE-core based COF units of the hybrid composite with triiodide anions. (a) Interaction between triiodide and imine-N site of the COF, (b) Interaction between triiodide and hydrogen of TPE-core of the COF, (c, d) Interaction between triiodide and hydrogen of TPD-core of the COF, respectively. (Color code: nitrogen: blue, carbon: gray, hydrogen: white, iodine: violet) (B.E.: binding energy).

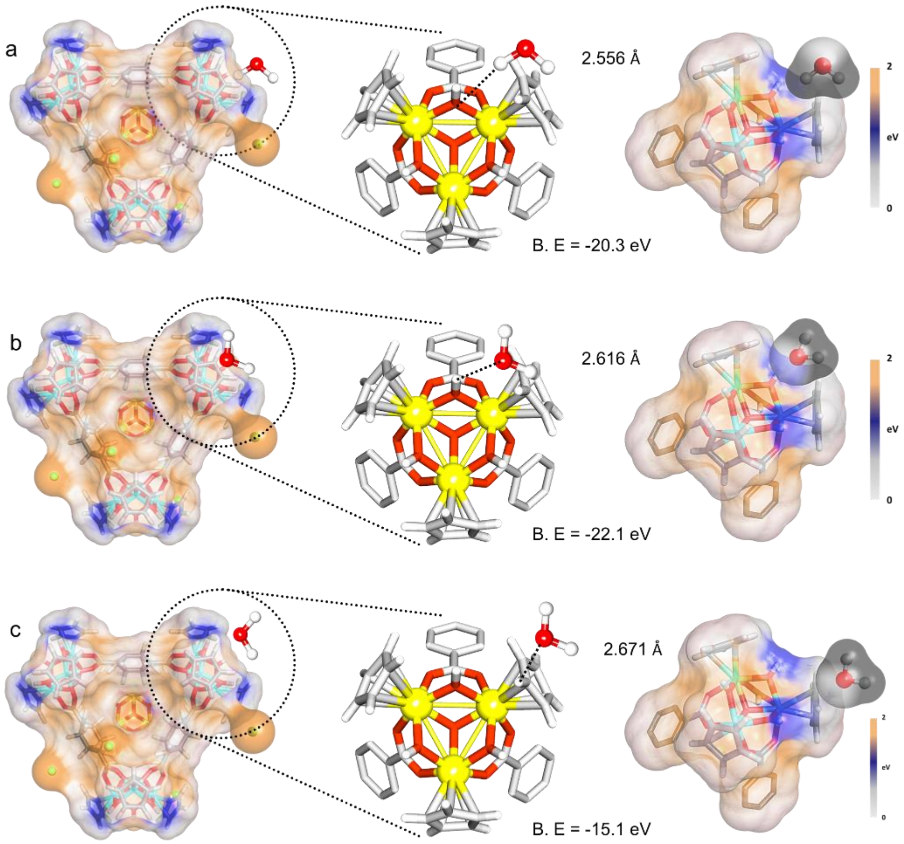

Supplementary Figure 106: Electrostatic Potential (ESP) diagram and corresponding DFT optimized structures with respective binding energies of different interaction between Zr-SBU of MOP in the hybrid composite with water molecule. (a) Interaction between hydrogen of water molecule and oxygen of hydroxyl group of the Zr-SBU, (b) Interaction between oxygen of water molecule and hydrogen of hydroxyl group of the Zr-SBU, (c) Interaction between oxygen of water molecule and hydrogen of Cp-ring of the Zr-SBU, respectively. (Color code: zirconium: yellow, oxygen: red, carbon: gray, hydrogen: white) (B.E.: binding energy).

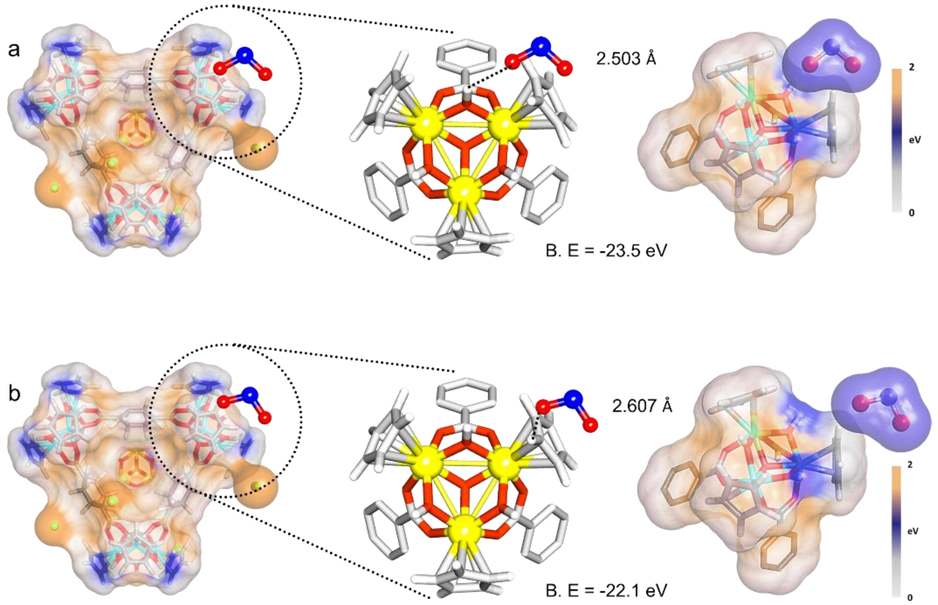

Supplementary Figure 107: Electrostatic Potential (ESP) diagram and corresponding DFT optimized structures with respective binding energies of different interaction between Zr-SBU of MOP in the hybrid composite with NO_2 molecule. (a) Interaction between oxygen of NO_2 molecule and hydrogen of hydroxyl group of the Zr-SBU, (b) Interaction between oxygen of NO_2 molecule and hydrogen of Cp-ring of the Zr-SBU, respectively. (Color code: zirconium: yellow, oxygen: red, nitrogen: blue, carbon: gray, hydrogen: white) (B.E.: binding energy).

Supplementary References

1. Liu, J., Duan, W., Song, J., Guo, X., Wang, Z., Shi, X., Liang, J., Wang, J., Cheng, P., Chen, Y., Zaworotko, M. J. & Zhang, Z. Self-Healing Hyper-Cross-Linked Metal−Organic Polyhedra (HCMOPs) Membranes with Antimicrobial Activity and Highly Selective Separation Properties. *J. Am. Chem. Soc.* **141**, 12064−12070 (2019).
2. Mollick, S., Fajal, S., Saurabh, S., Mahato, D. & Ghosh, S. K. Nanotrap Grafted Anion Exchangeable Hybrid Materials for Efficient Removal of Toxic Oxoanions from Water. *ACS Cent. Sci.* **6**, 1534–1541 (2020).
3. Fajal, S., Mandal, W., Mollick, S., More, Y. D., Torris, A., Saurabh, S., Shirolkar, M. M. & Ghosh, S. K. Trap Inlaid Cationic Hybrid Composite Material for Efficient Segregation of Toxic Chemicals from Water. *Angew. Chem. Int. Ed.* **61**, e202203385 (2022).
4. Xie, Y., Pan, T., Lei, Q., Chen, C., Dong, X., Yuan, Y., Shen, J., Cai, Y., Zhou, C., Pinnau, I. & Han, Y. Ionic Functionalization of Multivariate Covalent Organic Frameworks to Achieve an Exceptionally High Iodine-Capture Capacity. *Angew. Chem. Int. Ed.* **60**, 22432–22440 (2021).
5. Wang, P., Xu, Q., Li, Z., Jiang, W., Jiang, W. & Jiang, D. Exceptional Iodine Capture in 2D Covalent Organic Frameworks. *Adv. Mater.* **30**, 1801991 (2018).
6. An, S., Zhu, X., He, Y., Yang, L., Wang, H., Jin, S., Hu, J. & Liu, H. Porosity Modulation in Two-Dimensional Covalent Organic Frameworks Leads to Enhanced Iodine Adsorption Performance. *Ind. Eng. Chem. Res.* **58**, 10495 (2019).
7. Wang, C., Wang, Y., Ge, R., Song, X., Xing, X., Jiang, Q., Lu, H., Hao, C., Guo, X., Gao, Y. & Jiang, D. A 3D Covalent Organic Framework with Exceptionally High Iodine Capture Capability. *Chem.-Eur. J.* **24**, 585 (2018).
8. He, L., Chen, L., Dong, X., Zhang, S., Zhang, M., Dai, X., Liu, X., Lin, P., Li, K., Chen, C., Pan, T., Ma, F., Chen, J., Yuan, M., Zhang, Y., Chen, L., Zhou, R., Han, Y., Chai, Z. & Wang, S. A nitrogen-rich covalent organic framework for simultaneous dynamic capture of iodine and methyl iodide. *Chem* **7**, 699 (2021).
9. Chang, J., Li, H., Zhao, J., Guan, X., Li, C., Yu, G., Valtchev, V., Yan, Y., Qiu, S. & Fang, Q. Tetrathiafulvalene-based covalent organic frameworks for ultrahigh iodine capture. *Chem. Sci.* **12**, 8452-8457 (2021).
10. Guo, X., Li, Y., Zhang, M., Cao, K., Tian, Y., Qi, Y., Li, S., Li, K., Yu X. & Ma, L. Colyliform Crystalline 2D Covalent Organic Frameworks (COFs) with Quasi-3D Topologies for Rapid I_2 Adsorption. *Angew. Chem., Int. Ed.* **59**, 22697 (2020).
11. Yang, Y., Xiong, X., Fan, Y., Lai, Z., Xu, Z. & Luo, F. Insight into volatile iodine uptake properties of covalent organic frameworks with different conjugated structures. *J. Solid State Chem.* **279**, 120979 (2019).

12. Chen, R., Hu, T., Zhang, W., He, C. & Li, Y. Synthesis of nitrogen-containing covalent organic framework with reversible iodine capture capability. *Microporous Mesoporous Mater.* **312**, 110739 (2021).
13. Li, J., Zhang, H., Zhang, L., Wang, K., Wang, Z., Liu, G., Zhao, Y. & Zeng, Y. Two-dimensional covalent–organic frameworks for ultrahigh iodine capture. *J. Mater. Chem. A.* **8**, 9523 (2020).
14. Song, S., Shi, Y., Liu, N. & Liu, F. Theoretical Screening and Experimental Synthesis of Ultrahigh-Iodine Capture Covalent Organic Frameworks. *ACS Appl. Mater. Interfaces* **13**, 10513 (2021).
15. Yin, Z.-J., Xu, S.-Q., Zhan, T.-G., Qi, Q.-Y., Wu, Z.-Q. & Zhao, X. Ultrahigh volatile iodine uptake by hollow microspheres formed from a heteropore covalent organic framework. *Chem. Commun.* **53**, 7266–7269 (2017).
16. Jiang, X., Cui, X., Duncan, A. J., Li, L., Hughes, R. P., Staples, R. J., Alexandrov, E. V., Proserpio, D. M., Wu, Y. & Ke, C. Topochemical Synthesis of Single-Crystalline Hydrogen-Bonded Cross-Linked Organic Frameworks and Their Guest-Induced Elastic Expansion. *J. Am. Chem. Soc.* **141**, 10915–10923 (2019).
17. Xie, L., Zheng, Z., Lin, Q., Zhou, H., Ji, X., Sessler, J. L. & Wang, H. Calix[4]pyrrole-based Crosslinked Polymer Networks for Highly Effective Iodine Adsorption from Water. *Angew. Chem., Int. Ed.* **61**, e202113724 (2022).
18. Dai, D., Yang, J., Zou, Y.-C., Wu, J.-R., Tan, L.-L., Wang, Y., Li, B., Lu, T., Wang, B. & Yang. Y.-W. Macrocyclic Arenes-Based Conjugated Macrocycle Polymers for Highly Selective CO_2 Capture and Iodine Adsorption. *Angew. Chem., Int. Ed.* **60**, 8967 (2021).
19. Shetty, D., Raya, J., Han, D. S., Asfari, Z., Olsen, J.-C. & Trabolsi, A. Lithiated Polycalix[4]arenes for Efficient Adsorption of Iodine from Solution and Vapor Phases. *Chem. Mater.* **29**, 8968–8972 (2017).
20. Su, K., Wang, W., Li, B. & Yuan, D. Azo-Bridged Calix[4]resorcinarene-Based Porous Organic Frameworks with Highly Efficient Enrichment of Volatile Iodine. *ACS Sustain. Chem. Eng.* **6**, 17402 –17409 (2018).
21. Yan, Z., Yuan, Y., Tian, Y., Zhang, D. & Zhu, G. Highly Efficient Enrichment of Volatile Iodine by Charged Porous Aromatic Frameworks with Three Sorption Sites. *Angew. Chem., Int. Ed.* **54**, 12733 (2015).
22. Das, G., Prakasam, T., Nuryyeva, S., Han, D. S., Abdel-Wahab, A., Olsen, J.-C., Polychronopoulou, K., Plataslglesias, C., Ravaux, F., Jouiad, M. & Trabolsi, A. Multifunctional redox-tuned viologen-based covalent organic polymers. *J. Mater. Chem. A.* **4**, 15361 (2016).
23. Wang, S., Liu, Y., Ye, Y., Meng, X., Du, J., Song, X. & Liang, Z. Ultrahigh volatile iodine capture by conjugated microporous polymer based on N,N,N′,N′-tetraphenyl-1,4-phenylenediamine. *Polym. Chem.* **10**, 2608 (2019).

24. Li, H., Ding, X. & Han, B. H. Porous Azo-Bridged Porphyrin–Phthalocyanine Network with High Iodine Capture Capability. *Chem. Eur. J.* **22**, 11863 (2016).
25. Dang, Q.-Q., Wang, X.-M., Zhan, Y.-F. & Zhang, X.-M. An azo-linked porous triptycene network as an absorbent for CO_2 and iodine uptake. *Polym. Chem.* **7**, 643 (2016).
26. Subrahmanyam, K. S., Sarma, D., Malliakas, C. D., Polychronopoulou, K., Riley, B. J., Pierce, D. A., Chun, J. & Kanatzidis, M. G. Chalcogenide Aerogels as Sorbents for Radioactive Iodine. *Chem. Mater.* **27**, 2619 (2015).
27. Chen, Y., Sun, H., Yang, R., Wang, T., Pei, C., Xiang, Z., Zhu, Z., Liang, W., Li, A. & Deng, W. Synthesis of conjugated microporous polymer nanotubes with large surface areas as absorbents for iodine and CO_2 uptake. *J. Mater. Chem. A.* **3**, 87 (2015).
28. Sigen, A., Zhang, Y., Li, Z., Xia, H., Xue, M., Liu, X. & Mu, Y. Highly efficient and reversible iodine capture using a metalloporphyrin-based conjugated microporous polymer. *Chem. Commun.* **50**, 8495 (2014).
29. Ma, H., Chen, J.-J., Tan, L., Bu, J.-H., Zhu, Y., Tan, B. & Zhang, C. Nitrogen-Rich Triptycene-Based Porous Polymer for Gas Storage and Iodine Enrichment. *ACS Macro. Lett.* **5**, 1039 (2016).
30. Niu, T.-H., Feng, C.-C., Yao, C., Yang, W.-Y. & Xu, Y.-H. Bisimidazole-Based Conjugated Polymers for Excellent Iodine Capture. *ACS Appl. Polymer Materials* **3**, 354–361 (2020).
31. Geng, T., Zhang, C., Liu, M., Hu, C. & Chen, G. Preparation of biimidazole-based porous organic polymers for ultrahigh iodine capture and formation of liquid complexes with iodide/polyiodide ions. *J. Mater. Chem. A.* **8**, 2820–2826 (2020).
32. Janeta, M., Bury, W. & Szafert, S. Porous Silsesquioxane–Imine Frameworks as Highly Efficient Adsorbents for Cooperative Iodine Capture. *ACS Appl. Mater. Interfaces* **10**, 19964–19973 (2018).
33. Xu, M., Wang, T., Zhou, L. & Hua, D. Fluorescent conjugated mesoporous polymers with N,N-diethylpropylamine for the efficient capture and real-time detection of volatile iodine. *J. Mater. Chem. A.* **8**, 1966–1974 (2020).
34. Xiong, S., Tang, X., Pan, C., Li, L., Tang, J. & Yu, G. Carbazole-Bearing Porous Organic Polymers with a Mulberry-Like Morphology for Efficient Iodine Capture. *ACS Appl. Mater. Interfaces* **11**, 27335–27342 (2019).
35. Sava, D. F., Garino, T. J. & Nenoff, T. M. Iodine Confinement into Metal–Organic Frameworks (MOFs): Low-Temperature Sintering Glasses To Form Novel Glass Composite Material (GCM) Alternative Waste Forms. *Ind. Eng. Chem. Res.* **51**, 614 (2012).
36. Sava, D. F., Chapman, K. W., Rodriguez, M. A., Greathouse, J. A., Crozier, P. S., Zhao, H., Chupas, P. J. & Nenoff, T. M. Competitive I_2 Sorption by Cu-BTC from Humid Gas Streams. *Chem. Mater.* **25**, 2591 (2013).

37. Chen, P., He, X., Pang, M., Dong, X., Zhao, S. & Zhang, W. Iodine Capture Using Zr-Based Metal–Organic Frameworks (Zr-MOFs): Adsorption Performance and Mechanism. *ACS Appl. Mater. Interfaces* **12**, 20429–20439 (2020).
38. Tang, Y., Huang, H., Li, J., Xue, W. & Zhong, C. IL-induced formation of dynamic complex iodide anions in IL@MOF composites for efficient iodine capture. *J. Mater. Chem. A.* **7**, 18324–18329 (2019).
39. Valizadeh, B., N, T., Smit, B. & Stylianou, K. C. Porous Metal–Organic Framework@Polymer Beads for Iodine Capture and Recovery Using a Gas-Sparged Column. *Adv. Func. Mater.* **28**, 1801596 (2018).
40. Chapman, K. W., Chupas, P. J. & Nenoff, T. M. Radioactive Iodine Capture in Silver-Containing Mordenites through Nanoscale Silver Iodide Formation. *J. Am. Chem. Soc.* **132**, 8897 (2010).
41. Lin, Y., Jiang, X., Kim, S. T., Alahakoon, S. B., Hou, X., Zhang, Z., Thompson, C. M., Smaldone, R. A. & Ke, C. An Elastic Hydrogen-Bonded Cross-Linked Organic Framework for Effective Iodine Capture in Water. *J. Am. Chem. Soc.* **139**, 7172-7175 (2017).
42. Zhang, M., Samanta, J., Atterberry, B. A., Staples, R., Rossini, A. J. & Ke, C. A Crosslinked Ionic Organic Framework for Efficient Iodine and Iodide Remediation in Water. *Angew. Chem. Int. Ed.* **61**, e202214189, (2022).
43. An, D., Li, L., Zhang, Z., Asiri, A. M., Alamry, K. A. & Zhang, X. Amino-bridged covalent organic Polycalix[4]arenes for ultra efficient adsorption of iodine in water. *Mater. Chem. Phys.* **239**, 122328 (2020).
44. Gogia, A., Das, P. & Mandal, S. K. Tunable Strategies Involving Flexibility and Angularity of Dual Linkers for a 3D Metal–Organic Framework Capable of Multimedia Iodine Capture. *ACS Appl. Mater. Interfaces* **12**, 46107-46118 (2020).
45. He, T., Xu, X., Ni, B., Lin, H., Li, C., Hu, W. & Wang, X. Metal–Organic Framework Based Microcapsules. *Angew. Chem. Int. Ed.* **57**, 10148-10152 (2018).
46. Liu, Q.-K., Ma, J.-P. & Dong, Y.-B. Highly efficient iodine species enriching and guest-driven tunable luminescent properties based on a cadmium(ii)-triazole MOF. *Chem. Commun.* **47**, 7185-7187 (2011).
47. Li, B., Wang, B., Huang, X., Dai, L., Cui, L., Li, J., Jia, X. & Li, C. Terphen[n]arenes and Quaterphen[n]arenes (n=3–6): One-Pot Synthesis, Self-Assembly into Supramolecular Gels, and Iodine Capture. *Angew. Chem. Int. Ed.* **58**, 3885 (2019).
48. Li, X., Jia, Z., Zhang, J., Zou, Y., Jiang, B., Zhang, Y., Shu, K., Liu, N., Li, Y. & Ma, L. Moderate and Universal Synthesis of Undoped Covalent Organic Framework Aerogels for Enhanced Iodine Uptake. *Chem. Mater.* **34**, **24**, 11062–11071, (2022).
49. Sen, A., Sharma, S., Dutta, S., Shirolkar, M. M., Dam, G. K., Let, S. & Ghosh, S. K. Functionalized Ionic Porous Organic Polymers Exhibiting High Iodine Uptake from Both the Vapor and Aqueous Medium. *ACS Appl. Mater. Interfaces* **13**, 34188–34196 (2021).

50. Zhang, Z., Li, L., An, D., Li, H. & Zhang, X. Triazine-based covalent organic polycalix[4]arenes for highly efficient and reversible iodine capture in water *J. Mater. Sci.* **55**, 1854–1864 (2020).
51. Zhang, Q.-M., Zhai, T.-L., Wang, Z., Cheng, G., Ma, H., Zhang, Q.-P., Zhao, Y.-H., Tan, B. & Zhang, C. Hyperporous Carbon from Triptycene-Based Hypercrosslinked Polymer for Iodine Capture. *Adv. Mater. Interfaces* **6**, 1900249 (2019).
52. Cao, J., Zhu, H., Shangguan, L., Liu, Y., Liu, P., Li, Q., Wu, Y. & Huang, F. A pillar[5]arene-based 3D polymer network for efficient iodine capture in aqueous solution. *Polym. Chem.* **12**, 3517 (2021).
53. Xiong, S., Tao, J., Wang, Y., Tang, J., Liu, C., Liu, Q., Wang, Y., Yu, G. & Pan, C. Uniform poly(phosphazene–triazine) porous microspheres for highly efficient iodine removal. *Chem. Commun.* **54**, 8450 (2018).
54. Ren, F., Zhu, Z., Qian, X., Liang, W., Mu, P., Sun, H., Liu, J. & Li, A. Novel thiophene-bearing conjugated microporous polymer honeycomb-like porous spheres with ultrahigh iodine uptake. *Chem. Commun.* **52**, 9797-9800 (2016).
55. Jiang, Z.-Q., Wang, F. & Zhang. J. Adsorption of Iodine Based on a Tetrazolate Framework with Microporous Cages and Mesoporous Cages. *Inorg. Chem.* **55**, 13035-13038 (2016).
56. Yan, C. & Mu. T. Investigation of ionic liquids for efficient removal and reliable storage of radioactive iodine: a halogen-bonding case. *Phys. Chem. Chem. Phys.* **16**, 5071-5075 (2014).
57. BIOVIA, Dassault Systèmes, [Discovery Studio], [V16.1.0.15350], San Diego: Dassault Systèmes, (2017).
